# Nickel(ii) complexes with 14-membered bis-thiosemicarbazide and bis-isothiosemicarbazide ligands: synthesis, characterization and catalysis of oxygen evolution reaction†

**DOI:** 10.1039/d4dt02182g

**Published:** 2024-08-27

**Authors:** Iuliana Besleaga, Anastasia A. Fesenko, Anup Paul, Biljana Šljukić, Peter Rapta, Armando J. L. Pombeiro, Anatoly D. Shutalev, Vladimir B. Arion

**Affiliations:** a University of Vienna, Institute of Inorganic Chemistry Währinger Strasse 42 A-1090 Vienna Austria vladimir.arion@univie.ac.at; b A. N. Frumkin Institute of Physical Chemistry and Electrochemistry, Russian Academy of Sciences 31 Leninsky Ave. 119071 Moscow Russian Federation; c Centro de Química Estrutural, Institute of Molecular Sciences, Instituto Superior Técnico, Universidade de Lisboa Av. Rovisco Pais 1049-001 Lisboa Portugal pombeiro@tecnico.ulisboa.pt; d Center of Physics and Engineering of Advanced Materials, Laboratory of Physics of Materials and Emerging Technologies, Chemical Engineering Department, Instituto Superior Técnico, Universidade de Lisboa 1049-001 Lisbon Portugal; e Institute of Physical Chemistry and Chemical Physics, Faculty of Chemical and Food Technology, Slovak University of Technology in Bratislava Radlinského 9 SK-81237 Bratislava Slovak Republic peter.rapta@stuba.sk; f N. D. Zelinsky Institute of Organic Chemistry, Russian Academy of Sciences 47 Leninsky Ave. 119991 Moscow Russian Federation anatshu@gmail.com; g Inorganic Polymers Department, “Petru Poni” Institute of Macromolecular Chemistry Aleea Gr. Ghica Voda 41 A Iasi 700487 Romania

## Abstract

Design and development of novel, low-cost and efficient electrocatalysts for oxygen evolution reaction (OER) in alkaline media is crucial for lowering the reaction overpotential and thus decreasing the energy input during the water electrolysis process. Herein, we present the synthesis of new 14-membered bis-thiosemicarbazide and bis-isothiosemicarbazide macrocycles and their nickel(ii) complexes characterized by spectroscopic techniques (^1^H and ^13^C NMR, IR, UV–vis), electrospray ionization mass spectrometry, single crystal X-ray diffraction, scanning electron microscopy-energy dispersive X-ray spectroscopy (SEM-EDX) and cyclic voltammetry. Finally, the activity of nickel(ii) complexes towards OER is reported. Ni^II^L^SEt^ delivered a current density of 10 mA cm^−2^ at the lowest overpotential of 350 mV with the lowest Tafel slope of 93 mV dec^−1^. The high performance of Ni^II^L^SEt^ might be attributed to its high surface area and thus abundant active sites with the observed low charge-transfer resistance enabling the effective current flow through the electrocatalyst. Square-planar coordination geometry and increment in Ni oxidation state are believed to favor its OER performance. Beside high activity towards OER, Ni^II^L^SEt^ demonstrated excellent long-term stability with continuous operation, advocating its possible application in commercial systems.

## Abstract 2

The Ni(ii) complexes reported in this work show high performance for OER and are suggested as promising candidates for alkaline water electrolysis.

## Graphical abstract

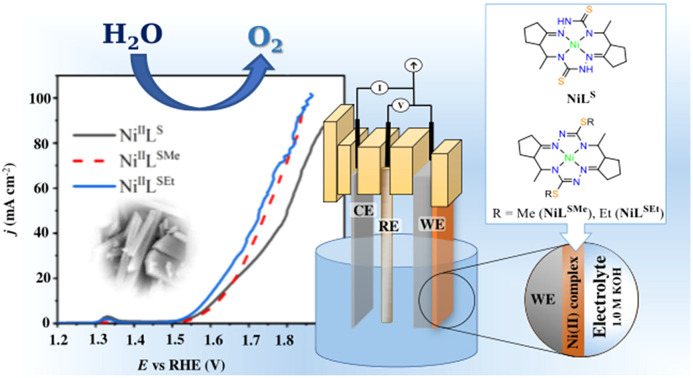

## Introduction

**Chart 1:**
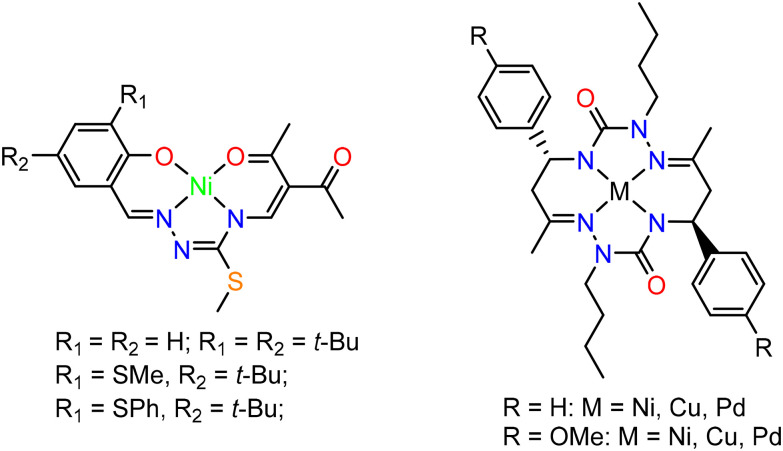
3d metal complexes with noninnocent ligands.

Open-chain and macrocyclic potentially tetradentate bis-isothiosemicarbazide ligands were first reported more than 25 years ago.^1,2^ These organic compounds were assembled in the presence of metal ions favoring square-planar coordination geometry, as a rule Ni(ii), but also Cu(ii), Co(ii), which in addition to square-planar coordination environment, could also accept 4 + 1 or 4 + 2 coordination geometry.^3,4^ Pentan-2,4-dione bis(S-alkylisothiosemicarbazones) were disclosed to act in some first row transition metal complexes as redox-active noninnocent ligands,^5^ adopt a series of protonation forms, as *i.e.*, neutral species,^6^ monoanions,^7^ dianions^8^ or even trianions,^9^ stabilize particular metal ions in unusually high oxidation states, *i.e.*, iron(iv),^10^ copper(iii),^11^ or undergo 2e oxidation with formation of 14π electronic species with a definite oxidation state.^12^ Nickel(ii) complexes with open-chain tetradentate ligands were also used as precursors for the assembly of macrocyclic complexes with both *cis*- and *trans*-arranged isothiosemicarbazide moieties when heated with acetylacetone (Hacac) and triethylorthoformate (CH(OEt)_3_) as both dehydrating and formylating agent.^13^ This kind of ligands is also quite reactive at central carbon atom of acac moiety and undergo dimerization through C–C bond formation^14^ or oxidation with formation of C=O group at the same carbon atom.^15^ Other related coordinated ligands exhibited similar reactivity.^16–19^ Redox noninnocent behavior was also disclosed for tetradentate ligands with N_2_O_2_ coordination environment^20^ and for some 14-membered hexaazamacrocyclic bis-semicarbazide ligands,^21^ shown in Chart 1.

The nickel(ii) complexes with tetradentate open-chain and 14-membered macrocyclic ligands can be in particular instances demetallated with isolation of free ligands^22,23^ used for coordination with other metal ions which are not suited as templates. This was not the case for nickel(ii) complexes with 14-membered hexaazamacrocycles based on 2,4-diketones and isothiosemicarbazides, which could not be demetalated due to their high thermodynamic stability and kinetic inertness.^13,24^

**Chart 2:**
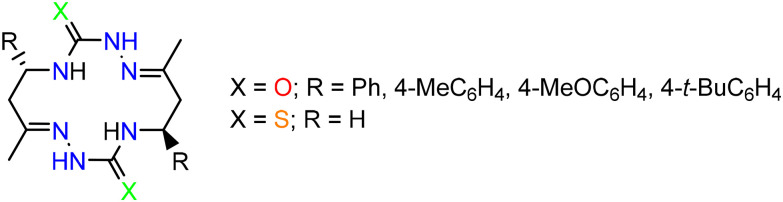
Macrocyclic compounds reported recently.

Quite recently, several new bis-semicarbazide^25,26^ and bis-thiosemicarbazide^27^ macrocycles were reported (Chart 2), which were self-assembled upon acid-promoted dimerization of particular hydrazones. The synthesis of these macrocycles offers opportunities for the discovery of unexplored so far aspects of their coordination chemistry and useful application of metal complexes.

**Chart 3:**
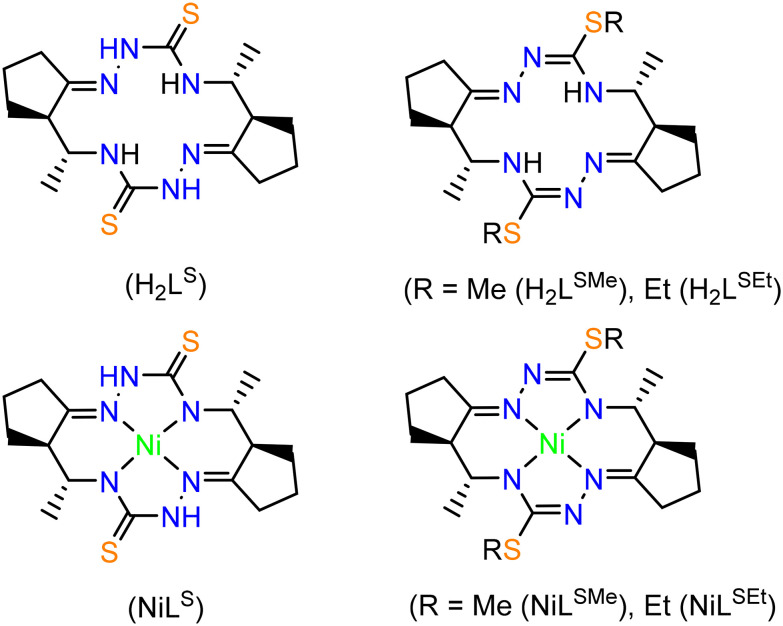
Compounds reported in this work.

Herein we report on the synthesis and comprehensive characterization of new 14-membered bis-thiosemicarbazide and bis-isothiosemicarbazide macrocycles and of their nickel(ii) complexes (Chart 3) by spectroscopic methods (^1^H and ^13^C NMR, IR, UV–vis), ESI mass spectrometry, single crystal X-ray diffraction (SC-XRD) and cyclic voltammetry. In contrast to metal complexes with open-chain chelating ligands, the complexes with polyazamacrocyclic ligands derived from isothiosemicarbazides are characterized by higher thermodynamic stability and kinetic inertness due to macrocyclic effect. Some of them remain intact in concentrated acids, *e.g.* HCl.

We also investigated the electrocatalytic activity of nickel(ii) complexes towards oxygen evolution reaction (OER) that plays a vital role in operation of energy conversion devices such as water electrolyzers, unitized regenerative fuel cells and rechargeable metal–air batteries.^28–30^ At the moment, electrocatalysts based on noble metal oxides IrO_2_ and RuO_2_ exhibit the best overall performance towards OER, but high costs hamper their use in commercial devices. Consequently, various non-noble metal OER electrocatalysts have been designed and developed, delivering promising performance.^29,31^ However, it is still challenging to achieve excellent performance at low overpotentials using these non-noble metal electrocatalysts which limits the overall efficiency of the above-mentioned devices. The high overpotential and consequent high energy input necessary to drive OER comes from the complexity of OER as a 4-electron transfer process. Thus, quest for novel, advanced electrocatalysts for OER continues. Use of transition metal complexes has been recently suggested, though often combined with highly conductive, high-surface area carbon nanostructured materials.^32–34^

## Results and discussion

### Synthesis of H_2_L^S^ and H_2_L^SEt^

**Scheme 1:**
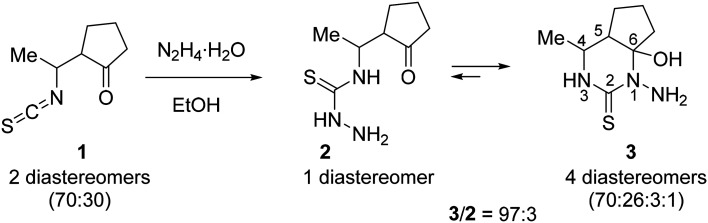
Synthesis of a 97 : 3 equilibrium mixture of pyrimidine 3 and its acyclic isomer 2.

Quite recently, we reported^27^ an unprecedented self-assembly of 14-membered bis-thiosemicarbazone and 28-membered tetrakis-thiosemicarbazone macrocycles *via* acid-promoted cyclooligomerization of 4-(3-oxobutyl)thiosemicarbazide hydrazone.^35^ We hypothesized that, under acidic conditions, hydrazones of other 4-(γ-oxoalkyl)thiosemicarbazides could be converted into new polyazamacrocyclic compounds as strong metal-binding chelators. However, the reaction of methyl-substituted β-isothiocyanatoketone 1 with hydrazine afforded thiosemicarbaside ketone 2 only as intermediate that spontaneously converted into its cyclic isomeric form, namely 1-amino-6-hydroxy-4-methyl-5,6-trimethylenehexahydropyrimidine-2-thione 3 (Scheme 1).^36^ At the same time, the ^1^H NMR spectrum of 3 revealed the presence of a small amount of the open-chain form 2. We envisioned that due to the ring-open chain isomerism this pyrimidine could react with an excess hydrazine to form the hydrazone of compound 2, which is expected to be a precursor to polyazamacrocycle.

The starting material, a 97 : 3 mixture of 3 and its acyclic isomer 2, was prepared in 82% yield by the reaction of freshly distilled isothiocyanato ketone 1 (two diastereomers, 70 : 30) with hydrazine hydrate in MeCN according to the reported procedure (Scheme 1).^36^ It should be noted that both recrystallization of the mixture from MeCN and standing of solution of this mixture in DMSO-*d*_6_ at room temperature for 13 days practically did not change the 3 : 2 ratio (Fig. S1 in the ESI†), which indicates that these isomers reached an equilibrium.

According to ^1^H NMR spectroscopic data, thiosemicarbazide 2 is a single diastereomer, while pyrimidine 3 is a mixture of 4 diastereomers with a significant predominance of two of them (70 : 26 : 3 : 1). Since the ratio of the two main isomers in 3 is approximately equal to the isomer ratio in the starting isothiocyanate 1 (see Scheme 1), it is obvious that these isomers differ in the relative configuration at the C4 and C5 atoms. The ^1^H NMR spectrum of 3 in DMSO-*d*_6_ showed that the major diastereomer had configuration 4R*,5S*,6R* with equatorial orientation of the Me group and axial positions of the OH group and the C5 substituent, providing a *cis*-junction of hexahydropyrimidine and cyclopentane rings.^37^ The first minor diastereomer of 3 (26%) had configuration 4R*,5R*,6R* with axial orientation of the OH group and equatorial positions of the Me group and the C5 substituent, providing a *trans*-junction of hexahydropyrimidine and cyclopentane rings.^38^

**Scheme 2:**
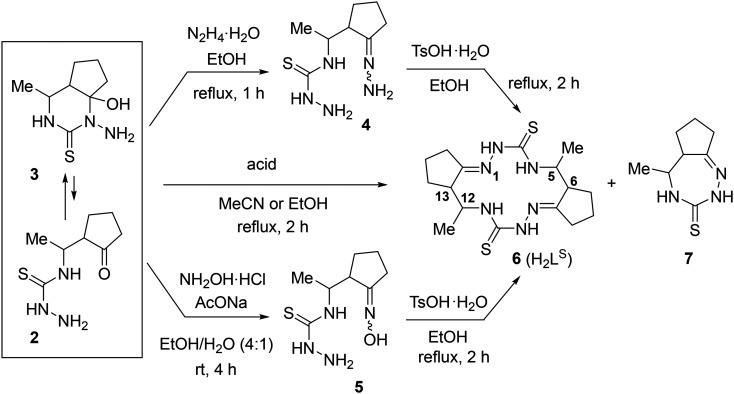
Synthesis of 14-membered cyclic bis-thiosemicarbazone 6 (H_2_L^S^).

The prepared equilibrium mixture of pyrimidine 3 and its acyclic isomer 2 was used for the synthesis of the two most suitable macrocycle precursors, namely, hydrazone and oxime of 4-[1-(2-oxocyclopentyl)ethyl]thiosemicarbazide (4 and 5, respectively, in Scheme 2).

We found that the 3 + 2 mixture readily and completely reacts with excess hydrazine hydrate in refluxing EtOH to give the expected hydrazone 4 as an oil. After many failed attempts to solidify the hydrazine 4, we finally succeeded to isolate it as a hygroscopic solid foam by extracting the oily product with chloroform, washing the extract with brine and evaporating the solvent under reduced pressure.^39^

According to ^1^H NMR spectrum, hydrazone 4 was formed as a mixture of three stereoisomers in a ratio of 46 : 45 : 9. Since we were unable to obtain this compound in analytically pure form due to its high hygroscopicity and lability, we used it in the cyclization step without additional purification.

Oxime 5 was prepared as a hygroscopic solid foam by reaction of the 3 + 2 mixture with NH_2_OH·HCl in the presence of NaOAc in EtOH/H_2_O at room temperature followed by work up (see Experimental part). ^1^H NMR spectrum of 5 in DMSO-*d*_6_ showed that it was a mixture of four stereoisomers in a ratio of 55 : 26 : 11 : 7. This product was also used in the macrocyclization step without further purification.

The macrocyclization of hydrazone 4 and oxime 5 was performed in refluxing EtOH by using TsOH·H_2_O as an acidic promoter (Scheme 2). In contrast to the cyclization of hydrazone of 4-(3-oxobutyl)thiosemicarbazide which, under similar conditions, was converted into a 92 : 8 mixture of 14- and 28-membered polyazamacrocycles,^27^ hydrazone 4 was cyclized with exclusively high selectivity to give only the 14-membered cyclic bis-thiosemicarbazone 6 (H_2_L^S^). This product precipitated from the reaction mixture and was isolated in 83% yield and high purity (>98%) (Table 1, entry 1). Although the starting material was a mixture of stereoisomers (*vide supra*) and there were 4 stereocenters in the macrocycle 6, it was formed with excellent stereoselectivity. According to ^1^H NMR spectrum, the crude macrocycle 6 was a mixture of several diastereomers with a huge predominance of the isomer with the configuration 5R*,6R*,12R*,13R* (≥94 mol%). Under similar conditions, oxime 5 was converted into macrocycle 6 with very high stereoselectivity, but with a lower yield (64%, entry 2).

**Table 1:** Synthesis of 14-membered cyclic bis-thiosemicarbazone 6 a

Entry	Substrate^b^	Promoter (equiv)	Solvent	Conc. of substrate mmol mL^−1^	Products distribution^c^ (%)		Isolated yield (%)
					6 d	7 (isomer ratio)	
1	4	TsOH·H_2_O (1.10)	EtOH	0.35	100	0	83
2	5	TsOH·H_2_O (1.12)	EtOH	0.18	100	0	64
3	3 + 2	NH_2_OH·HCl (1.25)	EtOH	0.30	100	0	92
4	3 + 2	NH_2_OH·HCl (1.26)	MeCN	0.25	82	18 (72 : 28)	88
5	3 + 2	NH_2_OH·HCl (0.75)	EtOH	0.23	95	5 (63 : 37)	89
6	3 + 2	NH_2_OH·HCl (0.26)	EtOH	0.21	78	22 (71 : 29)	87
7	3 + 2	NH_2_C(O)NHNH_2_.HCl (1.27)	EtOH	0.25	90	10 (63 : 37)	87
8	3 + 2	Pyridine (1.27), TsOH·H_2_O (1.25)	EtOH	0.32	28	72 (85 : 15)	86
9	3 + 2	AcOH (1.25)	EtOH	0.27	Traces	100 (37 : 63)	—e

a All reactions were carried out in EtOH or MeCN under reflux for 2 h.

b The starting material was either a 97 : 3 mixture of compounds 3 and 2, or hydrazone 4 (solid foam), or oxime 5 (solid foam) (see the discussion).

c According to ^1^H NMR spectroscopic data for the crude products.

d The major stereoisomer (90–94 mol%) with (7*E*,14*E*)-(5R*,6R*,12R*,13R*)-configuration plus a set of small amounts of other stereoisomers (total 6–10 mol% according to ^1^H NMR data).

e Not less than 35% unidentified by-products was also formed (^1^H NMR data).

Despite the successful synthesis of 6 from hydrazone 4 and oxime 5, the use of these starting materials was inconvenient due to their high hygroscopicity and the inability to reliably purify them (*vide supra*). We hypothesized that *in situ* generation of these compounds from the 3 + 2 mixture in the presence of acid could lead to the formation of the target macrocycle 6. Indeed, treatment of the 97 : 3 mixture of 3 and 2 with hydroxylamine hydrochloride in refluxing EtOH produced the bis-thiosemicarbazone 6 in 92% yield and with high diastereoselectivity (entry 3). Apparently, the first step of this transformation involves deprotonation of NH_2_OH·HCl in the presence of thiosemicarbazides 2 or/and 3 as bases followed by reaction of NH_2_OH formed with 2, which is in equilibrium with 3, to afford oxime 5. Then, the two molecules of oxime 5 react with each other and the obtained dimeric species undergoes cyclization to give 6.

The successful NH_2_OH·HCl promoted synthesis of 6 prompted us to study the macrocyclization of the 3 + 2 mixture using other promoters and other reaction conditions. However, all the results obtained were worse than those described above. For example, under the above conditions, but with MeCN as a solvent, a 82 : 18 mixture of macrocycle 6 and triazepinethione 7 (two diastereomers, 72 : 28) was prepared (entry 4). Decrease of the amount of NH_2_OH·HCl from 1.25 equiv (entry 4) to 0.75 equiv (entry 5), and then to 0.26 equiv (entry 6) also reduced the reaction selectivity (6/7 = 100 : 0, 95 : 5, and 78 : 22, respectively). Semicarbazide hydrochloride was shown to be a less selective promoter than hydroxylamine hydrochloride (entry 7 *vs*. entry 3). Pyridinium tosylate also catalyzed the macrocyclization of the 3 + 2 mixture, but the formed product contained 72% of triazepinethione 7 and only 28% of macrocycle 6 (entry 8). The use of AcOH as a promoter resulted in the predominant formation of triazepinethione 7 (entry 9).

Thus, very convenient synthesis of 14-membered macrocycle 6 (H_2_L^S^) involving treatment of readily available isomeric mixture of compounds 3 and 2 with NH_2_OH·HCl was developed. Macrocycle was formed in excellent yield and purity, including diastereomeric purity (*vide supra*). It should be noted that we scaled up this reaction to a 40.8 mmol loading (multi-gram scale) with no loss of product purity and even a slight increase in yield (up to 97%).

It should be also stressed that although the starting material, pyrimidine 3, was mainly a mixture of (4R*,5S*,6R*)- and (4R*,5R*,6R*)-diastereomers in a ratio of 70 : 26, macrocycle 6 was mainly obtained as the only diastereomer with the configuration 5R*,6R*,12R*,13R*. This nontrivial result can be explained by epimerization of (R*,S*)-isomers in isomeric mixtures of the acyclic intermediates (*e.g.*, oxime 5) under reaction conditions to the corresponding (R*,R*)-isomers, followed by dimerization of the latter and cyclization. It is also of note that the dimerization proceeds highly selectively involving molecules with the same relative configuration of the stereocenters to give predominantly (5R*,6R*,12R*,13R*)-diastereomer of macrocycle 6, but not its (5R*,6R*,12S*,13S*)-diastereomer. Apparently, the rate of the dimerization of molecule pair with the same relative configurations (*R*,*R* + *R*,*R* or *S*,*S* + *S*,*S*) is essentially higher than that of molecule pair with the opposite relative configurations (*R*,*R* + *S*,*S* or *S*,*S* + *R*,*R*). It should be mentioned, however, that in addition to the (5R*,6R*,12R*,13R*)-isomer, small amounts of some other stereoisomers are also formed, but their total content did not exceed 10% (Fig. S2 in the ESI†). After recrystallization of the crude product 6 from *n*BuOH, the major diastereomer was isolated in pure form.

The structure of (5R*,6R*,12R*,13R*)-6 was unambiguously confirmed by 1D and 2D NMR spectra (Fig. S3A–S3H in the ESI†), ESI mass spectrometry (Fig. S5 and S6 in the ESI†), elemental analysis, infrared spectra (Fig. S8 in the ESI†), as well as by single crystal X-ray diffraction (*vide infra*). In ESI (+) mass spectrum a characteristic peak with *m*/*z* 367.25 was attributed to the ion [H_2_L^S^ + H]^+^ (Fig. S5 in the ESI†), while in the ESI (−) spectrum the peak with *m*/*z* 365.17 was attributed to the ion [H_2_L^S^-H]^−^ (Fig. S6 in the ESI†). The number of resonances in ^1^H and ^13^C{1H} NMR spectra of (5R*,6R*,12R*,13R*)-6 is in agreement with *C*_2_ molecular symmetry of the compound in solution (Fig. S3A in the ESI†). A high value (11.0 Hz) of vicinal coupling between the H-5 and H-6 protons and between the H-12 and H-13 protons provides evidence that they are pairwise antiperiplanar, and therefore, this compound adopts *trans*-arrangement of substituents at the C-5 and C-6 atoms, as well as at the C-12 and C-13 atoms. These data and the results of the NOESY experiment (Fig. S3H in the ESI†) indicated (5R*,6R*,12R*,13R*)-configuration of the isolated macrocycle.

**Scheme 3:**
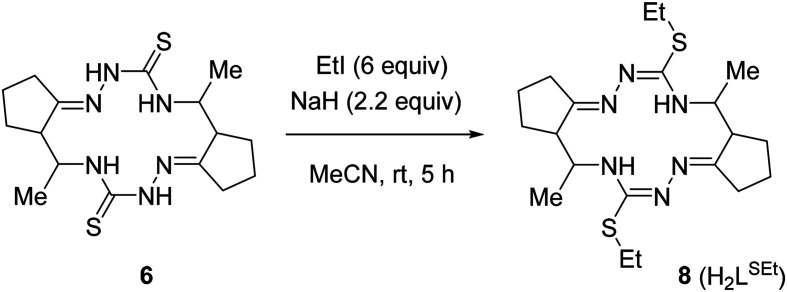
Synthesis of 14-membered cyclic bis-isothiosemicarbazone 8 (H_2_L^SEt^).

The crude macrocycle 6 was further alkylated by reaction with excess iodoethane in the presence of NaH in dry MeCN at room temperature to give the desired 14-membered bis-isothiosemicarbazone 8 (H_2_L^SEt^) as a diastereomeric mixture with a strong predominance of (7*E*,14*E*)-(5R*,6R*,12R*,13R*)-diastereomer (≥90%) in 98% yield (Scheme 3). Recrystallization of the crude 8 from EtOH afforded this diastereomer in analytically pure form.

The structure of (5R*,6R*,12R*,13R*)-8 was unambiguously established by 1D and 2D NMR spectroscopy (Fig. S4A–S4M in the ESI†), ESI mass spectrometry (Fig. S7 in the ESI†), infrared spectra (Fig. S9 in the ESI†), elemental analysis, as well as by single crystal X-ray diffraction (*vide infra*). The bis-isothiosemicarbazide macrocycle H_2_L^SEt^ showed in the positive ion mass spectra a peak with *m*/*z* 423.25 corresponding to the ion [H_2_L^SEt^ + H]^+^ (Fig. S7 in the ESI†). The ^1^H and ^13^C{1H} NMR spectra of this compound are in agreement with *C*_2_ molecular symmetry of 8 in solution. Like for 6, a high value (10.3 Hz) of vicinal coupling between the H-5 and H-6 protons and between the H-12 and H-13 protons indicates that these protons are pairwise antiperiplanar. The presence of vicinal coupling (3.4 Hz) of the H-5 and H-12 protons with the N_(4)_H and N_(13)_H protons, respectively, indicates that the tautomeric structures of the two isothiosemicarbazone fragments correspond to those presented in Scheme 3.

### Synthesis of Ni(ii) complexes

By heating the reaction mixture of H_2_L^S^ and NiCl_2_·6H_2_O in DMF in the presence of Et_3_N in 1 : 1 : 2 molar ratio the complex NiL^S^ was isolated as red microcrystalline product in 53% yield. The ESI mass spectrum measured in positive ion mode (Fig. S10 in the ESI†) showed a peak with *m*/*z* 423.16 and 445.11 attributed to [NiL^S^ + H]^+^ and [NiL^S^ + Na]^+^ respectively, while that recorded in the negative ion mode (Fig. S11 in the ESI†) revealed a peak with *m*/*z* 421.00 assigned to [NiL^S^-H]^−^. The complex is diamagnetic. The number of resonances in ^1^H and ^13^C NMR spectra (Fig. S12 and S13 in the ESI†) is in accord with its *C*_2_-molecular symmetry in solution. IR and UV–vis spectra of NiL^S^ are presented in Fig. S14 and S15 in the ESI.†

The bis-isothiosemicarbazide macrocycle H_2_L^SMe^ was generated by treatment of H_2_L^S^ with NaH in dry DMF under argon and subsequent addition of iodomethane. The ligand generated *in situ* was further reacted with Ni(OAc)_2_·4H_2_O to give a red microcrystalline product of NiL^SMe^ in 53% yield. The methylation of both thione sulfur atoms in H_2_L^S^ was confirmed by ESI (+) mass spectrum (Fig. S16 in the ESI†), in which a peak with *m*/*z* 451.21 was observed, which could be easily attributed to the ion [NiL^SMe^ + H]^+^. In ESI (−) mass spectrum (Fig. S17 in the ESI†), a peak with *m*/*z* 449.02 was assigned to the ion [NiL^SMe^-H]^−^. The SMe protons were seen at 2.49 ppm, while the ^13^C resonance of these two groups at 16.35 ppm. As for H_2_L^S^, the number of resonances in ^1^H and ^13^C NMR spectra (Fig. S18 and S19 in the ESI†) is in agreement with its *C*_2_-molecular symmetry. Fig. S20 and S21 in the ESI† show the IR and UV–vis spectra of NiL^SMe^.

In contrast to NiL^SMe^, the complex NiL^SEt^ was prepared by direct reaction of H_2_L^SEt^ with Ni(OAc)_2_·4H_2_O in the presence of 2 equiv of Et_3_N in dry and anoxic DMF in 63% yield. The complex formation was evidenced by mass spectra, in which the peak with *m*/*z* 479.20 was attributed to [NiL^SEt^ + H]^+^ in positive mode, while that with *m*/*z* 477.06 in the negative ion mode to [NiL^SEt^-H]^−^ (Fig. S22 and S23 in the ESI†). The ^1^H and ^13^C NMR spectra (Fig. S24 and 25 in the ESI†) provided further evidence for the formation of NiL^SEt^ and its *C*_2_-molecular symmetry in solution. Other spectroscopic data for NiL^SEt^ are displayed in Fig. S26 and S27 in the ESI.† The optical spectra of NiL^S^, NiL^SMe^ and NiL^SEt^ in the visible region of the spectrum are characterized by low intensity d–d band with maxima at 526, 530 and 529 nm, respectively (Fig. S15, S21 and S27 in the ESI†), in accord with their square-planar coordination geometry for low-spin d^8^ electronic configuration (*S* = 0).

In addition, single crystals of X-ray diffraction quality of NiL^S^ and NiL^SEt^ were obtained from mother liquor in a Schlenk tube upon standing at +4 °C, while those of NiL^SMe^ by diffusion of diethyl ether into the mother liquor at room temperature.

#### X-ray crystallography

**Fig. 1:**
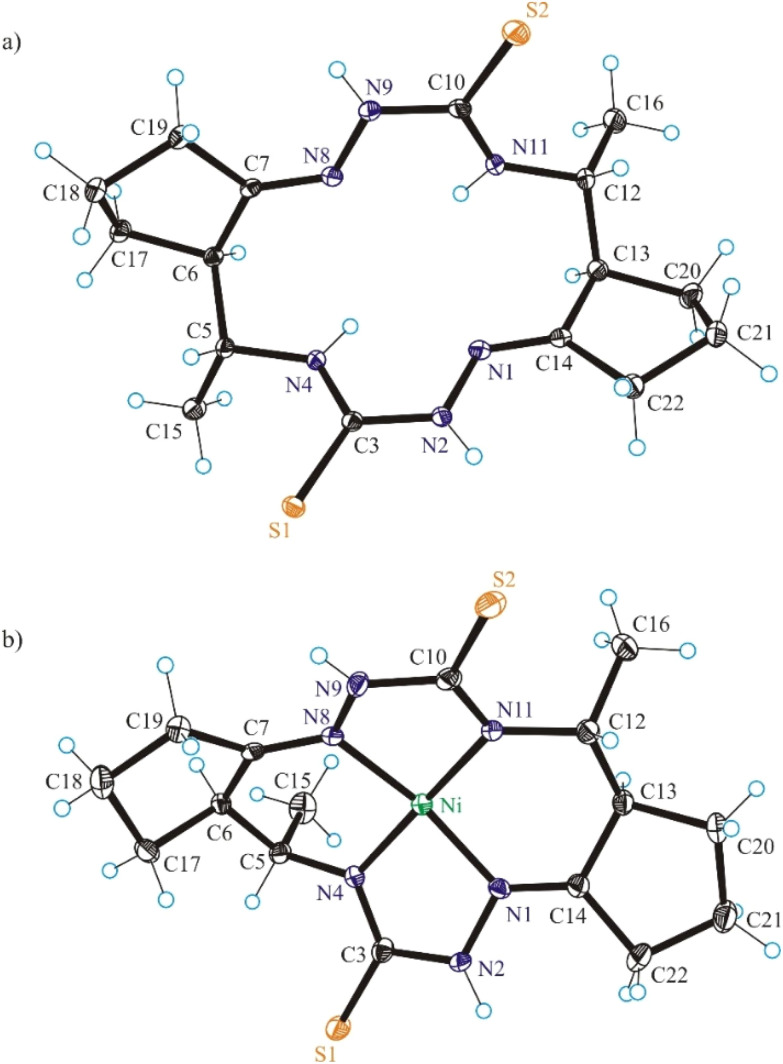
ORTEP views of (a) H_2_L^S^ and (b) Ni^II^L^S^ as diastereomers with 5*R*,6*R*,12*R*,13*R*-configuration. The thermal ellipsoids are drawn at 50% probability level. Selected bond distances (Å) and bond angles (°) in H_2_L^S^: N1–N2 1.3853(11), N2–C3 1.3662(11), C3–S1 1.6943(9), C3–N4 1.3316(12), N4–C5 1.4673(11), C5–C6 1.5491(13), C6–C7 1.5183(13), C7–N8 1.2806(11), N8–N9 1.3818(12), N9–C10 1.3723(12), C10–S2 1.6853(10), C10–N11 1.3294(13), N11–C12 1.4599(11), C12–C13 1.5439(15), C13–C14 1.5199(13), C14–N1 1.2780(12); *Θ*_N1–N2–C3–N4_ = –0.62(13), *Θ*_N8–N9–C10–N11_ = 8.24(12); in Ni^II^L^S^: Ni–N1 1.854(3), Ni–N4 1.863(3), Ni–N8 1.871(2), Ni–N11 1.873(3), N1–N2 1.394(4), N2–C3 1.393(4), C3–S1 1.695(3), C3–N4 1.316(4), N4–C5 1.469(4), C5–C6 1.527(4), C6–C7 1.508(4), C7–N8 1.282(4), N8–N9 1.397(3), N9–C10 1.381(4), C10–S2 1.707(3), C10–N11 1.312(4), N11–C12 1.477(4), C12–C13 1.529(4), C13–C14 1.509(4), C14–N1 1.279(4); *Θ*_N1–N2–C3–N4_ = 12.5(4), *Θ*_N8–N9–C10–N11_ = 6.4(4).

**Fig. 2:**
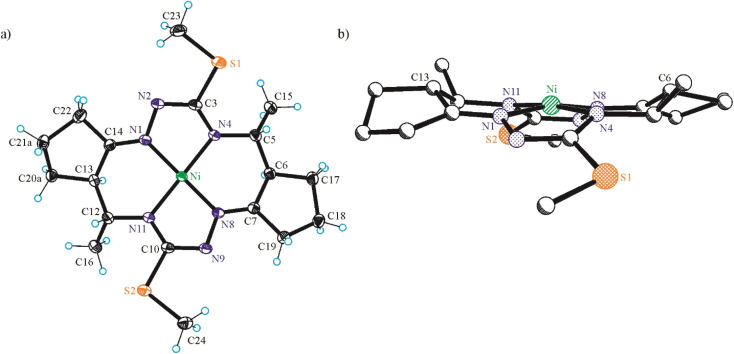
ORTEP view of (a) Ni^II^L^SMe^ as diastereomer with 5*R*,6*R*,12*R*,13*R*-configuration and the thermal ellipsoids drawn at 50% probability level, and (b) ball-and-stick projection of the same molecule perpendicular to the NiN_4_ mean plane. Selected bond distances (Å) and torsion angles (°): Ni–N1 1.8471(11), Ni–N4 1.8845(10), Ni–N8 1.8486(11), Ni–N11 1.8868(10), N1–N2 1.4008(14), N2–C3 1.3239(17), C3–S1 1.7717(12), C3–N4 1.3447(16), N4–C5 1.4703(16), C5–C6 1.5370(17), C6–C7 1.5030(17), C7–N8 1.2854(16), N8–N9 1.3987(14), N9–C10 1.3242(17), C10–S2 1.7754(13), C10–N11 1.3370(16), N11–C12 1.4702(16), C12–C13 1.5295(19), C13–C14 1.5037(18), C14–N1 1.2839(16); *Θ*_N1–N2–C3–N4_ = 5.75(16), *Θ*_N8–N9–C10–N11_ = 2.55(16).

**Fig. 3:**
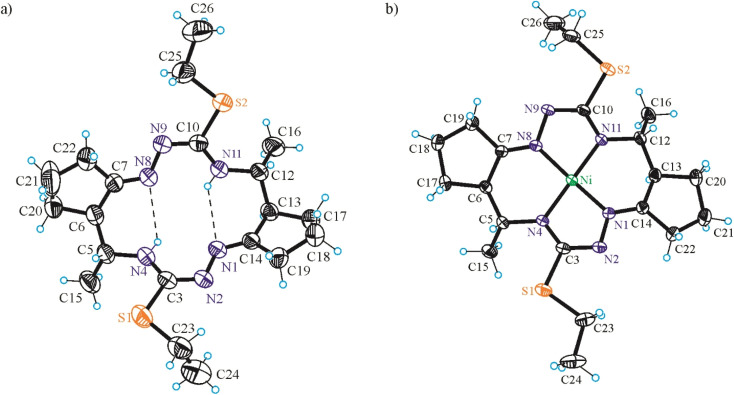
ORTEP view of (a) H_2_L^SEt^ and (b) Ni^II^L^SEt^ as diastereomers with 5*R*,6*R*,12*R*,13*R*-configuration. The thermal ellipsoids are drawn at 50% probability level. Selected bond distances (Å) and bond angles (°) in H_2_L^SEt^: N1–N2 1.413(2), N2–C3 1.293(3), C3–S1 1.777(2), C3–N4 1.343(3), N4–C5 1.463(3), C5–C6 1.539(3), C6–C7 1.516(3), C7–N8 1.276(3), N8–N9 1.411(2), N9–C10 1.297(3), C10–S2 1.764(2), C10–N11 1.349(3), N11–C12 1.455(3), C12–C13 1.544(3), C13–C14 1.520(3), C14–N1 1.274(3); *Θ*_N1–N2–C3–N4_ = −1.1(3), *Θ*_N8–N9–C10–N11_ = −0.8(3); in Ni^II^L^SEt^: Ni–N1 1.8476(15), Ni–N4 1.8729(14), Ni–N8 1.8535(15), Ni–N11 1.8767(14), N1–N2 1.396(2), N2–C3 1.322(2), C3–S1 1.7717(18), C3–N4 1.340(2), N4–C5 1.468(2), C5–C6 1.534(2), C6–C7 1.503(2), C7–N8 1.282(2), N8–N9 1.4028(19), N9–C10 1.322(2), C10–S2 1.7709(18), C10–N11 1.339(2), N11–C12 1.470(2), C12–C13 1.536(3), C13–C14 1.503(3), C14–N1 1.282(2); *Θ*_N1–N2–C3–N4_ = 1.6(2), *Θ*_N8–N9–C10–N11_ = 4.1(2).

The results of SC-XRD analyses of H_2_L^S^ and NiL^S^ are shown in Fig. 1, those for complex NiL^SMe^ in Fig. 2, while the structures of H_2_L^SEt^ and NiL^SEt^ in Fig. 3. Selected bond distances and bond angles are listed in the captions to figures. Both the metal-free macrocycles and nickel(ii) complexes crystallized as orthorhombic, triclinic or monoclinic crystals in achiral space groups as racemic mixtures of 5*R*,6*R*,12*R*,13*R* and 5*S*,6*S*,12*S*,13*S* enantiomers (Table S3 in the ESI†).

**Chart 4:**
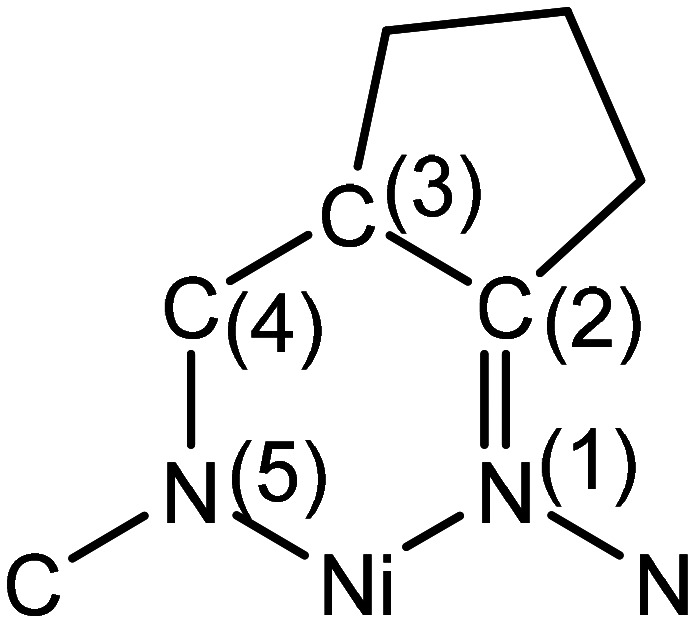
Chelate rings present in Ni^II^L^S^.

The conformation of the macrocycle in Ni^II^L^S^ is better described in terms of the component chelate rings formed upon binding to central atom. While the two 5-membered rings are quite similar and adopt envelope conformations (see Table S1†), the two six-membered rings shown in Chart 4 adopt two different conformations A and B according to classification proposed by Curtis^40^ for macrocyclic compounds (see Table S2 in the ESI†), which have two secondary amine and two imine donor atoms. The two conformations, A and B, can be distinguished in the complex by atom displacements from the MN_2_ plane (Tables S1and S2 in the ESI,†Chart 4), atom C(4) being displaced further on the same side of this plane than C(3) for A, and less than for B. So, the 6-membered chelate ring NiN4C5C6C7N8 adopted conformation A, while NiN11C12C13C14N1 the conformation B.

The free macrocycle H_2_L^SEt^ and coordinated macrocycic ligands in Ni^II^L^SMe^ and Ni^II^L^SEt^ (Fig. 2 and 3) have a saddle-shaped distortion from planarity, *i.e.*, with five-membered chelate rings NiNCNN tilted to one side of the molecular plane, and the six-membered rings tilted to the other side. A consequence of the saddle shape of the metal-free macrocycles is that lone pairs of the four cavity N atoms are directed out of the N_4_ plane, while in the nickel(ii) complexes the lone pairs lie in the NiN_4_ plane. Generally, the macrocycles in complexes Ni^II^L^SMe^ and Ni^II^L^SMe^ are more “symmetric” than in Ni^II^L^S^.

Square-planar coordination geometry is observed in all three complexes studied by SC-XRD. Comparison of the bond lengths around low-spin Ni(ii) in Ni^II^L^S^, Ni^II^L^SMe^ and Ni^II^L^SEt^ shows that Ni–N_hydrazine_ bonds are commonly shorter than Ni–N_thioamide_ bonds, as also observed in the most part of square-planar nickel(ii) complexes based on isothiosemicarbazide.^13^

#### Cyclic voltammetry

**Fig. 4:**
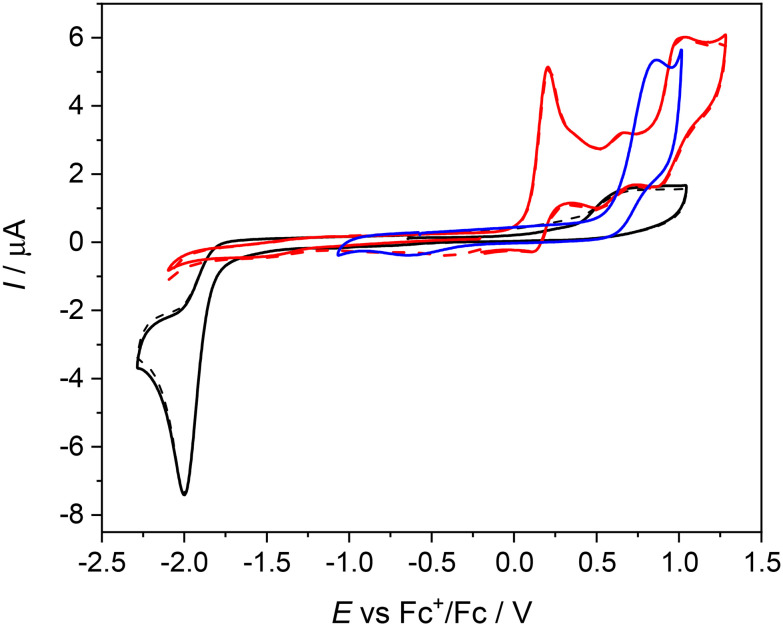
Cyclic voltammograms of Ni^II^L^S^ (black traces, solid trace – the first scan, dashed trace – the second scan), Ni^II^L^SMe^ (red traces), and of H_2_L^S^ (blue trace) in 0.2 M *n*Bu_4_NPF_6_/CH_2_Cl_2_ both in anodic and cathodic parts (Pt-disc working electrode, scan rate 100 mV s^−1^).

**Fig. 5:**
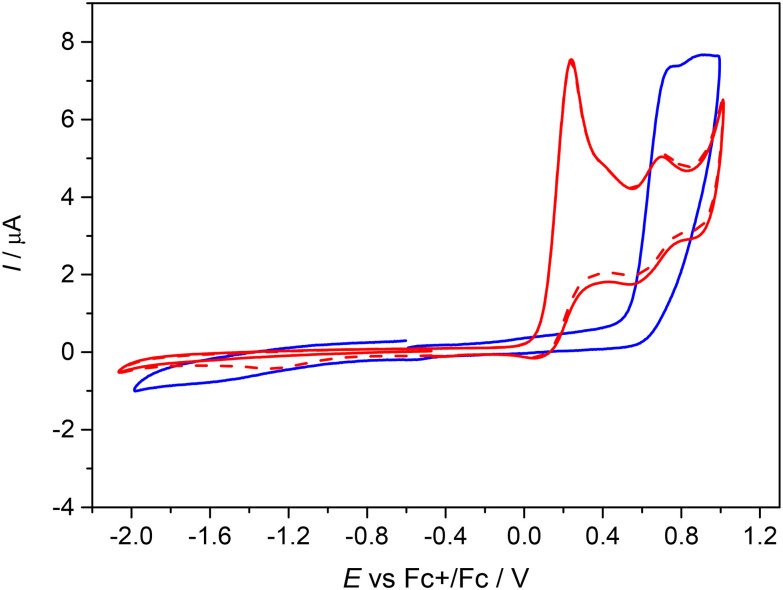
Cyclic voltammograms of Ni^II^L^SEt^ (red traces, solid trace – the first scan, dashed trace – the second scan), and of H_2_L^SEt^ (blue trace) in 0.2 M *n*Bu_4_NPF_6_/CH_2_Cl_2_ both in anodic and cathodic parts (Pt-disc working electrode, scan rate 100 mV s^−1^).

The redox properties of the investigated organic compounds and nickel(ii) complexes have been studied by cyclic voltammetry (CV), at a Pt working electrode in 0.2 M *n*Bu_4_NPF_6_/CH_2_Cl_2_ solutions, at room temperature. Notably, the investigated complexes Ni^II^L^SMe^, Ni^II^L^SEt^, and the corresponding metal-free macrocyclic compounds are not redox-active in the cathodic region (see cathodic part in Fig. 4 and 5). No reduction was observed for them in the potential window available except of complex Ni^II^L^S^, where fully irreversible reduction peak was found at highly negative cathodic potential *E*_pc_ = −2.00 V (*vs*. Fc^+^/Fc at a scan rate of 100 mV s^−1^). For this complex only minor oxidation peaks were observed in the anodic part indicating the presence of either free ligand or minor impurity in the sample. In this anodic potential region the free ligand is redox active with irreversible oxidation peak at *E*_pa_ = +0.86 V *vs.* Fc^+^/Fc (see blue trace in Fig. 4). For complex Ni^II^L^SMe^ two main oxidation events were identified with *E*^1^_pa_ = +0.21 V and *E*^2^_pa_ = +1.01 V *vs*. Fc^+^/Fc (*E*^1^_pa_ = +0.81 V and *E*^2^_pa_ = +1.61 V *vs*. NHE).

The first oxidation peak is nearly chemically irreversible (but with a hint of small counter peak observed in a back scan) and following this peak a new minor reversible redox couple appears upon oxidation at *E*′_pa_ = +0.66 V *vs*. Fc^+^/Fc. As the sample was rigorously purified and analyzed before voltammetric measurements we attribute this new minor peak to the redox active follow up product formed in the region of the first nearly irreversible oxidation. The second main oxidation peak at +1.01 V shows much higher degree of chemical reversibility. Very similar redox behavior was found for Ni^II^L^SEt^ (see red traces in Fig. 5) with *E*^1^_pa_ = +0.24 V and *E*′_pa_ = +0.70 V *vs*. Fc^+^/Fc. Again, a minor anodic peak at around 0.7 V indicates the follow up product after the first oxidation step in solution leading to the formation of new redox active species. The first oxidation potential of Ni^II^L^SEt^ is substantially negatively shifted in comparison to the corresponding metal-free proligand H_2_L^SEt^ with *E*^1^_pa_ = +0.75 V *vs*. Fc^+^/Fc indicating a strong influence of the central atom. Consequently, we can assume that the one-electron oxidized complex represents a system with a substantially noninnocent character of the ligand.

#### Oxygen evolution reaction study

**Fig. 6:**
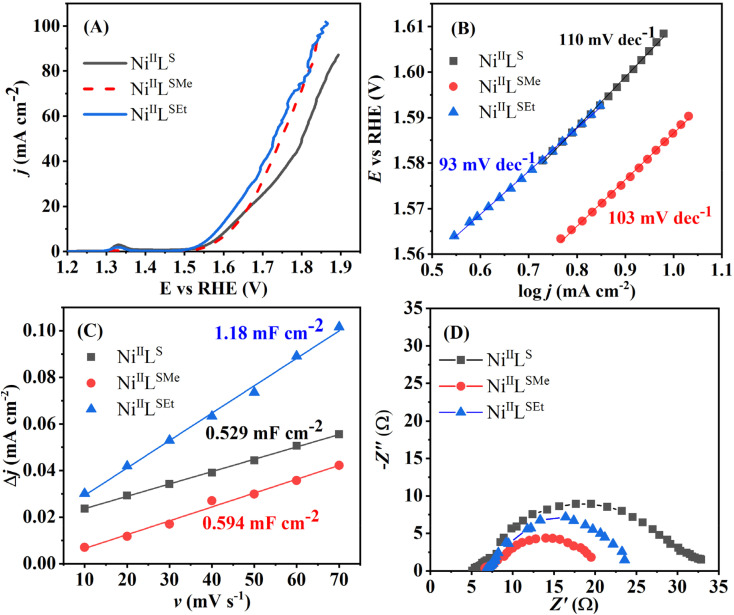
LSVs of Ni^II^L^SEt^, Ni^II^L^SMe^ and Ni^II^L^S^ studied in a form of a thin film on a glassy carbon support at 10 mV s^−1^ (A) with the corresponding Tafel plots (B), double-layer capacitance determination plots (Δ*j vs. v*) (C) and Nyquits plots at 1.67 V *vs*. RHE (D). All measurements were done in 1 M KOH.

The performance of the three Ni(ii) complexes under OER conditions was evaluated from the linear sweep voltammetry (LSV) measurements in 1 M KOH, Fig. 6A. The voltammograms of Ni^II^L^SEt^ and Ni^II^L^S^ show a low-intensity peak at *ca.* 1.33 V that can be conceivably attributed to the Ni^2+^/Ni^3+^ oxidation. This increment of Ni oxidation state typically favors the adsorption/desorption of the intermediates formed during the OER (see below).^41^ All three Ni(ii) complexes delivered high OER performance. It was previously reported that Ni(ii) in the square-planar configuration endows electrocatalysts with high intrinsic OER activity.^41^ The reaction was observed to start the earliest, *i.e.*, at the lowest onset potential in the case of Ni^II^L^SEt^. Accordingly, the overpotential to reach 10 mA cm^−2^ (*η*_10_) was *ca.* 20 and 30 mV lower in case of Ni^II^L^SEt^ (350 mV) compared to Ni^II^L^S^ (370 mV) and Ni^II^L^SMe^ (380 mV), respectively. Overpotential is calculated as the difference between the measured potential and the theoretical equilibrium potential value of oxygen electrode. The difference in the performance of the three complexes becomes even more pronounced at higher current densities and, *e.g.*, the overpotential to reach 50 mA cm^−2^ (*η*_50_) was 80 and 20 mV lower in case of Ni^II^L^SEt^ (480 mV) compared to Ni^II^L^S^ (560 mV) and Ni^II^L^SMe^ (500 mV), respectively. Moreover, Ni^II^L^SEt^ greatly outperformed commercial IrO_2_ (one of the currently suggested benchmark electrocatalysts for OER) (*η*_10_ = 620 mV) studied under the same conditions^32^ with 270 mV lower *η*_10_ in case of Ni^II^L^SEt^ (IrO_2_ did not reach 50 mA cm^−2^ in the given potential range). Comparison with other transition metal-based electrocatalysts reported in literature revealed comparable or higher performance of the herein studied Ni(ii) complexes. For instance, cobalt–benzimidazole swapped metal–organic macrocycle with reduced graphene oxide coated with Ni foam showed overpotential of 350 mV at 10 mA cm^−2^.^42^ Similarly, ultrathin CoMn layered double hydroxide exhibited similar value of overpotential of 350 mV at 10 mA cm^−2^ in 1 M KOH.^43^

The current density at the overpotential of 400 mV was the highest one in the case of Ni^II^L^SEt^ (22.4 mA cm^−2^), followed by Ni^II^L^S^ (15.2 mA cm^−2^) and then Ni^II^L^SMe^ (13.9 mA cm^−2^). IrO_2_ delivered *ca.* 35 times lower current density than Ni^II^L^SEt^ at the same overpotential value.^33^

Tafel analysis of the intrinsic OER activity of the complexes was further performed. Tafel slope, reflecting the rate of increase of current density with potential, was found to be the lowest in the case of OER at Ni^II^L^SEt^ (93 mV dec^−1^), followed by Ni^II^L^SMe^ (103 mV dec^−1^) and then Ni^II^L^S^ (110 mV dec^−1^) (Fig. 6B). The value determined for Ni^II^L^SEt^ was comparable to that of IrO_2_ (96 mV dec^−1^).^32^

**Fig. 7:**
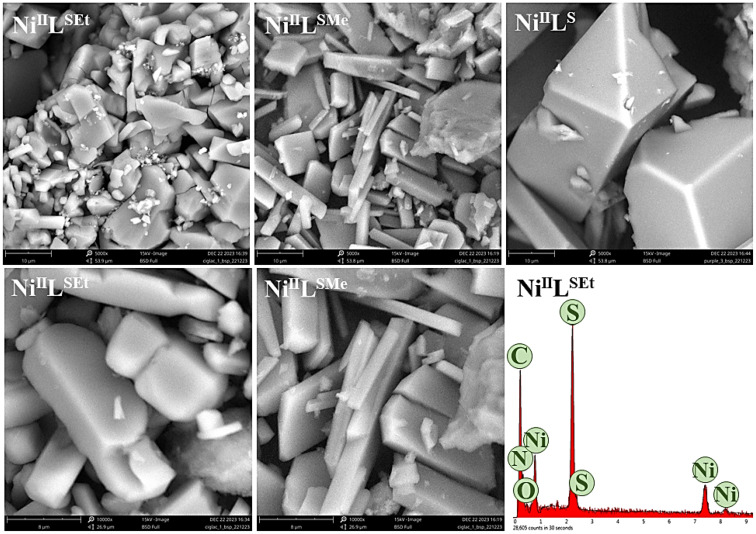
SEM images of studies Ni(ii)-complexes at magnifications of 5000× (upper row) and 10 000× (lower row) with EDX spectra of Ni^II^L^SEt^.

Scanning electron microscopy (SEM) studies of the as-prepared complexes illustrate different morphologies (Fig. 7). Ni^II^L^SEt^ is composed of cuboids and rod-like particles, while Ni^II^L^S^ displays a rod-like morphology as well. Particles of Ni^II^L^SMe^ were observed to be notably larger than in the case of the two other complexes. Energy-dispersive X-ray spectroscopy (EDX) analysis of the three Ni(ii)-complexes confirmed the presence of Ni, C, N, S and O elements.

Double-layer capacitance (*C*_dl_) of Ni^II^L^SEt^ (1.18 mF cm^−2^) was determined to be *ca.* double that of Ni^II^L^SMe^ (0.594 mF cm^−2^) and Ni^II^L^S^ (0.529 mF cm^−2^) (Fig. 6C). This suggests that Ni^II^L^SEt^ possess large surface area, offering abundant active sites for surface catalytic reactions and thus contributing to its high activity towards OER. Electrochemical active surface area (ECSA) is then calculated by dividing the *C*_dl_ value with a capacitance of 40 μF previously reported for Ni-based OER catalysts.^44^ The highest ECSA of 29.5 cm^2^ was evaluated for Ni^II^L^SEt^, well above those displayed by Ni^II^L^SMe^ (14.85 cm^2^) and Ni^II^L^S^ (13.22 cm^2^). Corresponding roughness factors were determined to be 118 for Ni^II^L^SEt^, 59.4 for Ni^II^L^SMe^ and 52.8 for Ni^II^L^S^.

Nyquist plots (Fig. 6D) reveal similar values of solution resistance (*R*_s_) of 5–7 Ω suggesting the comparable surface porosity of the three materials. On the other hand, the charge-transfer resistance (*R*_ct_) of Ni^II^L^SEt^ (16.8 Ω) was comparable to that of Ni^II^L^SMe^ (15.3 Ω), but almost twice lower than that of Ni^II^L^S^ (29.9 Ω). This further suggests the fastest electron-transfer capability of Ni^II^L^SEt^ and Ni^II^L^SMe^ and accounts for their higher current densities reached.

**Fig. 8:**
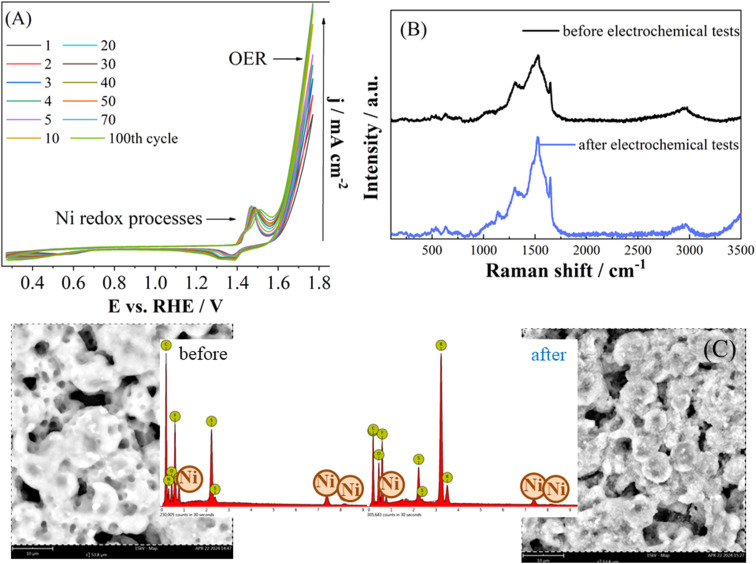
CVs of Ni^II^L^SEt^ during continuous cycling (A, 1^st^–100^th^ cycle) along with the Raman spectra (B) and SEM images with EDX spectra (C) of Ni^II^L^SEt^ working electrode before and after chronoamperometric test at 1.67 V.

As stability of the electrocatalysts is crucial for practical applications, continuous cycling was conducted. Current density was observed to continuously increase within the first 40 cycles (Fig. 8A) slightly decreasing with further cycling. The observed increase of current density suggests increment of Ni oxidation state during the stability test^45^ with Ni oxidation peak shifting somewhat positively from 1.47 V to 1.50 V. This further confirms that the Ni represents the active sites for OER. Namely, different reconstruction processes might occur during cycling under OER conditions involving formation of Ni oxidized species.^41,46^ Reversible reconstruction processes involve potential-dependent phase changes, the dissolution/redeposition of surface Ni atoms, and the adsorption/desorption of OER intermediates.^47^ Redox transformations of catalytic centers and the generation/refilling of vacancies proceed in parallel. Irreversible transformations may include phase changes and morphological or structural alterations.

Namely, nickel oxide (NiO) or α-phase of nickel hydroxide (α-Ni(OH)_2_) are formed in alkaline media.^47,48^ Generated α-Ni(OH)_2_ comprises layers with H_2_O molecules or OH^−^ ions in the interlayer space. It typically converts to β-Ni(OH)_2_ phase as a result of its dissolution and recrystallization in aqueous media. Generated β-Ni(OH)_2_ is the closest-packed, hexagonal structure of Ni^2+^ and OH^−^ ions, with no ions in the interlayer space. Under OER conditions, *i.e.*, at more positive potentials, this phase usually reversibly transforms to nickel oxyhydroxide (β-NiOOH) with Ni in the oxidation state of +3. Moreover, a few γ-Ni(OH)_2_ phases comprising much larger interlayer distances, can also be present. Under OER conditions, α-Ni(OH)_2_ can reversibly transforms to γ-NiOOH. In addition, β-NiOOH can be irreversibly overcharged in highly alkaline media forming the γ-NiOOH. The presence of Ni^2+^ (hydroxide species) along with Ni^3+^ species has been reported to be fundamental for active-site formation in OER catalysis by Ni-based materials.^49,50^

Ni^II^L^SEt^ working electrode was additionally analyzed before and after the electrochemical tests by using SEM-EDX and Raman spectroscopy, Fig. 8B and C. Minor changes in the electrode surface and elements composition could be observed. As mentioned, in case of heterogeneous complexes as catalysts such as those studied herein, leaching of metal ions and their subsequent redeposition might occur and cause a reactivation effect.^51^ On the other hand, in case of homogeneous complexes as catalysts for water oxidation (*i.e.*, catalysts present in the aqueous phase), their degradation and formation of transition metal oxides/oxyhydroxides on the electrode surface have been reported.^52,53^

The herein synthesized material is assumed to undergo dynamic reconstruction under oxidative OER conditions, forming an active oxyhydroxide layer. These phase transformations, which are dependent on the applied potential, further lead to structural changes with the formation of a new surface layer, changes in the oxidation state of interfacial Ni cations, and number of lattice oxygen vacancies. These open the possibility of OER proceeding by both conventional adsorbate evolution mechanism (involving oxygen-containing intermediates from the electrolyte) and the lattice-oxygen mediated mechanism (involving lattice oxygen vacancies) with the latter being associated with increased OER activity of Ni-based materials.^47^

## Conclusion

Successful synthesis of new 14-membered bis-thiosemicarbazide and bis-isothiosemicarbazide macrocycles and their nickel(ii) complexes is reported and the results are supported by spectroscopic data (^1^H and ^13^C NMR, IR, UV–vis), ESI-MS and SC-XRD analysis. Moreover, potential application of the prepared complexes for electrocatalysis of OER in alkaline media was scrutinized for the first time. Ni^II^L^SEt^ delivered a current density of 10 mA cm^−2^ and 50 mA cm^−2^ at low overpotential of 350 mV and 480 mV in KOH electrolyte of moderate concentration (1 M), with Tafel slope of 93 mV dec^−1^. In addition, Ni^II^L^SEt^ exhibited high stability during long-term operation. Considering the exhibited high performance for OER along with the synthesis directly scalable to industrial level and the use of only non-precious metals, the herein prepared Ni complexes are suggested as promising candidates for alkaline water electrolysis.

## Experimental

### Chemicals

Triethylamine, NiCl_2_·6H_2_O, NaH, NH_2_OH·HCl, TsOH·H_2_O, iodomethane, iodoethane, Ni(OAc)_2_·4H_2_O were purchased from commercial suppliers and used without further purification. All solvents and liquid reagents purchased from commercial sources and used for the synthesis of metal-free macrocycles were distilled prior use. Petroleum ether had a distillation window 40–70 °C. Anhydrous MeCN was obtained according to the standard procedure. Pyrimidine 3 was prepared according to the reported procedure.^36^

#### (7*E*,14*E*)-(5R*,6R*,12R*,13R*)-5,12-Dimethyl-6,7:13,14-bis(trimethylene)-1,2,4,8,9,11-hexaazacyclotetradeca-7,14-diene-3,10-dithione (6, H_2_L^S^) [(7*E*,14*E*)-(5R*,6R*,12R*,13R*)-6]

*Method A* (Table 1, entry 3): A 10 mL round-bottom flask equipped with a magnetic stirring bar was successively charged with a 97 : 3 mixture of compounds 3 and 2 (0.180 g, 0.90 mmol),^36^ NH_2_OH·HCl (0.078 g, 1.12 mmol), and EtOH (3 mL) at room temperature. The resulting mixture was refluxed under stirring on a hot plate magnetic stirrer for 2 h. At the beginning of heating, a solution quickly forms, from which after 3 min a white solid begins to precipitate. After completion of the reaction, the solvent was removed under reduced pressure, the solid residue was triturated with H_2_O until suspension formed, and cooled to 0 °C. The precipitate was filtered, thoroughly washed with ice-cold water, petroleum ether, and dried to give compound 6 (0.150 g, 92%; white solid) as a mixture of (7*E*,14*E*)-(5R*,6R*,12R*,13R*)-diastereomer (≥90%) with small amounts of some other diastereomers. Compound 6 with similar purity was also prepared by method A using multi-gram loadings. For example, the treatment of a 97 : 3 mixture of compounds 3 and 2 (8.213 g, 40.80 mmol) with NH_2_OH·HCl (3.555 g, 51.16 mmol) in refluxing EtOH (120 mL) for 2 h gave 6 (7.226 g, 97%) as a white solid. An analytically pure sample of (7*E*,14*E*)-(5R*,6R*,12R*,13R*)-6 (2.09 g; white solid) was obtained after crystallization of crude 6 (2.95 g) from butan-1-ol (140 mL). Anal. Calcd for C_16_H_26_N_6_S_2_, %: C, 52.43; H, 7.15; N, 22.93. Found, %: C, 52.34; H, 7.38; N, 23.13. Mp 261 °C (decomp., black foam; butan-1-ol) (heating rate close to the melting point is about 1 °C per 15–20 s; at a lower heating rate, only decomposition occurs without melting). IR (KBr) *ν*, cm^−1^: 3287 (br vs), 3208 (br s) (*ν* NH), 1653 (m) (*ν* C=N), 1542 (br vs), 1519 (s), 1491 (s), 1478 (s) (thioamide-II), 1215 (s), 1183 (s), 1138 (s); ^1^H NMR spectrum (600.13 MHz, DMSO-*d*_6_) *δ*: 10.08 (2 × 1H, s, 2 NH-N), 8.72 (2 × 1H, d, ^3^*J* = 5.8 Hz, 2 N*H*-CH), 3.87 (2 × 1H, ddq, ^3^*J* = 11.0, ^3^*J* = 6.1, ^3^*J* = 5.8 Hz, H-5 and H-12), 2.64 (2 × 1H, dddd, ^3^*J* = 11.0, ^3^*J* = 8.0, ^3^*J* = 4.4, ^4^*J* = 1.6 Hz, H-6 and H-13), 2.40–2.46 (2 × 1H, m, H_A_ in 2 CH_2_C=N), 2.31–2.37 (2 × 1H, m, H_B_ in 2 CH_2_C=N), 1.93–1.99 (2 × 1H, m, H_A_ in 2 C*H*_2_CH_2_CH_2_C=N), 1.69–1.81 (2 × 2H, m, 2 CH_2_C*H*_2_CH_2_C=N), 1.63–1.68 (2 × 1H, m, H_B_ in 2 C*H*_2_CH_2_CH_2_C=N), 1.33 (2 × 3H, d, ^3^*J* = 6.1 Hz, 2 CH_3_); ^13^C NMR spectrum (150.90 MHz, DMSO-*d*_6_) *δ*: 176.57 (C-3, C-10), 160.07 (C-7, C-14), 52.12 (C-5, C-12), 47.73 (C-6, C-13), 28.94 (2 CH_2_CH_2_*C*H_2_C=N), 28.76 (2 *C*H_2_CH_2_CH_2_C=N), 22.77 (2 CH_2_*C*H_2_CH_2_C=N), 20.31 (2 CH_3_); EIMS spectrum: *m*/*z* 368 [3, (M + 2)^+^], 367 [6, (M + 1)^+^], 366 (33, M^+^), 291 (4), 250 (3), 210 (14), 184 (37), 183 (100), 182 (19), 169 (13), 168 (38), 167 (42), 154 (8), 150 (24), 142 (10), 141 (11), 140 (14), 128 (10), 125 (19), 124 (26), 123 (50), 115 (40), 110 (42), 109 (37), 108 (41), 106 (24), 97 (55), 86 (52), 82 (66), 81 (57), 80 (64), 69 (58), 67 (83), 60 (83), 55 (62), 53 (54), 44 (80), 41 (90). HRMS (ESI-TOF) *m*/*z* calcd for C_16_H_27_N_6_S_2_ [M + H]^+^ 367.1733, found 367.1729.

*Method B* (Table 1, entry 1): A solution of a 97 : 3 mixture of compounds 3 and 2 (3.192 g, 15.86 mmol) and N_2_H_4_·H_2_O (7.944 g, 158.69 mmol) in EtOH (50 mL) was stirred under reflux for 1 h, then the solvent was removed under reduced pressure at 60 °C. The oily residue was co-evaporated with toluene (3 × 25 mL), dissolved in CHCl_3_ (60 mL), and washed with brine (3 × 5 mL). The solvent was removed under reduced pressure, the residual foam was dried in a vacuum desiccator over P_2_O_5_ overnight. The obtained crude hydrazone 4 (3.592 g, 16.68 mmol) was dissolved in EtOH (35 mL) upon warming, then solid TsOH·H_2_O (3.499 g, 18.39 mmol) and EtOH (15 mL) were added, and the mixture was stirred under reflux for 2 h. The resulting suspension was evaporated to dryness under reduced pressure, the solid residue was triturated with saturated aqueous solution of NaHCO_3_ until suspension formed, and cooled. The precipitate was filtered, thoroughly washed with ice-cold H_2_O, petroleum ether, and dried to give compound 6 (2.530 g, 83%) as a mixture of (5R*,6R*,12R*,13R*)-diastereomer (≥94%) with small amounts of some other diastereomers.

*Method C* (Table 1, entry 2): To a stirred solution of NH_2_OH·HCl (0.115 g, 1.65 mmol) and NaOAc·3H_2_O (0.228 g, 1.68 mmol) in H_2_O (1 mL) was added a 97 : 3 mixture of compounds 3 and 2 (0.268 g, 1.33 mmol), followed by EtOH (4 mL), and the reaction mixture was stirred at room temperature. Approximately 3 h after the beginning of the reaction, the solid was completely dissolved. After 4.5 h, the solution was evaporated to dryness under reduced pressure, the residue was dissolved in CHCl_3_ (15 mL), and washed with Н_2_О (3 × 5 mL), brine (2 × 5 mL). The solvent was removed under reduced pressure, the residual foam was dried in a vacuum desiccator over P_2_O_5_ overnight. To the obtained crude oxime 5 (0.160 g, 0.74 mmol) were added TsOH·H_2_O (0.157 g, 0.83 mmol) and EtOH (4 mL), and the mixture was stirred under reflux for 2 h. At the beginning of heating, a solution quickly forms, from which after 11 min a white solid begins to precipitate. After completion of the reaction, the solvent was removed under reduced pressure, the solid residue was triturated with saturated aqueous solution of NaHCO_3_ until suspension formed, and cooled to 0 °C. The precipitate was filtered, thoroughly washed with ice-cold water, petroleum ether, and dried to give compound 6 (0.087 g, 64%) as a mixture of (7*E*,14*E*)-(5R*,6R*,12R*,13R*)-diastereomer (≥90%) with small amounts of some other diastereomers.

#### (7*E*,14*E*)-(5R*,6R*,12R*,13R*)-3,10-Di(ethylthio)-5,12-dimethyl-6,7:13,14-bis(trimethylene)-1,2,4,8,9,11-hexaazacyclotetradeca-2,7,9,14-tetraene (8, H_2_L^SEt^) [(7*E*,14*E*)-(5R*,6R*,12R*,13R*)-8]

To NaH (0.163 g, 6.77 mmol) and crude macrocycle 6 (1.129 g, 3.08 mmol) was added dry MeCN (22 mL), and the obtained mixture was stirred at room temperature for 10 min, then iodoethane (3.162 g, 20.27 mmol) was added. The resulting mixture was stirred at room temperature for 5 h. During about 2 h from the beginning of the reaction slow gas evolution was observed, and the initial suspension turned into an almost clear creamy solution with small amount of grey precipitate. After reaction was completed the solvent was removed under reduced pressure, the solid residue was triturated with saturated aqueous solution of NaHCO_3_ until suspension formed, and cooled. The precipitate was filtered, thoroughly washed with ice-cold H_2_O, and dried to give compound 8 (1.280 g, 98%; very light creamy solid) as a diastereomeric mixture with a strong predominance of (7*E*,14*E*)-(5R*,6R*,12R*,13R*)-diastereomer (≥90%). An analytically pure sample of (7*E*,14*E*)-(5R*,6R*,12R*,13R*)-diastereomer of 8 (0.862 g, white needles) was obtained after crystallization of crude 8 (1.269 g) from EtOH (13 mL). Anal. Calcd for C_20_H_34_N_6_S_2_ (*M*_r_ = 422.66), %: C, 56.84; H, 8.11; N, 19.88. Found, %: C, 56.87; H, 8.15; N, 19.89. Mp 139–140 °C (EtOH). IR (KBr) *ν*, cm^−1^: 280 (br s) (*ν* NH), 1630 (s) (*ν* C=N), 1562 (vs) (N=C-NH), 1306 (s), 1140 (m); ^1^H NMR spectrum (600.13 MHz, DMSO-*d*_6_) *δ*: 8.14 (2 × 1H, d, ^3^*J* = 3.4 Hz, 2 N*H*-CH), 3.22 (2 × 1H, ddq, ^3^*J* = 10.3, ^3^*J* = 6.0, ^3^*J* = 3.4 Hz, H-5 and H-12), 2.99 (2 × 1H, dq, ^2^*J* = 13.0, ^3^*J* = 7.3 Hz, H_A_ in 2 SCH_2_), 2.93 (2 × 1H, dq, ^2^*J* = 13.0, ^3^*J* = 7.3 Hz, H_B_ in 2 SCH_2_), 2.61–2.67 (2 × 1H, m, H_A_ in 2 CH_2_C=N), 2.45 (2 × 1H, dddd, ^3^*J* = 10.3, ^3^*J* = 8.1, ^3^*J* = 5.3, ^4^*J* = 1.7 Hz, H-6 and H-13), 2.31–2.37 (2 × 1H, m, H_B_ in 2 CH_2_C=N), 1.94–2.00 (2 × 1H, m, H_A_ in 2 C*H*_2_CH_2_CH_2_C=N), 1.69–1.76 (2 × 1H, m, H_A_ in 2 CH_2_C*H*_2_CH_2_C=N), 1.55–1.66 (2 × 2H, m, H_B_ in 2 C*H*_2_CH_2_CH_2_C=N and H_B_ in 2 CH_2_C*H*_2_CH_2_C=N), 1.29 (2 × 3H, d, ^3^*J* = 6.0 Hz, 2 CH_3_), 1.26 (2 × 3H, t, ^3^*J* = 7.3 Hz, CH_3_ in 2 SEt); ^13^C NMR spectrum (150.90 MHz, DMSO-*d*_6_) *δ*: 170.18 (C-7, C-14), 159.94 (C-3, C-10), 50.86 (C-5, C-12), 47.85 (C-6, C-13), 29.19 (2 CH_2_CH_2_*C*H_2_C=N), 28.90 (2 *C*H_2_CH_2_CH_2_C=N), 23.70 (2 SCH_2_), 22.71 (5-CH_3_, 12-CH_3_), 22.33 (2 CH_2_*C*H_2_CH_2_C=N), 14.42 (CH_3_ in 2 SEt).

### Synthesis of complexes

Ni^II^L^S^. To a suspension of H_2_L^S^ (110 mg, 0.3 mmol) in dry DMF (3 mL) under argon was added triethylamine (83.7 μL, 0.6 mmol). The solution was stirred at room temperature for 15 min, then nickel(ii) chloride hexahydrate (74.7 mg, 0.3 mmol) was added and the reaction mixture was heated at 90 °C for 1 h. Afterwards, the obtained solution was cooled down to room temperature. The red precipitate was filtered off, washed with cold methanol (5 × 1 mL) and dried *in vacuo*. Yield: 79 mg, 53%. Anal. Calcd for C_16_H_24_N_6_S_2_NiCl_2_ (*M*_r_ = 423.22), %: C, 45.41; H, 5.72; N, 19.86; S, 15.15%. Found, %: C, 45.32; H, 5.72; N, 19.63; S, 15.13. ESI-MS in MeCN/MeOH +1% H_2_O (positive): *m*/*z* 423.16 [NiL^S^ + H]^+^, 445.11 [NiL^S^ + Na]^+^. ESI-MS in ACN/MeOH +1% H_2_O (negative): *m*/*z* 421.00 [NiL^S^-H]^−^. ^1^H NMR (700 MHz, DMSO-*d*_6_) *δ*, ppm: 10.50 (s, 2H, 2 and 9), 3.10–3.06 (m, 2H, 5 and 12), 2.73–2.68 (m, 4H, 6 and 13, (6- and 13-CH_2_-CH_2_-C*H*_2_)_*b*_), 2.45–2.39 (m, 2H, (6- and 13-CH_2_-CH_2_-C*H*_2_)_*a*_), 2.08–2.05 (m, 2H, (6- and 13-C*H*_2_-CH_2_-CH_2_)_*a*_), 1.89–1.85 (m, 2H, (6- and 13-CH_2_-C*H*_2_-CH_2_)_*a*_), 1.65–1.58 (m, 2H, (6- and 13-CH_2_-C*H*_2_-CH_2_)_*b*_), 1.50–1.44 (m, 2H, (6- and 13-C*H*_2_-CH_2_-CH_2_)_*b*_), 1.33 (d, 6H, 5- and 12-C*H*_3_). Due to the splitting of the signals of the methyl groups, those co-planar with CH 6 and 13 are indicated with an italic *a* as subindex. ^13^C NMR (151 MHz, DMSO-*d*_6_) *δ*, ppm: 181.62 (3 and 10), 165.82 (7 and 14), 55.44 (6 and 13), 51.63 (5 and 12), 31.18 (6- and 13-CH_2_-CH_2_-*C*H_2_), 30.53 (6- and 13-*C*H_2_-CH_2_-CH_2_), 23.23 (6- and 13-CH_2_-*C*H_2_-CH_2_), 21.16 (5- and 12-*C*H_3_). Single crystals suitable for X-ray diffraction study were obtained from mother liquor in a Schlenk tube upon standing at 4 °C overnight. UV–visible (MeOH), *λ*_max_, nm (*ε*, M^−1^ cm^−1^): 256sh; 271(16 764), 300sh, 356(38 256), 526(226). IR (ATR, selected bands, 
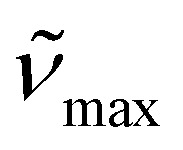
): 3319, 3070, 2876, 2646, 2322, 1660, 1546, 1446, 1362, 1277, 1199, 1084, 890, 647 cm^−1^.

Ni^II^L^SMe^. To a suspension of sodium hydride (14.4 mg, 0.6 mmol) in dry DMF (1.5 mL) under argon was added a suspension of H_2_L^S^ (110 mg, 0.3 mmol) in dry DMF (1.5 mL). The obtained solution was stirred at room temperature for 1 h, then iodomethane (37.35 μL, 0.6 mmol) was added and the solution was further stirred for 3 h. Then, nickel(ii) acetate tetrahydrate (24.88 mg, 0.1 mmol) was added and the solution was heated at 90 °C for 2 h. The reaction mixture was cooled down to room temperature. The red precipitate was filtered off, washed with methanol (5 × 1 mL) and dried *in vacuo*. Yield: 72 mg, 53%. Anal. Calcd for C_18_H_28_N_6_S_2_Ni (*M*_r_ = 451.28), %: C, 47.91; H, 6.25; N, 18.62; S, 14.21%. Found, %: C, 47.72; H, 6.22; N, 18.47; S, 14.09. ESI-MS in MeCN/MeOH +1% H_2_O (positive): *m*/*z* 451.21 [NiL^SMe^ + H]^+^. ESI-MS in ACN/MeOH +1% H_2_O (negative): *m*/*z* 449.11 [NiL^SMe^-H]^−^. ^1^H NMR (600 MHz, CDCl_3_) *δ* 2.99–2.82 (m, 4H, 5 and 12, (6- and 13-CH_2_-CH_2_-C*H*_2_)_*b*_), 2.58–2.51 (m, 2H, 6 and 13), 2.49 (s, 6H, 3- and 10-S-C*H*_3_), 2.44 (m, 2H, (6- and 13-CH_2_-CH_2_-C*H*_2_)_*a*_), 2.08 (dt, 2H, (6- and 13-C*H*_2_-CH_2_-CH_2_)_*a*_), 1.97–1.86 (m, 2H, (6- and 13-CH_2_-C*H*_2_-CH_2_)_*a*_), 1.67–1.56 (m, 2H, (6- and 13-CH_2_-C*H*_2_-CH_2_)_*b*_), 1.51–1.39 (m, 2H, (6- and 13-C*H*_2_-CH_2_-CH_2_)_*b*_), 1.32 (d, *J* = 5.8 Hz, 6H, 5- and 12-C*H*_3_). Due to the splitting of the signals of the methyl groups, those co-planar with CH 6 and 13 are indicated with an italic *a* as subindex. ^13^C NMR (151 MHz, CDCl_3_) *δ* 169.41 (3 and 10), 166.26 (7 and 14), 56.69 (6 and 13), 50.70 (5 and 12), 31.38 (6- and 13-*C*H_2_-CH_2_-CH_2_), 30.66 (6- and 13-CH_2_-CH_2_-*C*H_2_), 23.68 (6- and 13-CH_2_-*C*H_2_-CH_2_), 23.53 (5- and 12-*C*H_3_), 16.35 (3- and 10-S-*C*H_3_). Single crystals suitable for X-ray diffraction study were obtained by slow diffusion of diethyl ether into the mother liquor. UV–visible (MeOH), *λ*_max_, nm (*ε*, M^−1^ cm^−1^): 216(204 849), 236sh, 286(16 152), 318(6163), 400(4564), 530(222). IR (ATR, selected bands, 
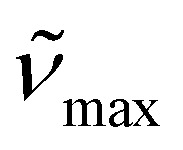
): 2955, 2925, 2867, 2831, 1463, 1286, 1142, 1089, 1012, 888, 828, 782, 653 cm^−1^.

Ni^II^L^SEt^. To a solution of H_2_L^SEt^ (63 mg, 0.15 mmol) in dry DMF (3 mL) under argon was added triethylamine (36.9 μL, 0.3 mmol). The solution was stirred at room temperature for 15 min, then nickel(ii) acetate tetrahydrate (37.3 mg, 0.15 mmol) was added and the reaction mixture was stirred at room temperature for 1 h. Afterwards, the volume of solution was reduced to 1/3 by concentration *in vacuo* and allowed to stand at 4 °C overnight. The red precipitate was filtered off, washed with cold methanol (3 × 2 mL) and dried *in vacuo*. Yield: 45 mg, 63%. Anal. Calcd for C_20_H_32_N_6_S_2_Ni (*M*_r_ = 479.33), %: C, 50.11; H, 6.73; N, 17.53; S, 13.38%. Found, %: C, 49.79; H, 6.68; N, 17.39; S, 13.33. ESI-MS in MeCN/MeOH +1% H_2_O (positive): *m*/*z* 479.20 [NiL^SEt^ + H]^+^. ^1^H NMR (600 MHz, CDCl_3_) *δ*, ppm: 3.14–3.01 (m, 4H, 3- and 10-S-C*H*_2_-CH_3_), 2.97–2.85 (m, 4H, 5 and 12, (6- and 13-CH_2_-CH_2_-C*H*_2_)_*b*_), 2.56–2.49 (m, 2H, 6 and 13), 2.43 (m, 2H, (6- and 13-CH_2_-CH_2_-C*H*_2_)_*a*_), 2.08 (dt, 2H, (6- and 13-C*H*_2_-CH_2_-CH_2_)_*a*_), 1.94–1.86 (m, 2H, (6- and 13-CH_2_-C*H*_2_-CH_2_)_*a*_), 1.64–1.57 (m, 2H, (6- and 13-CH_2_-C*H*_2_-CH_2_)_*b*_), 1.45 (ddd, 2H, (6- and 13-C*H*_2_-CH_2_-CH_2_)_*b*_), 1.32 (dd, 5- and 12-C*H*_3_, 3- and 10-S-CH_2_-C*H*_3_). Due to the splitting of the signals of the methyl groups, those co-planar with CH 6 and 13 are indicated with an italic *a* as subindex. ^13^C NMR (151 MHz, CHCl_3_) *δ*, ppm: 168.83(3 and 10), 165.93(7 and 14), 56.68(6 and 13), 50.71(5 and 12), 31.41(6- and 13-*C*H_2_-CH_2_-CH_2_), 30.63(6- and 13-CH_2_-CH_2_-*C*H_2_), 27.83(3- and 10-S-*C*H_2_-CH_3_), 23.76(5- and 12-*C*H_3_), 23.63(6- and 13-CH_2_-*C*H_2_-CH_2_), 15.00(3- and 10-S-CH_2_-*C*H_3_). Single crystals suitable for X-ray diffraction study were obtained from mother liquor in a Schlenk tube upon standing at 4 °C overnight. UV–visible (MeOH), *λ*_max_, nm (*ε*, M^−1^ cm^−1^): 216(23 232), 238sh, 289(18 880), 319(7249), 402(53 858), 529(301). IR (ATR, selected bands, 
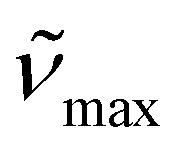
): 2957, 1450, 1370, 1333, 1297, 1245, 1142, 1093, 1006, 777 cm^−1^.

#### Physical measurements

Electrospray ionization mass spectrometry (ESI-MS) was carried out with Amazon speed ETD Bruker instrument. The sum formulae of the detected ions were determined using Bruker Compass DataAnalysis 5.3 based on the mass accuracy (Δ*m*/*z* ≤ 5 ppm) and isotopic pattern matching (SmartFormula algorithm). NMR spectra (solutions in DMSO-*d*_6_, CDCl_3_ at 25 °C) were acquired using a Bruker AV NEO 500 or Bruker Avance III 600 spectrometer at 600.13 (^1^H) and 150.90 (^13^C) MHz. ^1^H NMR chemical shifts are referenced to the residual proton signal in DMSO-*d*_6_ (2.50 ppm), CDCl_3_ (7.26 ppm). In ^13^C NMR spectra, central signal of DMSO-*d*_6_ (39.50 ppm)/CDCl_3_ (77.16 ppm) was used as a reference. Multiplicities are reported as singlet (s), doublet (d), triplet (t), quartet (q), and some combinations of these, multiplet (m). Selective ^1^H–^1^H decoupling, DEPT-135 experiments as well as HMQC, HMBC, and NOESY correlation techniques were used to aid in the assignment of ^1^H and ^13^C NMR signals. The atom numbering for the assignment of resonances for Ni(ii) complexes is given in Scheme S1.† Elemental analyses of ligands were performed using a Thermo Finnigan Flash EA1112 apparatus and elemental analysis of Ni(ii) complexes were carried out in a Carlo-Erba microanalyzer at the Microanalytical Laboratory of the University of Vienna. UV–vis spectra were measured on PerkinElmer UV–vis spectrophotometer Lambda 35 in the 240 to 700 nm window using samples dissolved in methanol. FTIR spectra of ligands were recorded using a Bruker Alpha-T spectrophotometer in KBr. IR spectra of complexes were recorded on a Bruker Vertex 70 Fourier transform IR spectrometer (4000–600 cm^−1^) using the ATR technique. SEM-EDX analysis was carried out using a Phenom™ ProX Desktop scanning electron microscope (ThermoFisher Scientific™, Waltham, MA, USA).

#### Crystallographic structure determination

X-ray diffraction measurements of H_2_L^S^, H_2_L^SEt^, NiL^S^, NiL^SMe^ and NiL^SEt^ were performed on Bruker X8 APEX-II CCD, Bruker D8 Venture and STOE diffractometers. Single crystals were positioned at 30, 40, 26, 27 and 40 mm from the detector, and 4868, 10 110, 1650, 2420 and 722 frames were measured, each for 30, 2, 3, 30 and 35 s over 0.12, 0.5, 0.360, 0.360 and 2° scan width, respectively. Crystal data, data collection parameters, and structure refinement details are given in Table S1 in the ESI.† The structures were solved by direct methods and refined by full-matrix least-squares techniques. Non-H atoms were refined with anisotropic displacement parameters. H atoms were inserted in calculated positions and refined with a riding model. The following computer programs and hardware were used: structure solution, SHELXS-2014 and refinement, SHELXL-2014;^54^ molecular diagrams, ORTEP;^55^ computer, Intel CoreDuo. CCDC no.: 2324332 (H_2_L^S^), 2324333 (H_2_L^SEt^), 2324334 (NiL^S^), 2324335 (NiL^SMe^) and 2324336 (NiL^SEt^).†

#### Cyclic voltammetry

All experiments were performed at room temperature under an argon atmosphere. A standard three electrode arrangement of a platinum-disc (from Ionode, Australia) as the working electrode, a platinum wire as the counter electrode, and a silver wire as the pseudoreference electrode was used. The recorded oxidation potentials were obtained with a scan rate of 100 mV s^−1^. Sample solutions with an approximate concentration of 0.5 mM, prepared with 0.2 M *n*Bu_4_NPF_6_ supporting electrolyte in dichloromethane CH_2_Cl_2_, were purged with argon for 5 min before each experiment. The electrochemical measurements were carried out with a Heka PG310USB (Lambrecht, Germany) potentiostat/galvanostat using the PotPulse 8.53 software package. Ferrocene (Fc) and decamethylferrocene (DmFc) from Sigma-Aldrich were used as internal standards.

#### Oxygen evolution study

OER studies were carried out using a Squidstat Plus potentiostat (Admiral Instruments) with platinum mesh as counter electrode and saturated calomel electrode as reference electrode. Potentials are then converted to the reversible hydrogen electrode (RHE) scale. The working electrode was prepared by drop-casting catalytic ink (5 μL; 5 mg of the corresponding Ni(ii) complex in 125 μL of 2 wt% solution of polyvinylidene fluoride in *N*-methyl-2-pyrrolidone) on a conductive glassy carbon support. Linear sweep voltammetry was run at 10 mV s^−1^ in 1 M KOH. Double-layer capacitance was evaluated by recording CVs in non-faradaic region at 5–100 mV s^−1^ rates. Electrochemical impedance spectroscopy was carried out at 1.67 V. Stability was probed by continuous cycling at 10 mV s^−1^ as well as by chronoamperometric measurements at 1.67 V. Graphite rod was used as a counter electrode during these stability measurements. The Raman spectra of working electrode before and after these tests were recorded using a DXR Raman microscope (Thermo Scientific, USA).

## Author contributions

Iuliana Besleaga – data curation; formal analysis; investigation; methodology; writing – original draft; Anastasia A. Fesenko – data curation; investigation; methodology; Anup Paul – data curation; investigation; methodology, sofware, writing-original draft; Biljana Šljukić – investigation; formal analysis; writing – original draft; visualisation; Peter Rapta – investigation; methodology; writing – original draft; Armando J. L. Pombeiro – writing – review and editing; supervision; funding acquisition; Anatoly Shutalev – investigation; writing – review and editing; supervision, and Vladimir B. Arion – conceptualization; funding acquisition; investigation; project administration; writing – review and editing.

## Data availability

The data supporting this article have been included as part of the ESI.† Crystallographic data for compounds H_2_L^S^, H_2_L^SEt^, NiL^S^, NiL^SMe^ and NiL^SEt^ have been deposited at the CCDC under accession numbers 2324332, 2324333, 2324334, 2324335 and 2324336† and can be obtained from CCDC e-mail: deposit@ccdc.cam.ac.uk.

## Conflicts of interest

The authors declare no competing financial interest.

## Supplementary Material

DT-053-D4DT02182G-s001

DT-053-D4DT02182G-s002
