# Supplementary material for: Nickel(ii) complexes with 14-membered bis-thiosemicarbazide and bis-isothiosemicarbazide ligands: synthesis, characterization and catalysis of oxygen evolution reaction
Source: Dalton Trans. 2024 Aug 27;53(38):15826–41. doi: 10.1039/d4dt02182g (PMC11375789; doi:10.1039/d4dt02182g)
Supplement: DT-053-D4DT02182G-s001 [file DT-053-D4DT02182G-s001.pdf]

Supplementary Information (SI) for Dalton Transactions.
This journal is © The Royal Society of Chemistry 2024

Supporting Information

for

Nickel(II) Complexes with 14-membered bis-Thiosemicarbazide and bis-Isothiosemicarbazide Ligands: Synthesis, Characterization and Catalysis of Oxygen Evolution Reaction

Iuliana Besleaga,[a] Anastasia A. Fesenko,[b] Anup Paul,[c] Biljana Šljukić,[d] Peter Rapta,[e,*] Armando J. L. Pombeiro,[c,*] Anatoly D. Shutalev,[f,*] Vladimir B. Arion [a,g,*]

[a]*University of Vienna, Institute of Inorganic Chemistry, Währinger Strasse 42, A-1090 Vienna, Austria*

[b]*A. N. Frumkin Institute of Physical Chemistry and Electrochemistry, Russian Academy of Sciences, 31 Leninsky Ave., 119071 Moscow, Russian Federation*

[c]*Centro de Química Estrutural, Institute of Molecular Sciences, Instituto Superior Técnico, Universidade de Lisboa, Av. Rovisco Pais, 1049-001 Lisboa, Portugal*

[d]*Center of Physics and Engineering of Advanced Materials, Laboratory of Physics of Materials and Emerging Technologies, Chemical Engineering Department, Instituto Superior Técnico, Universidade de Lisboa, 1049-001, Lisbon, Portugal*

[e]*Institute of Physical Chemistry and Chemical Physics, Faculty of Chemical and Food Technology, Slovak University of Technology in Bratislava, Radlinského 9, SK-81237 Bratislava, Slovak Republic*

[f]*N. D. Zelinsky Institute of Organic Chemistry, Russian Academy of Sciences, 47 Leninsky Ave., 119991 Moscow, Russian Federation*

[g]*Inorganic Polymers Department, "Petru Poni" Institute of Macromolecular Chemistry, Aleea Gr. Ghica Voda 41 A, Iasi 700487, Romania*

Table of contents

1. Characterization of H_2L^S and H_2L^{SEt} 3

1.1. NMR spectra of the ligands 3

1.2. ESI-MS of the ligands 26

1.3. IR spectra of the ligands 28

2. Characterization of Ni(II) complexes 30

3. Crystallographic data 43

1. Characterization of H_2L^S and H_2L^{SEt}

1.1. NMR spectra of the ligands

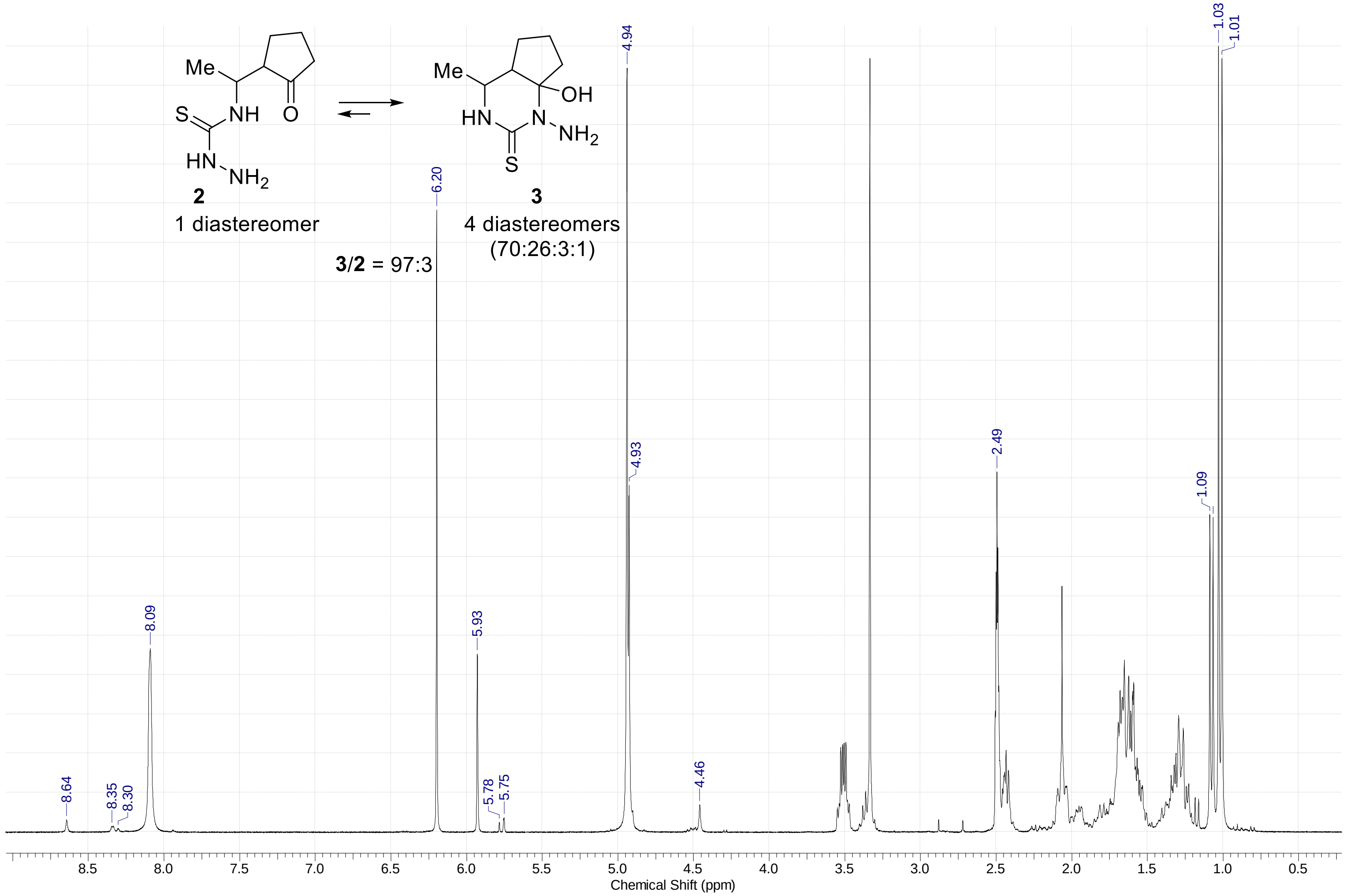

Figure S1. ^{1}H NMR spectrum of the starting material (mixture of **3** and **2**) (300.13 MHz, DMSO-d_6)

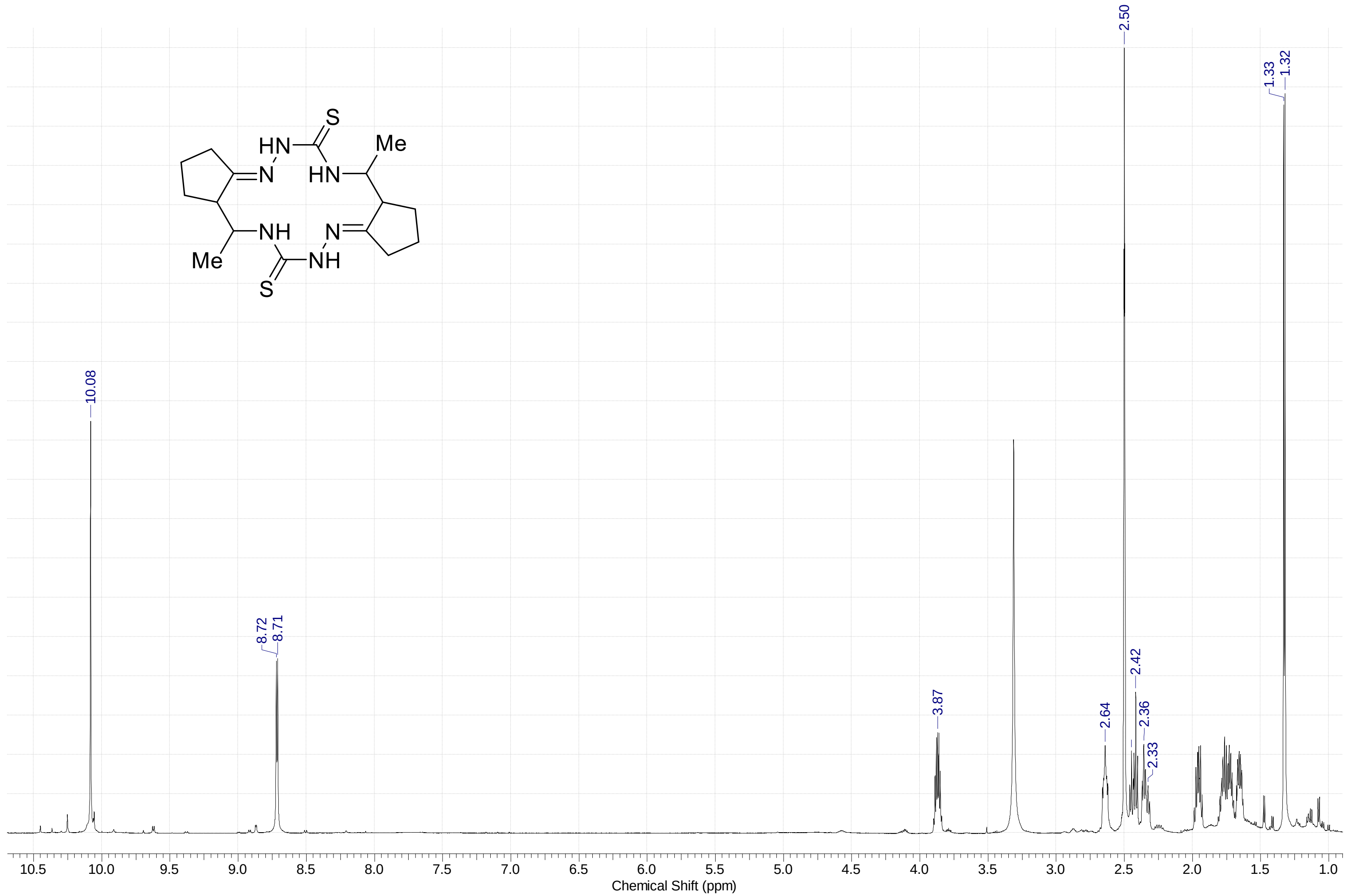

Figure S2. ^{1}H NMR spectrum of crude **6** prepared by the treatment of the mixture of **3** and **2** with $NH_2OH{\cdot}HCl$ (1.25 equiv) (EtOH, reflux, 2 h) (600.13 MHz, DMSO-d_6)

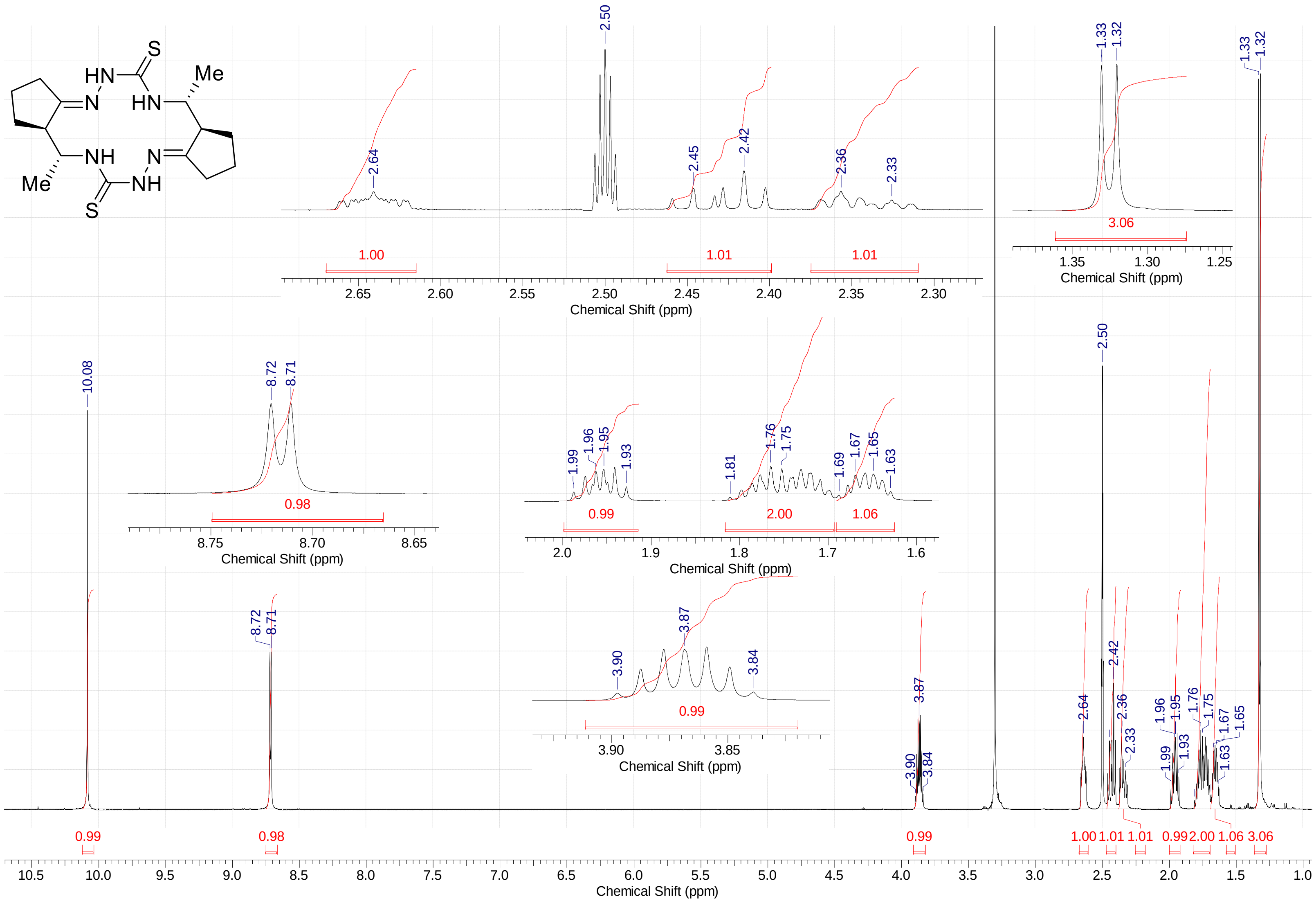

Figure S3A. ^{1}H NMR spectrum of (5*R**,6*R**,12*R**,13*R**)**-6** (600.13 MHz, DMSO-d_6)

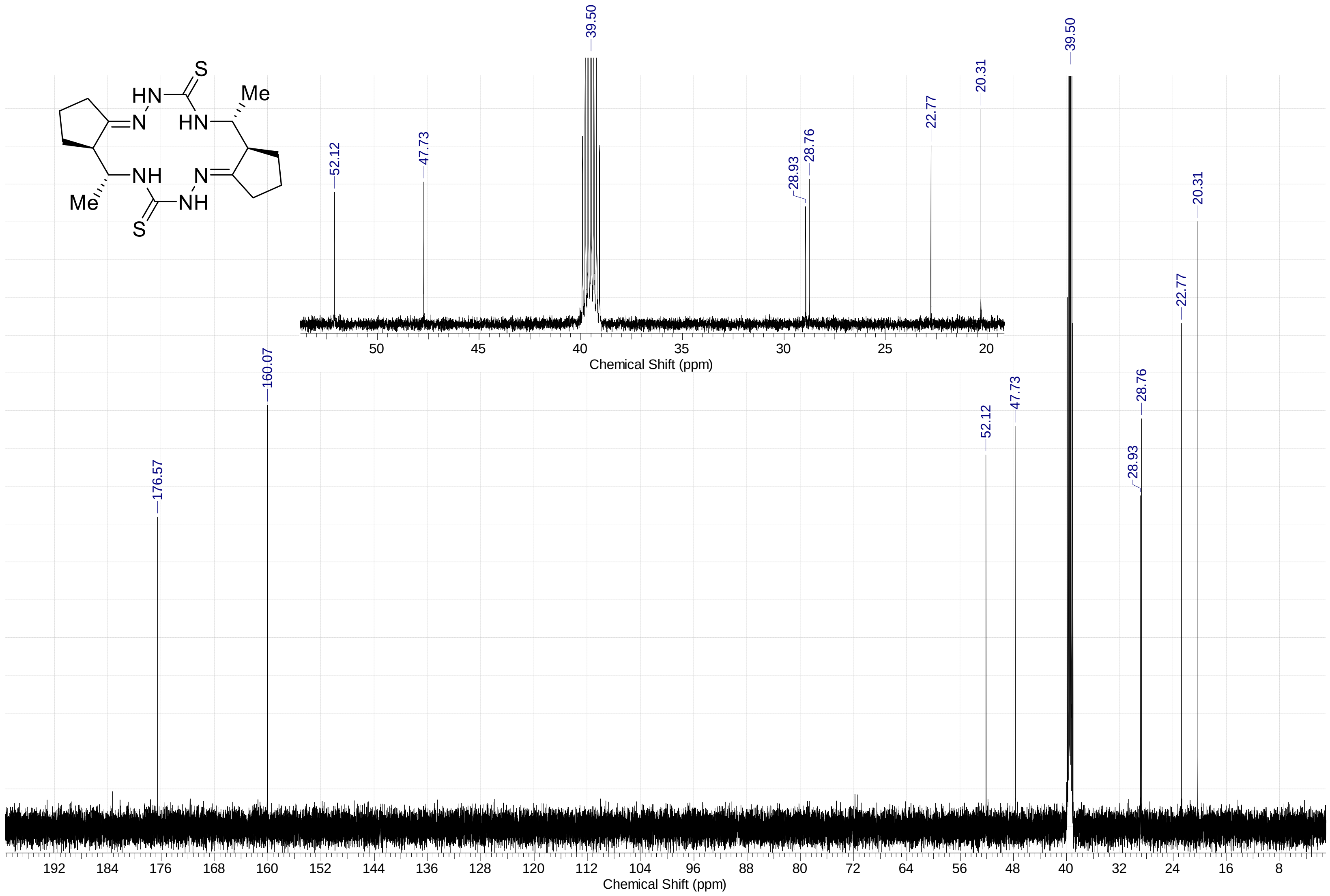

Figure S3B. ^{13}C NMR spectrum of (5*R**,6*R**,12*R**,13*R**)-**6** (150.90 MHz, DMSO-d_6)

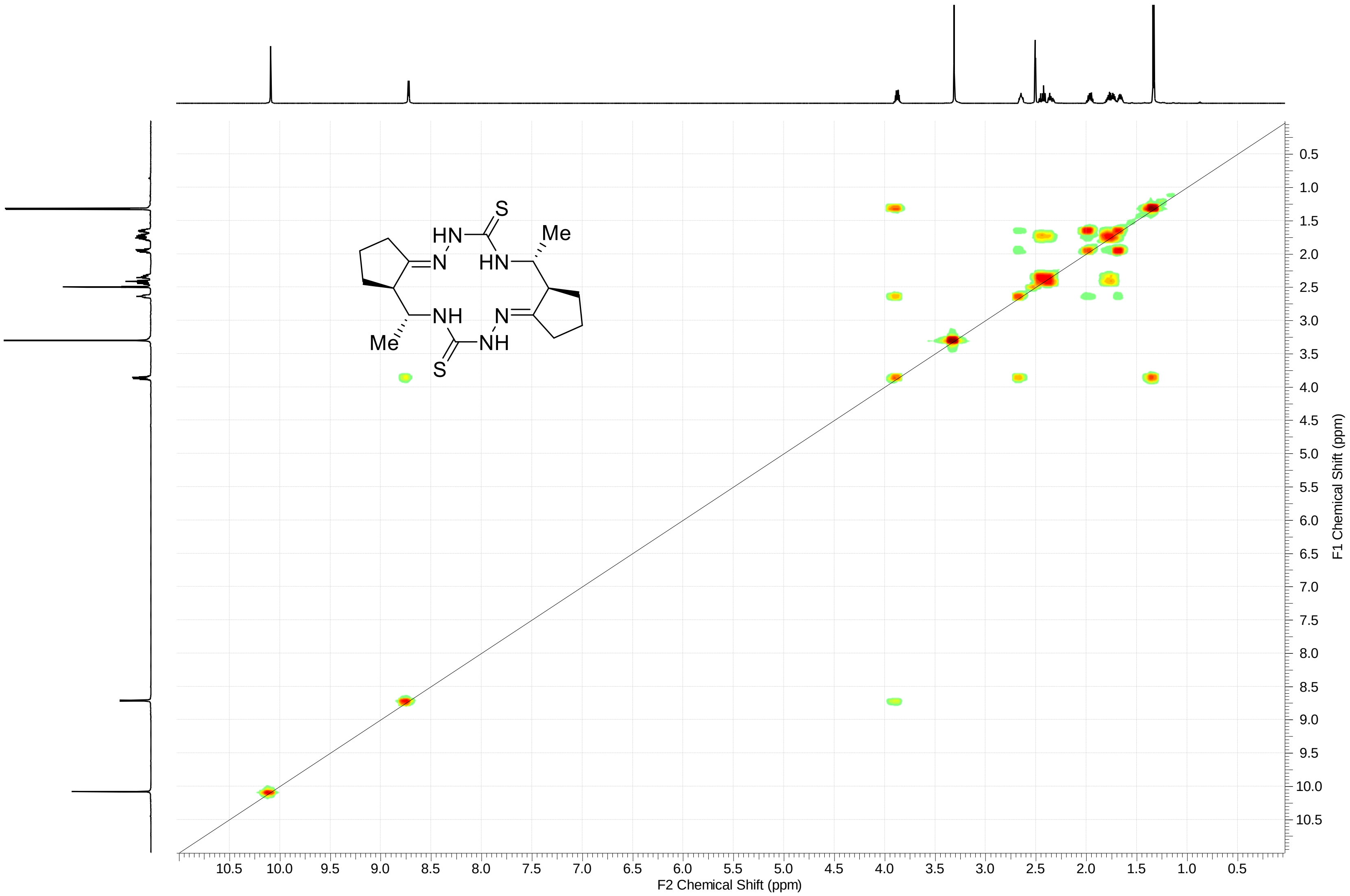

Figure S3C. ^{1}H,^{1}H COSY spectrum of (5*R**,6*R**,12*R**,13*R**)-**6** (150.90 MHz, DMSO-d_6)

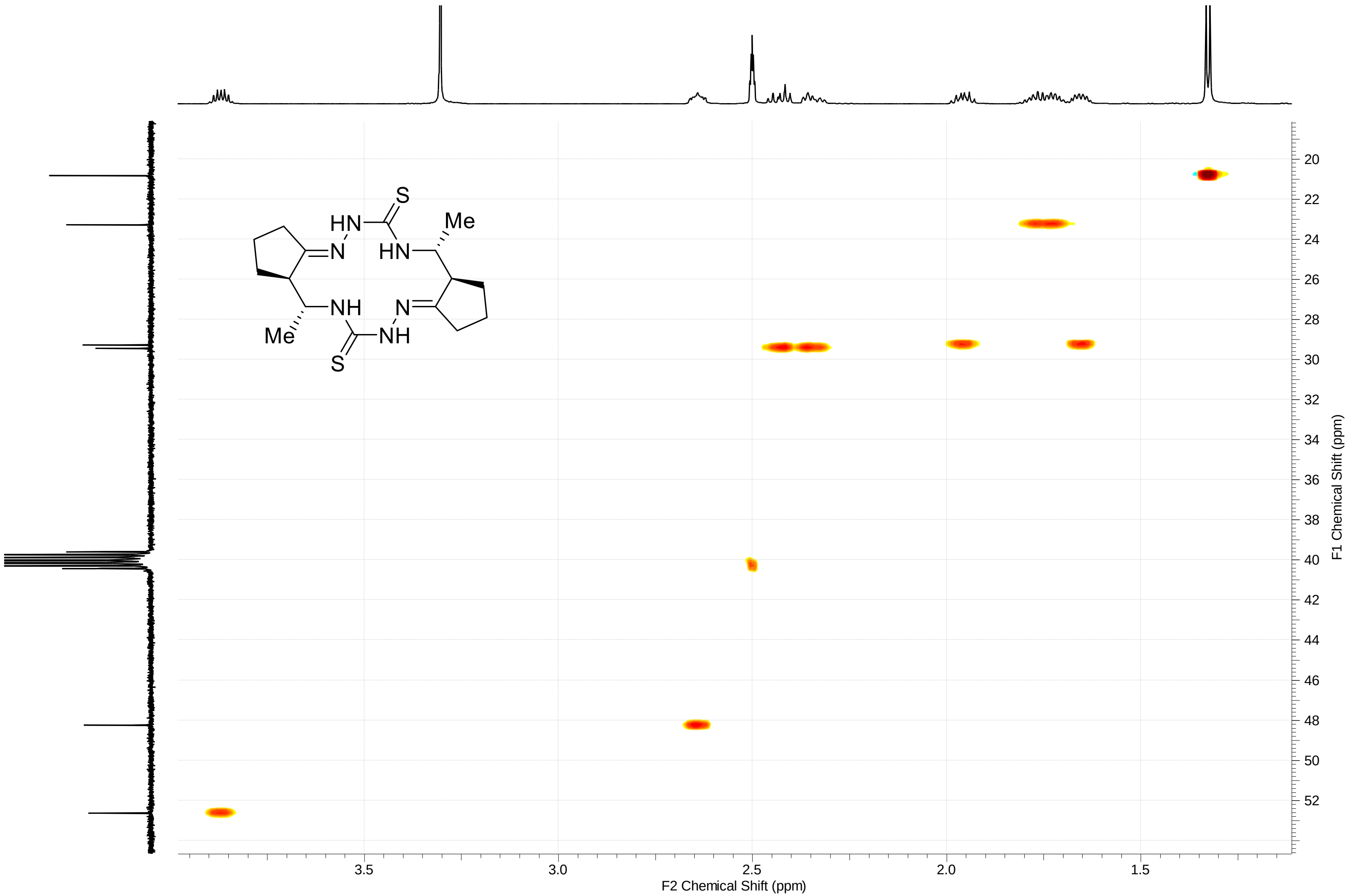

Figure S3D. $^{1}H,^{13}C$ HSQC spectrum of (5*R**,6*R**,12*R**,13*R**)-**6** (Bruker Avance III, DMSO-d_6)

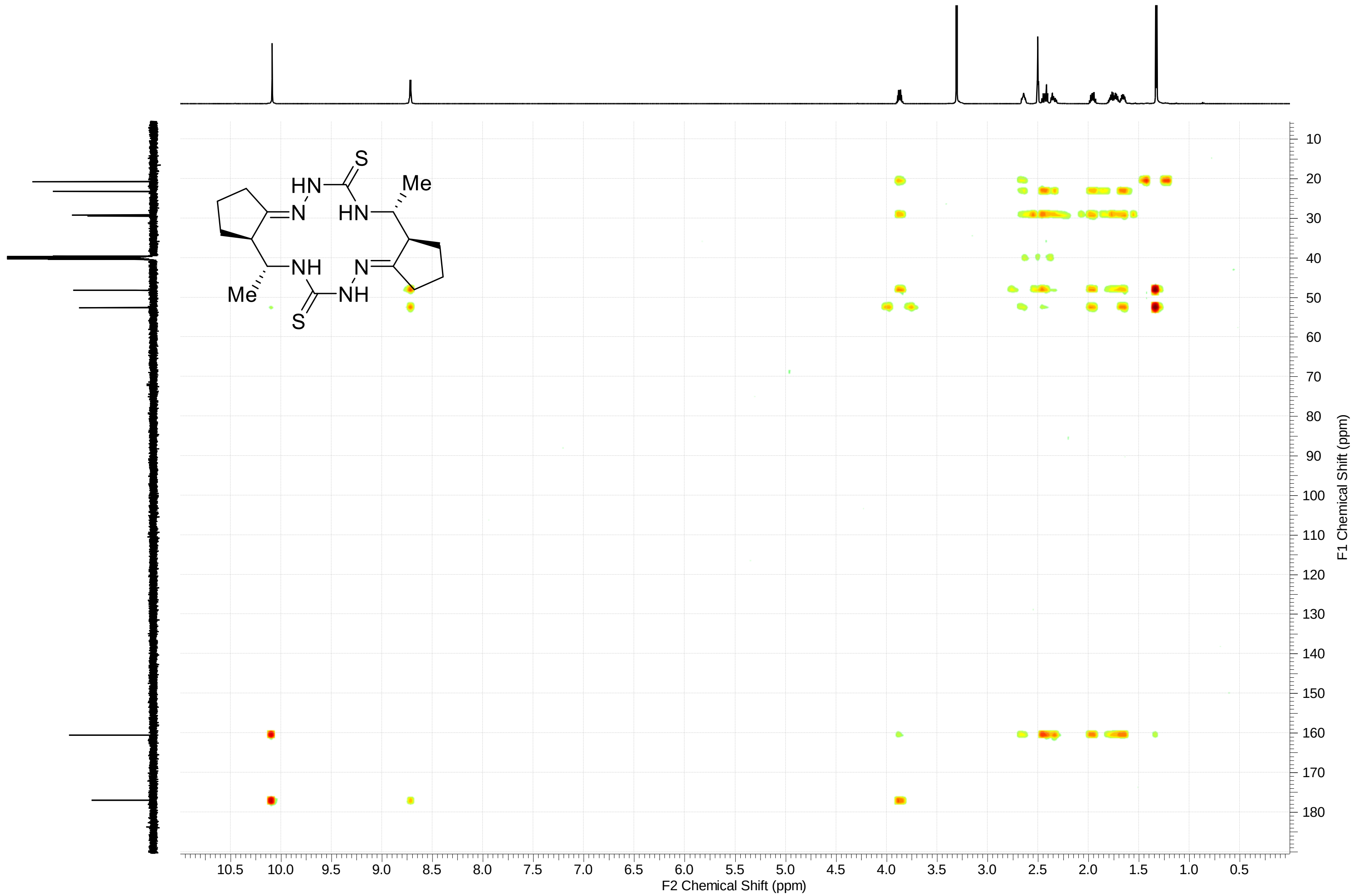

Figure S3E. $^{1}H,^{13}C$ HMBC spectrum of (5*R**,6*R**,12*R**,13*R**)**-6** (Bruker Avance III, DMSO-d_6)

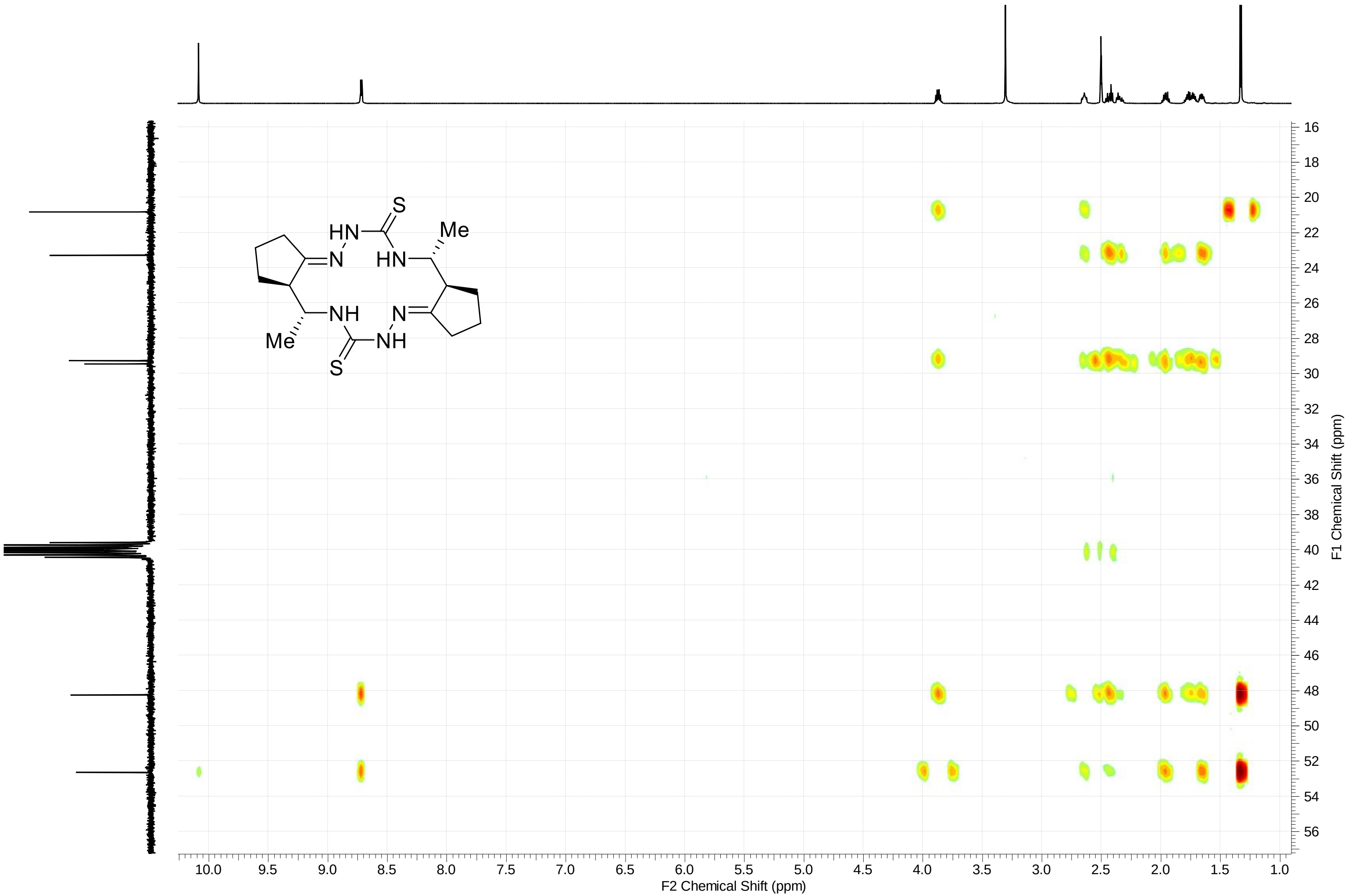

Figure S3F. Fragment of ^{1}H,^{13}C HMBC spectrum of (5*R**,6*R**,12*R**,13*R**)-**6** (Bruker Avance III, DMSO-d_6)

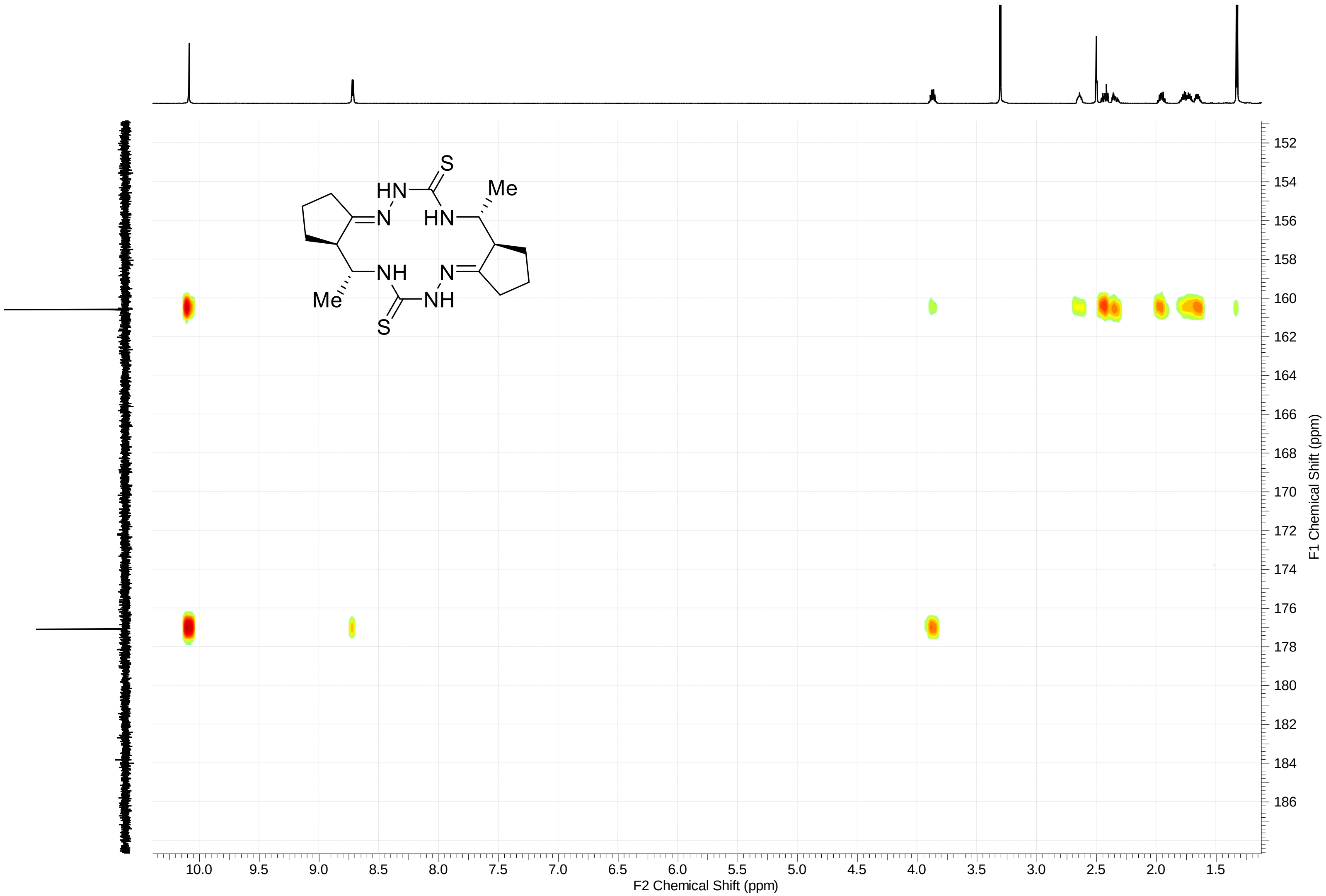

Figure S3G. Fragment of ^{1}H,^{13}C HMBC spectrum of (5*R**,6*R**,12*R**,13*R**)-**6** (Bruker Avance III, DMSO-d_6)

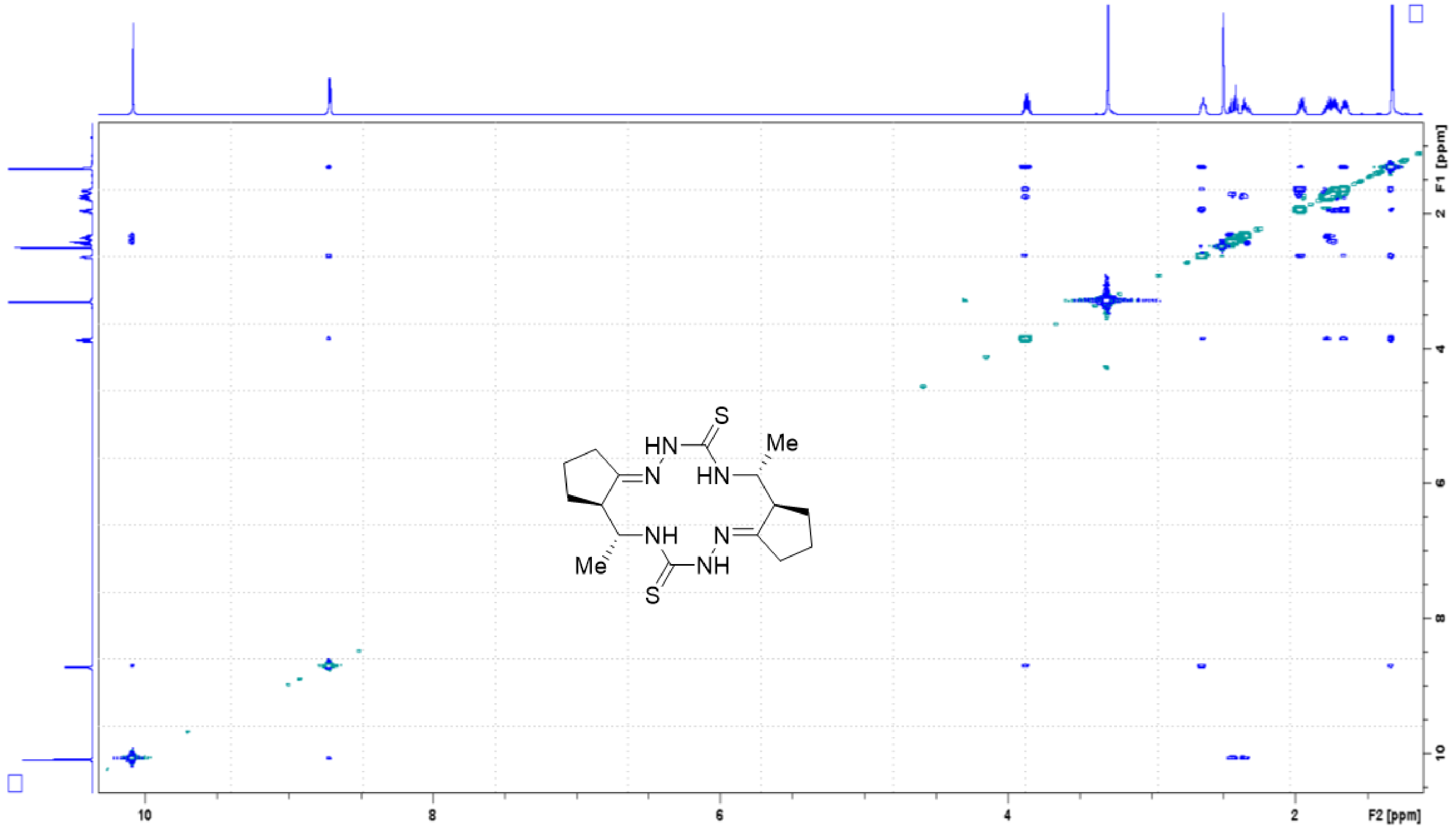

Figure S3H. $^{1}H,^{1}H$ NOESY spectrum of (5*R**,6*R**,12*R**,13*R**)-**6** (600.13 MHz, DMSO-d_6)

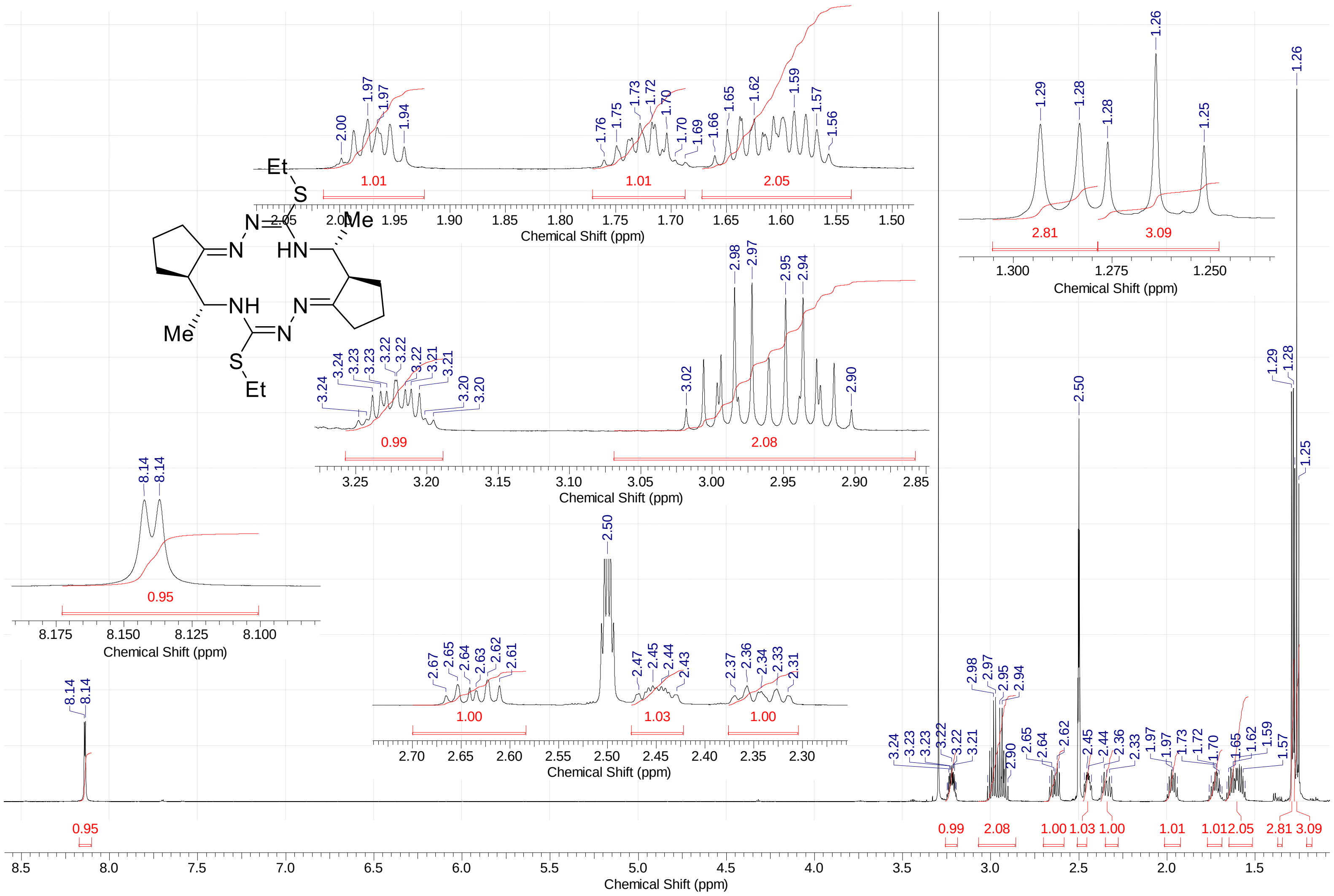

Figure S4A. ^{1}H NMR spectrum of (5*R**,6*R**,12*R**,13*R**)-**8** (600.13 MHz, DMSO-d_6)

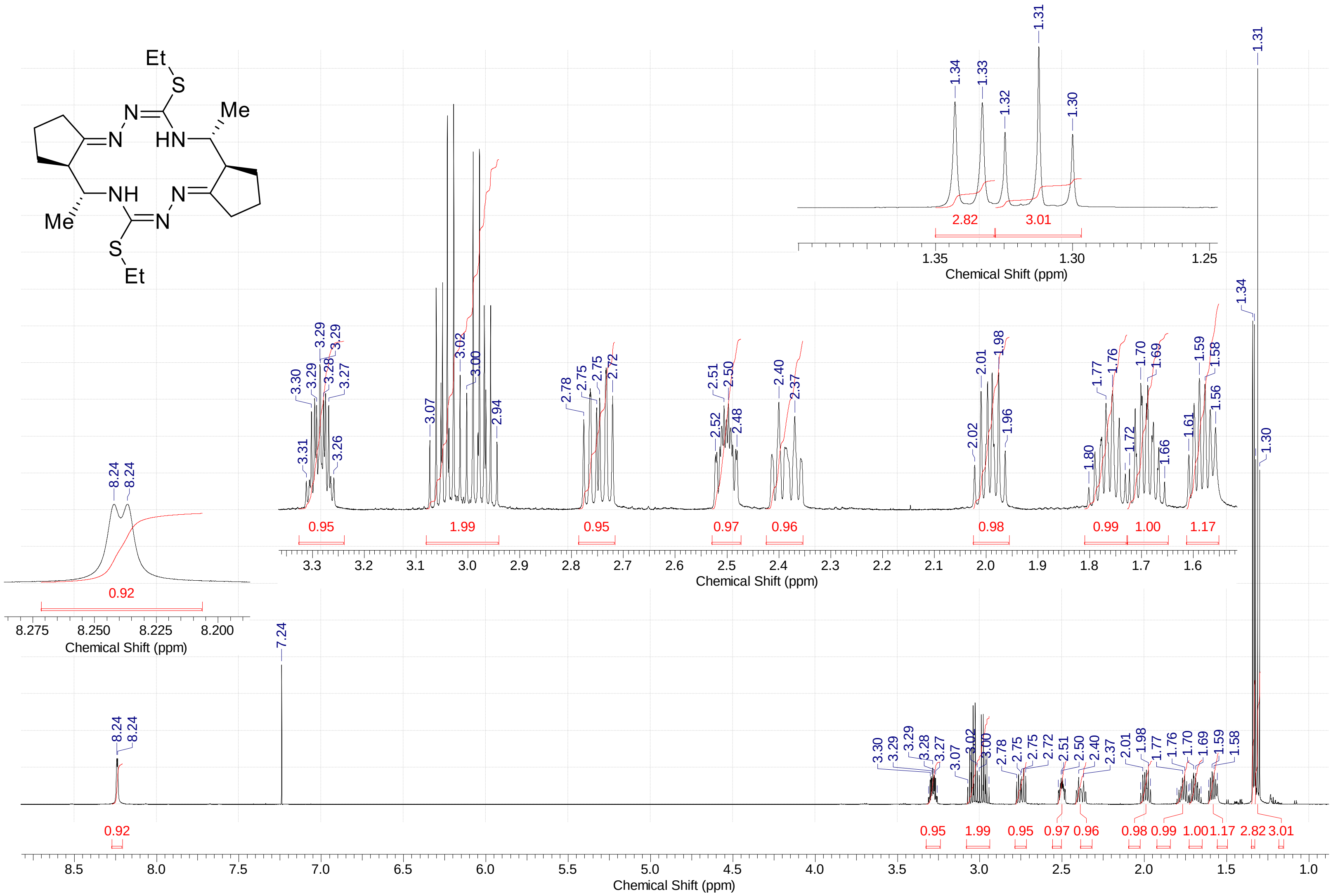

Figure S4B. ^{1}H NMR spectrum of (5*R**,6*R**,12*R**,13*R**)-**8** (600.13 MHz, $CDCl_3$).

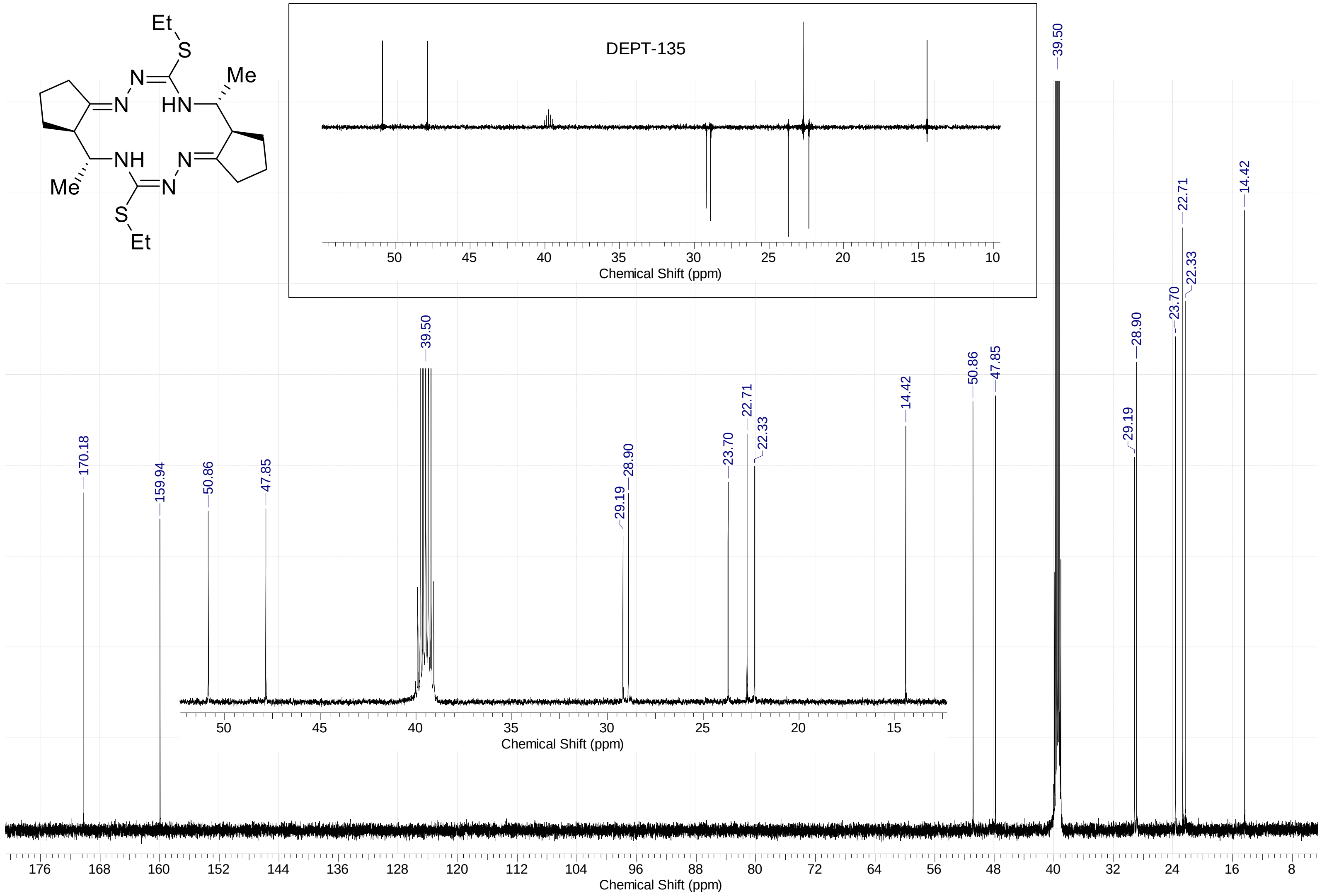

Figure S4C. ^{13}C NMR spectrum of (5*R**,6*R**,12*R**,13*R**)-**8** (150.90 MHz, DMSO-d_6).

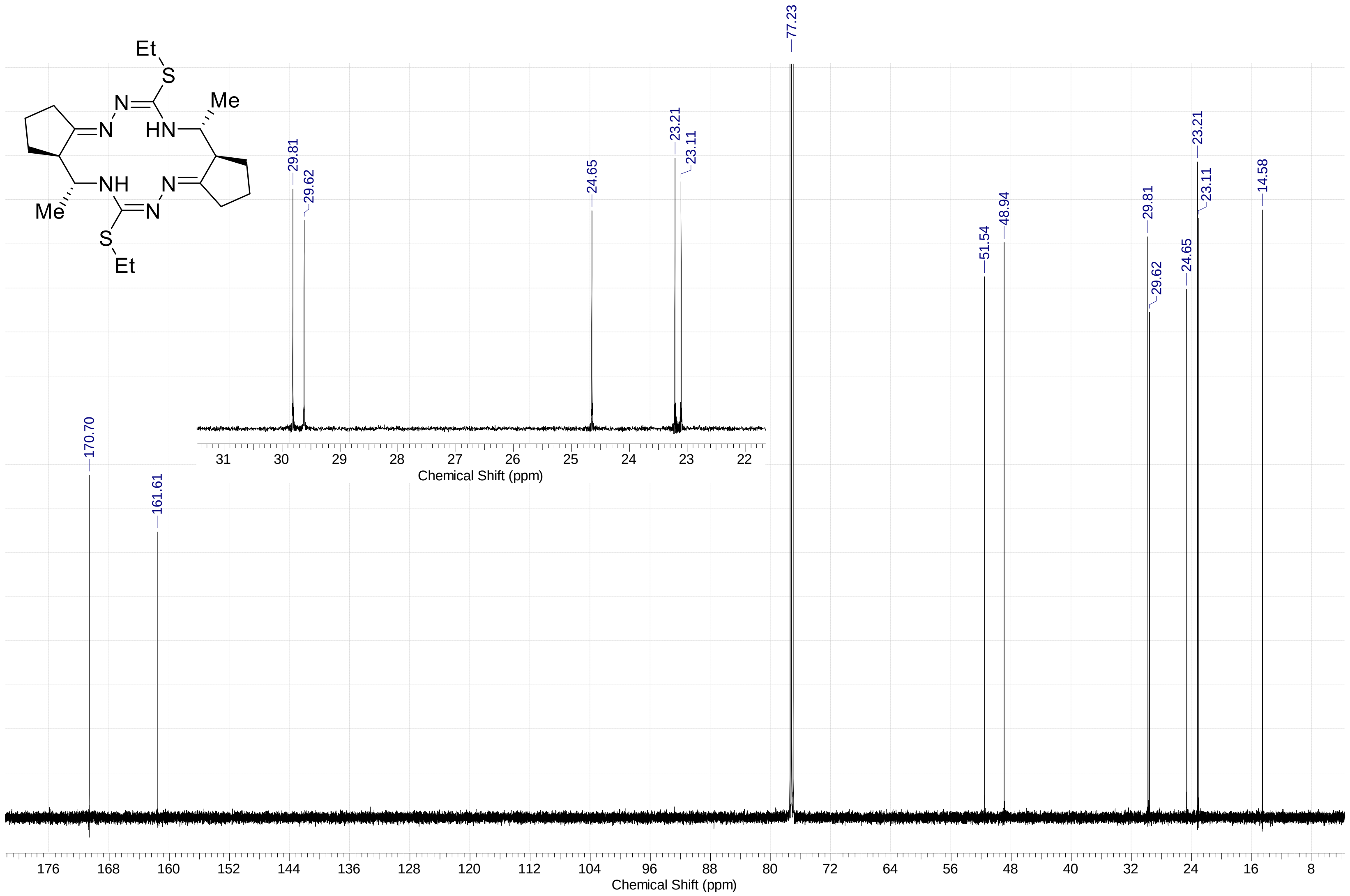

Figure S4D. ^{13}C NMR spectrum of (5*R**,6*R**,12*R**,13*R**)**-8** (150.90 MHz, $CDCl_3$).

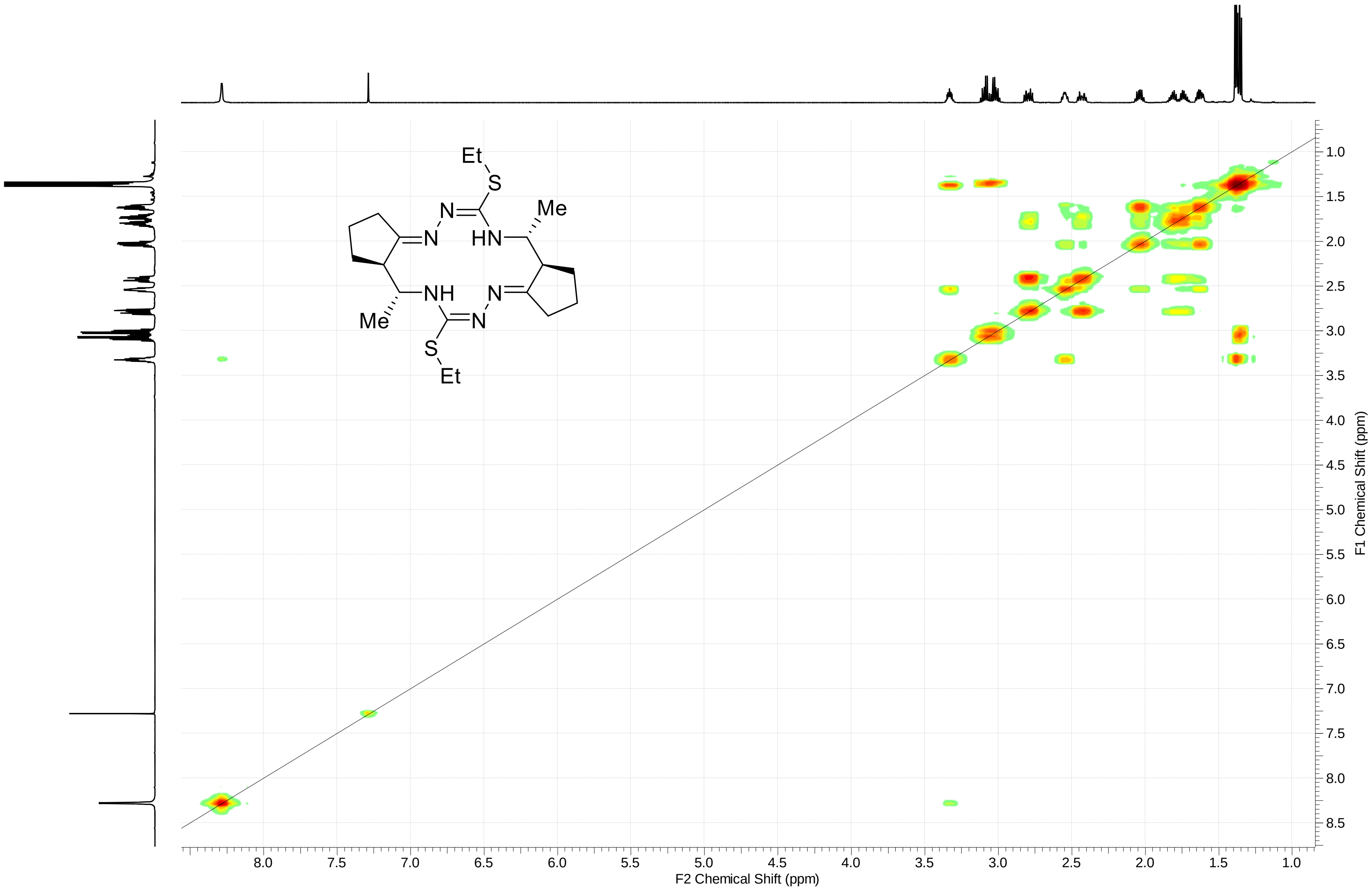

Figure S4E. ^{1}H,^{1}H COSY spectrum of (5*R**,6*R**,12*R**,13*R**)-**8** (150.90 MHz, $CDCl_3$).

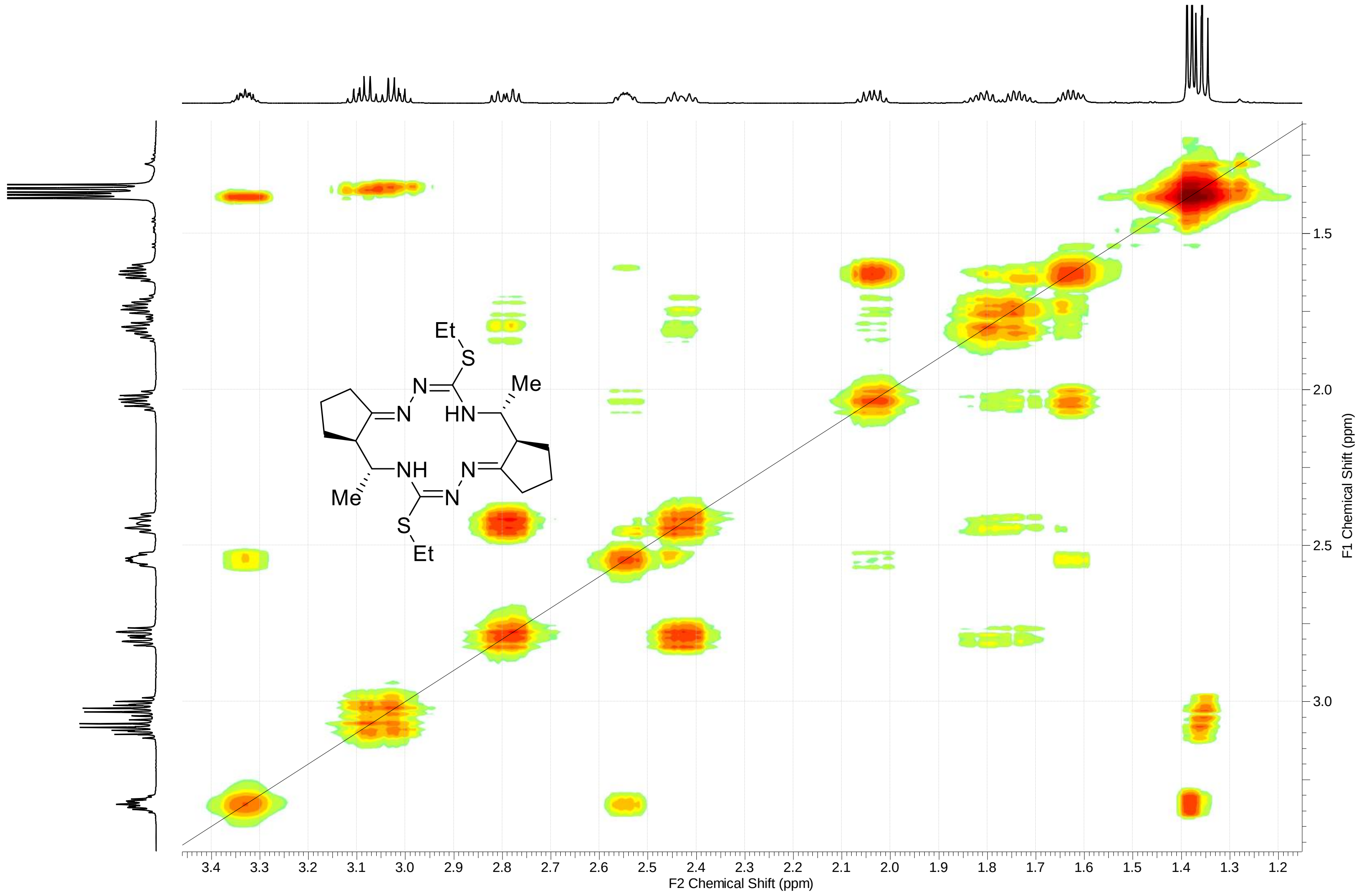

Figure S4F. Fragment of ^{1}H,^{1}H COSY spectrum of (5*R**,6*R**,12*R**,13*R**)**-8** (150.90 MHz, $CDCl_3$).

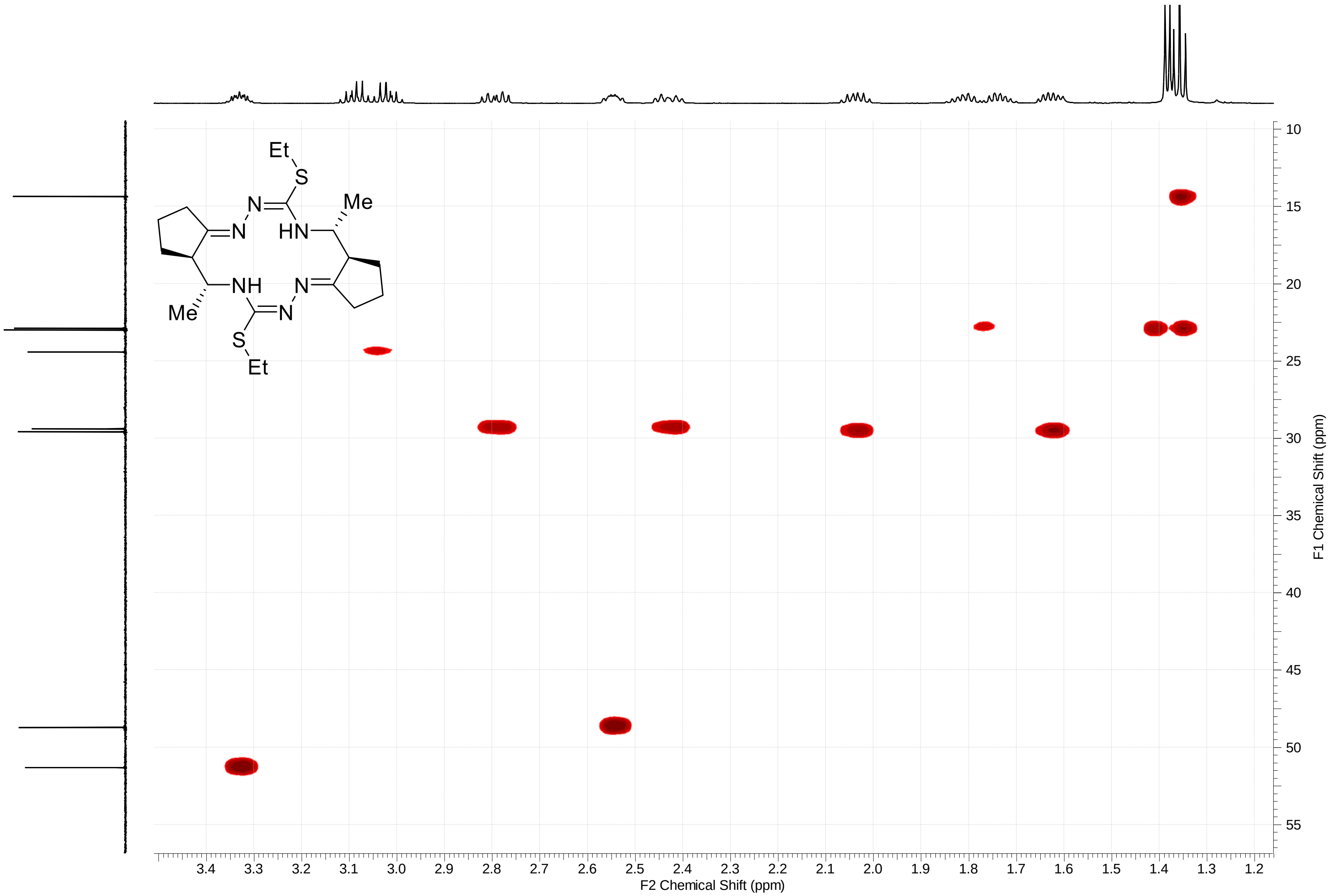

Figure S4G. ^{1}H,^{13}C HSQC spectrum of (5*R**,6*R**,12*R**,13*R**)-**8** (Bruker Avance III, $CDCl_3$).

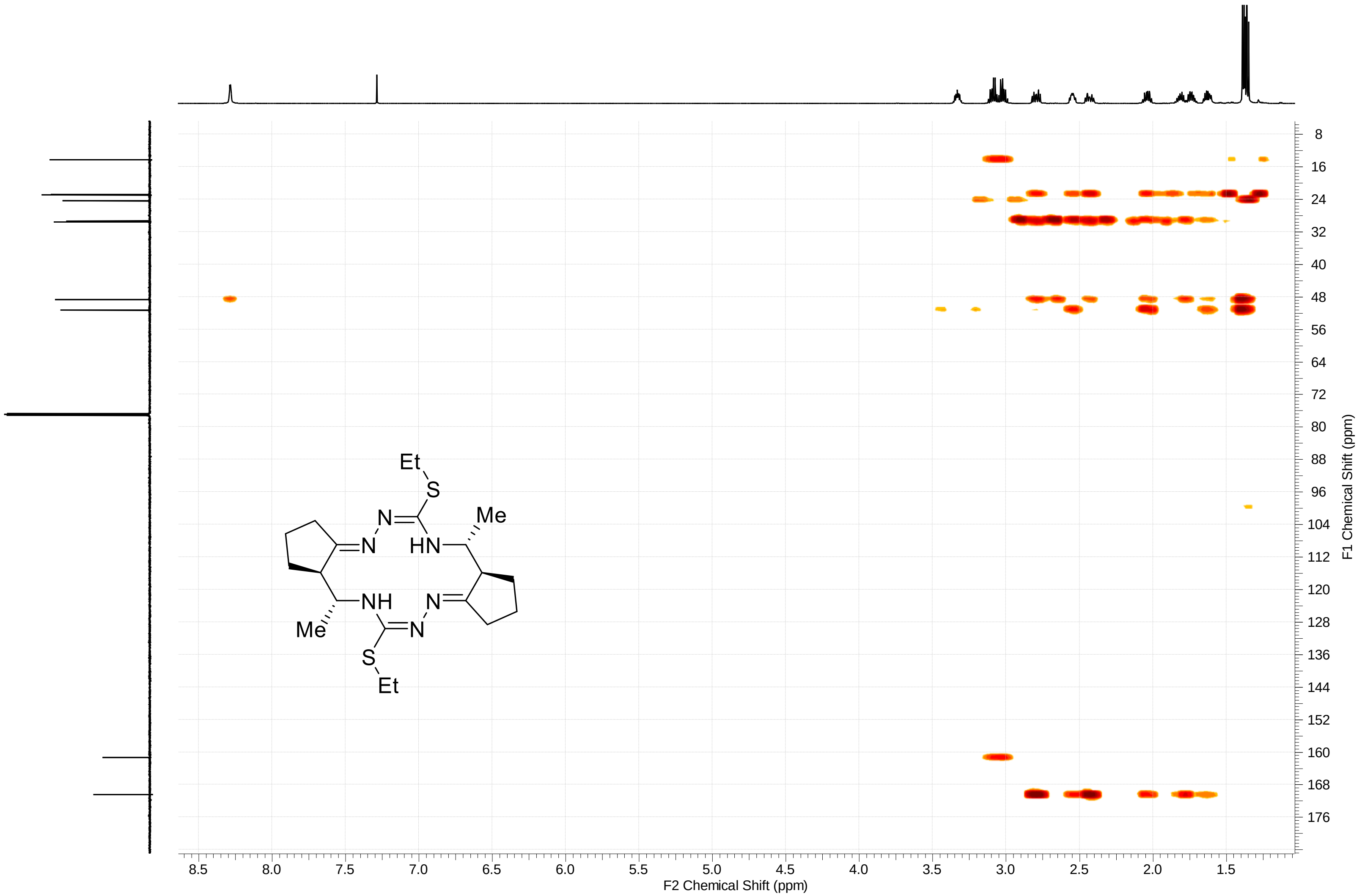

Figure S4H. ^{1}H,^{13}C HMBC spectrum of (5*R**,6*R**,12*R**,13*R**)-**8** (Bruker Avance III, $CDCl_3$).

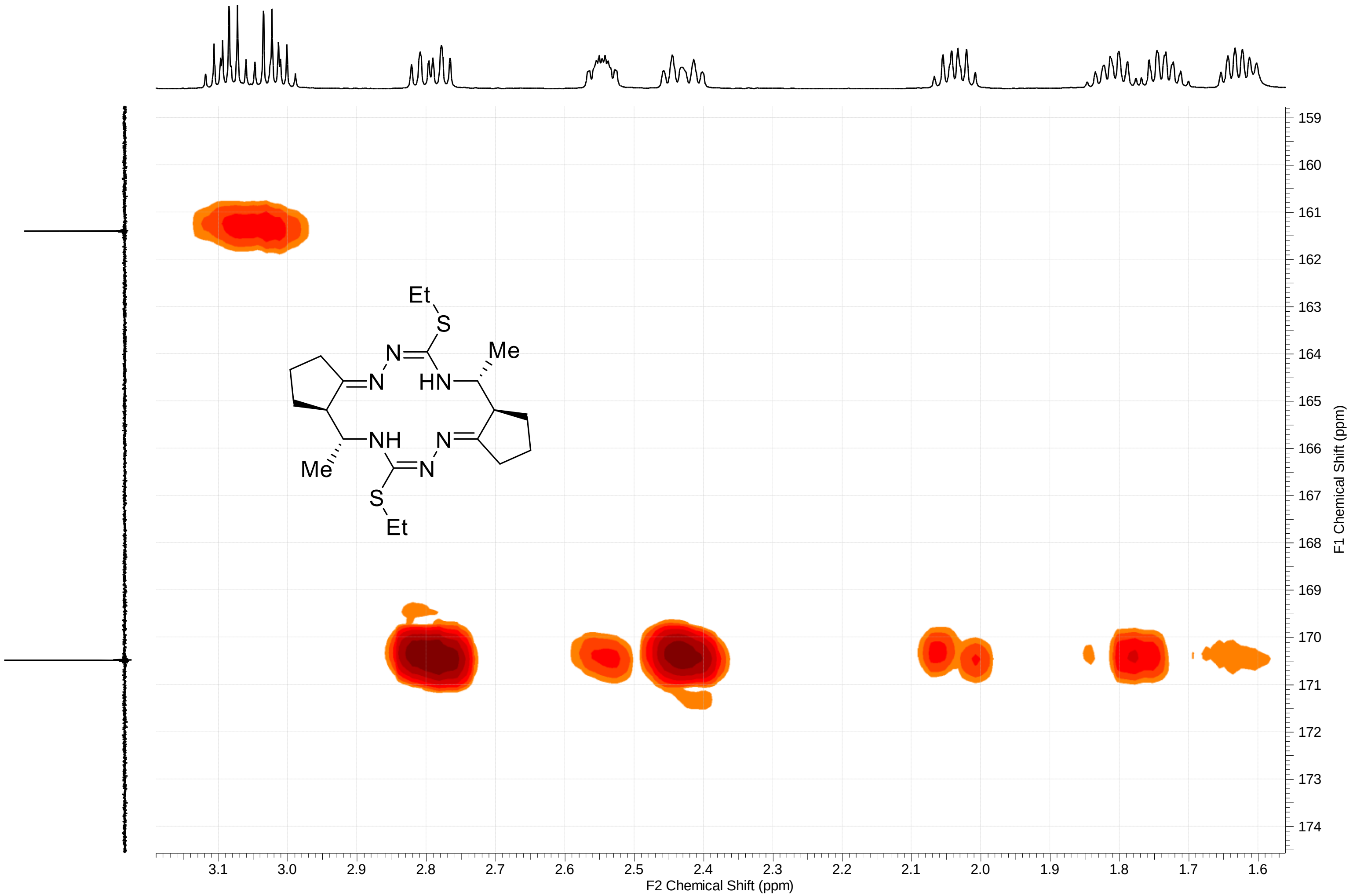

Figure S4I. Fragment of $^{1}H,^{13}C$ HMBC spectrum of (5*R**,6*R**,12*R**,13*R**)-**8** (Bruker Avance III, $CDCl_3$).

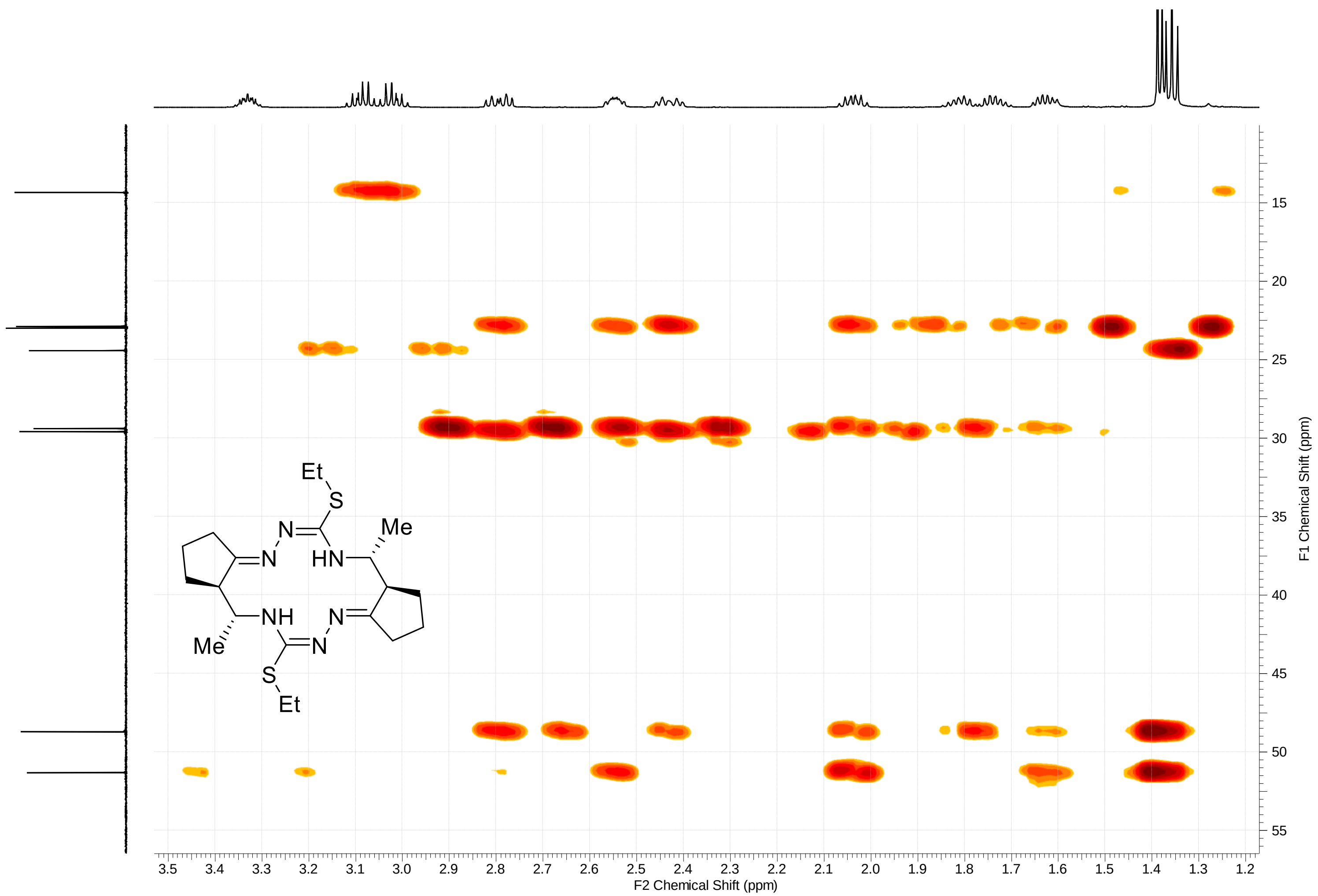

Figure S4J. Fragment of $^{1}H,^{13}C$ HMBC spectrum of (5*R**,6*R**,12*R**,13*R**)-**8** (Bruker Avance III, $CDCl_3$).

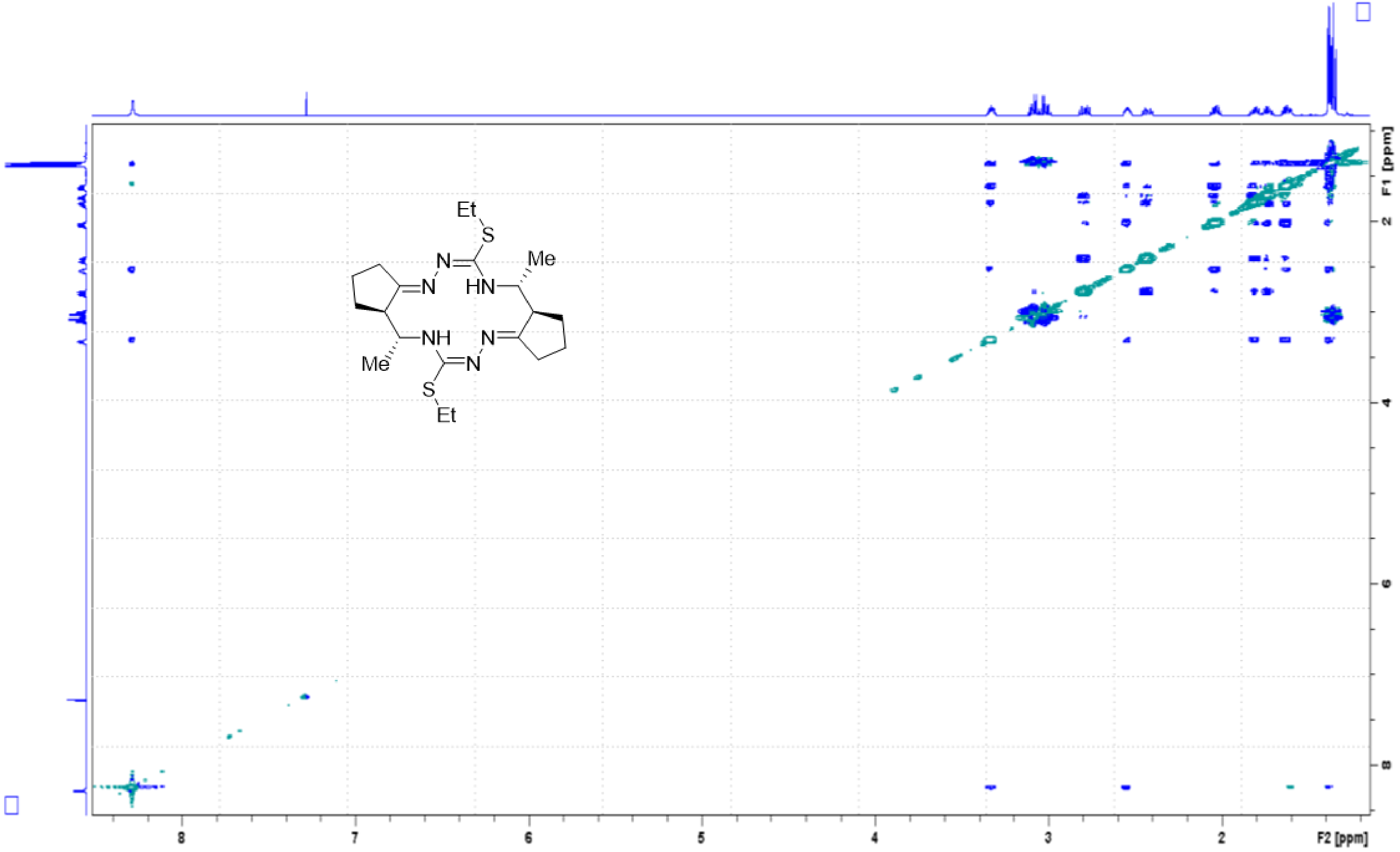

Figure S4K. $^{1}H,^{1}H$ NOESY spectrum of (5*R**,6*R**,12*R**,13*R**)-**8** (600.13 MHz, $CDCl_3$).

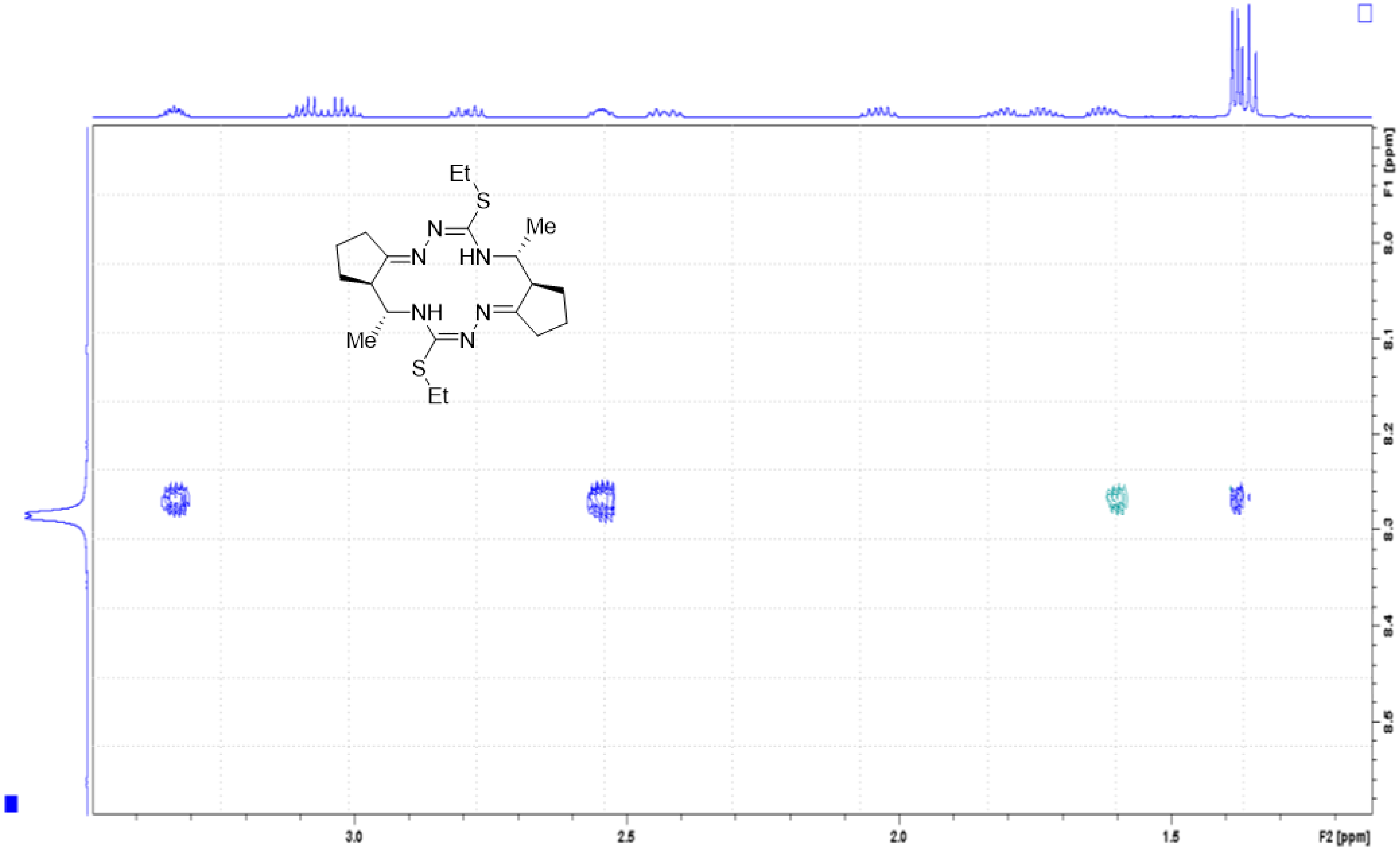

Figure S4L. Fragment of ^{1}H,^{1}H NOESY spectrum of (5*R**,6*R**,12*R**,13*R**)**-8** (600.13 MHz, $CDCl_3$).

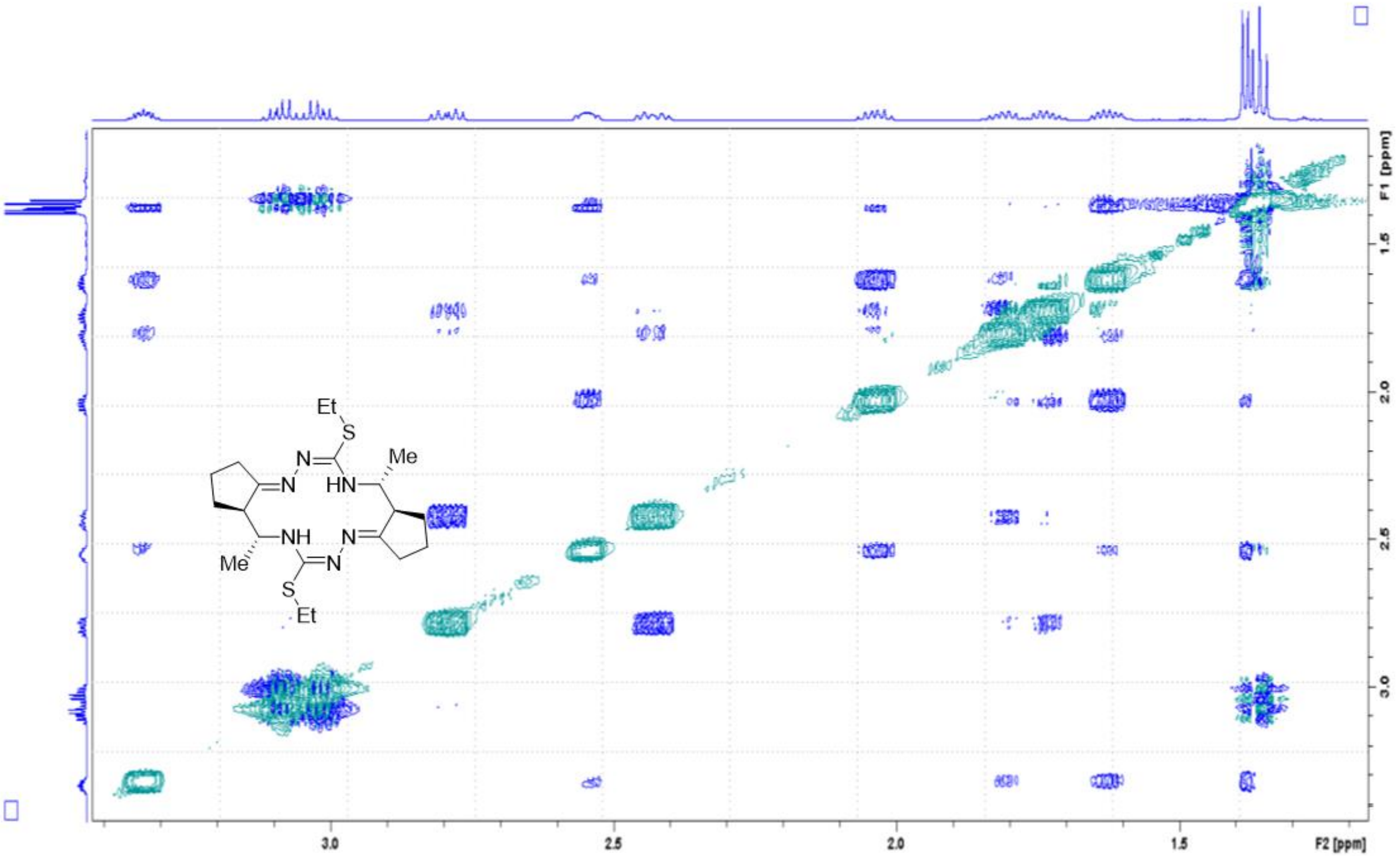

Figure S4M. Fragment of ^{1}H,^{1}H NOESY spectrum of (5*R**,6*R**,12*R**,13*R**)**-8** (600.13 MHz, $CDCl_3$).

1.2. ESI-MS of the ligands

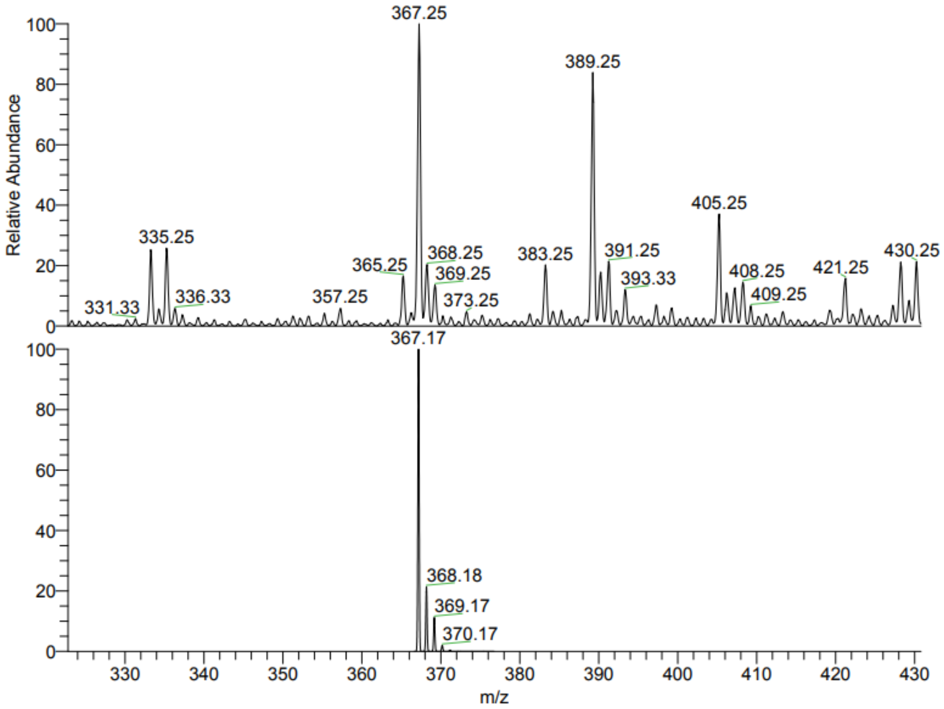

Figure S5. Positive ion ESI-MS for $\mathbf{H_2L^S}$.

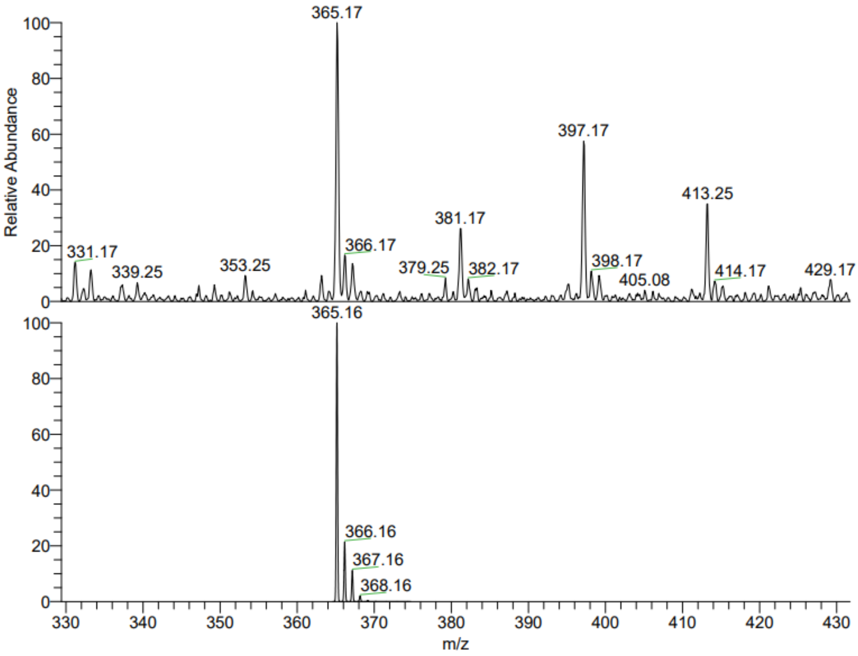

Figure S6. Negative ion ESI-MS for $\mathbf{H_2L^S}$.

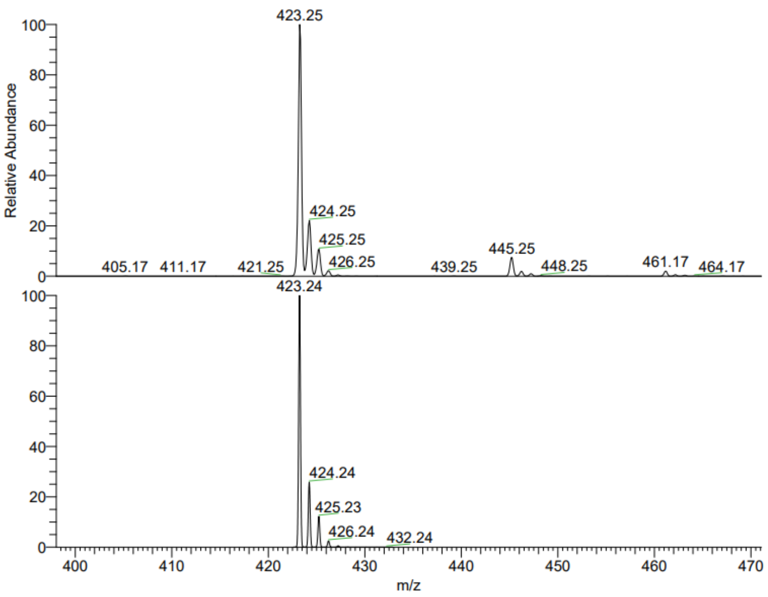

Figure S7. Positive ion ESI-MS for $\mathbf{H_2L^{SEt}}$.

1.3. IR spectra of the ligands

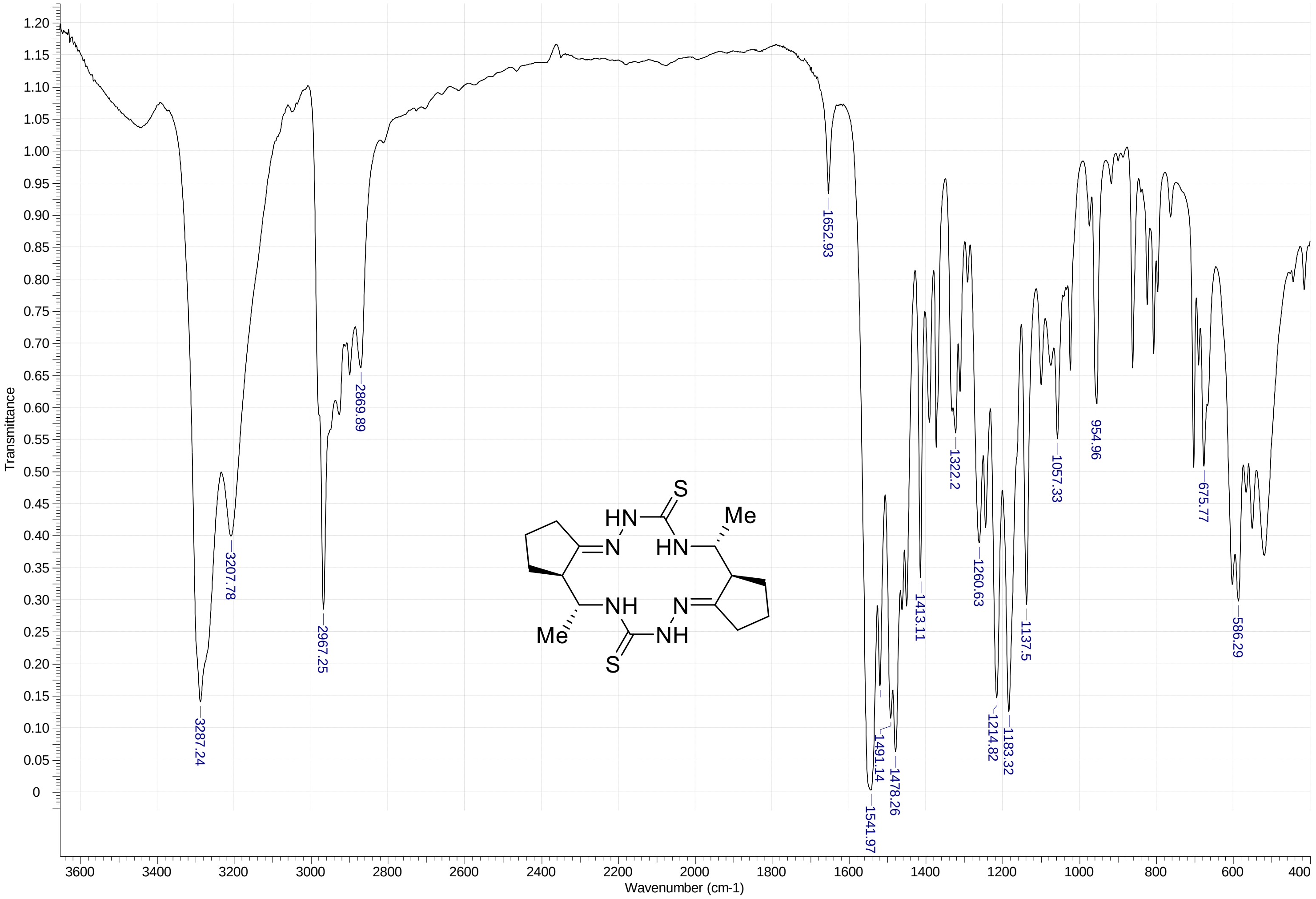

Figure S8. IR spectrum of (5*R**,6*R**,12*R**,13*R**)-**6** (KBr).

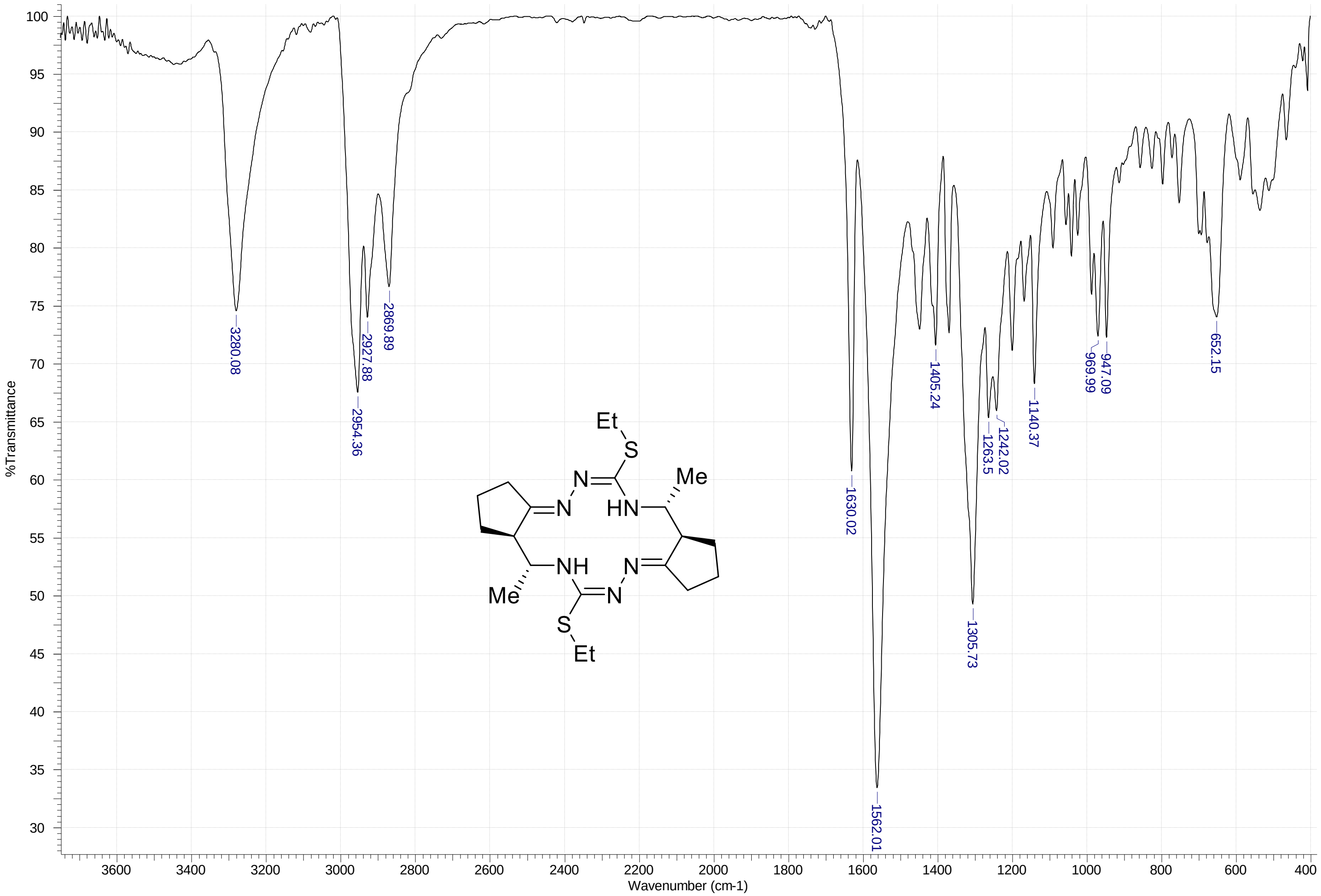

Figure S9. IR spectrum of (5*R**,6*R**,12*R**,13*R**)**-8** (KBr).

2. Characterization of Ni(II) complexes

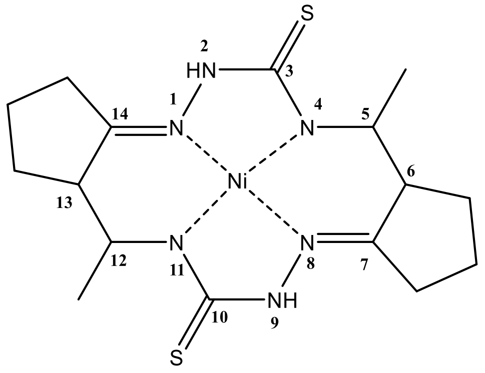

Scheme S1. The atom numbering for the assignment of resonances of Ni(II) complexes.

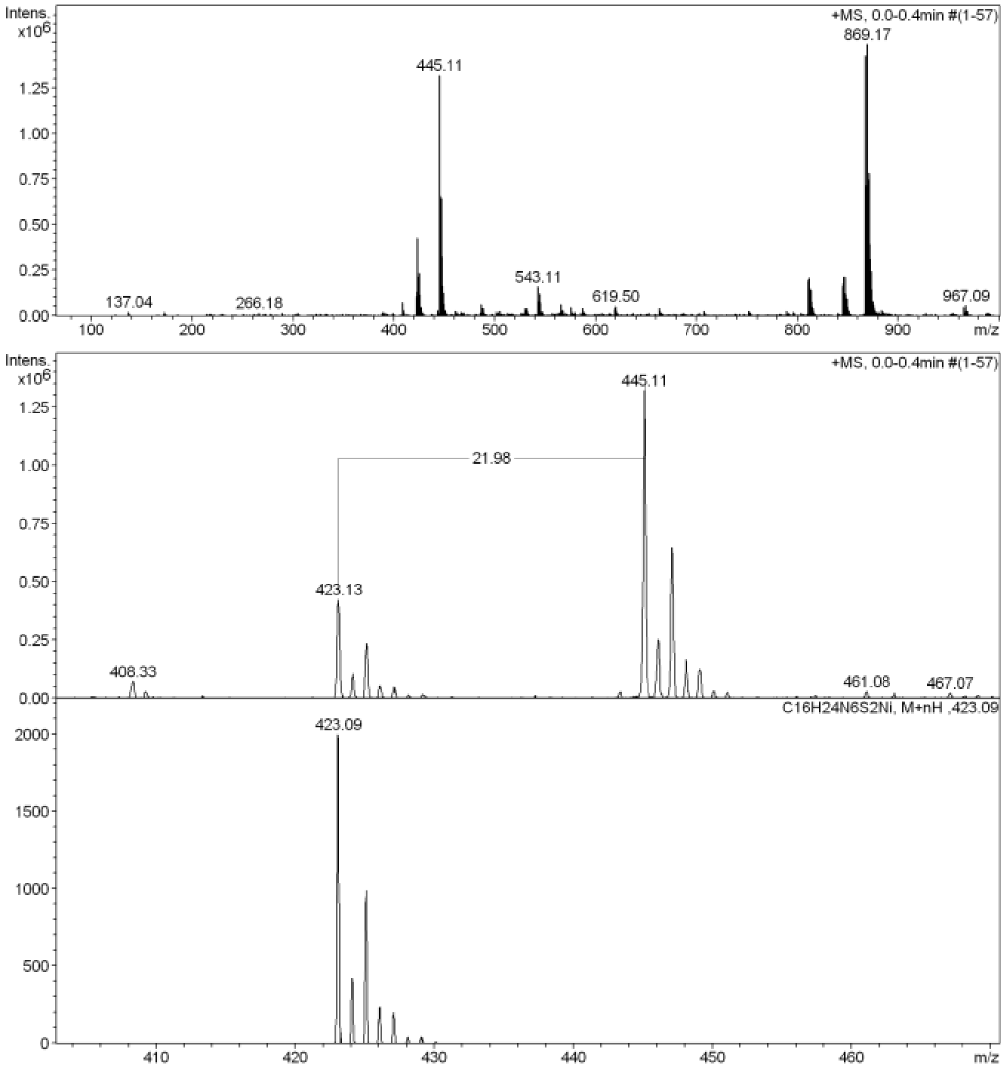

Figure S10. Positive ion ESI-MS for **Ni^{II}L^{S}**.

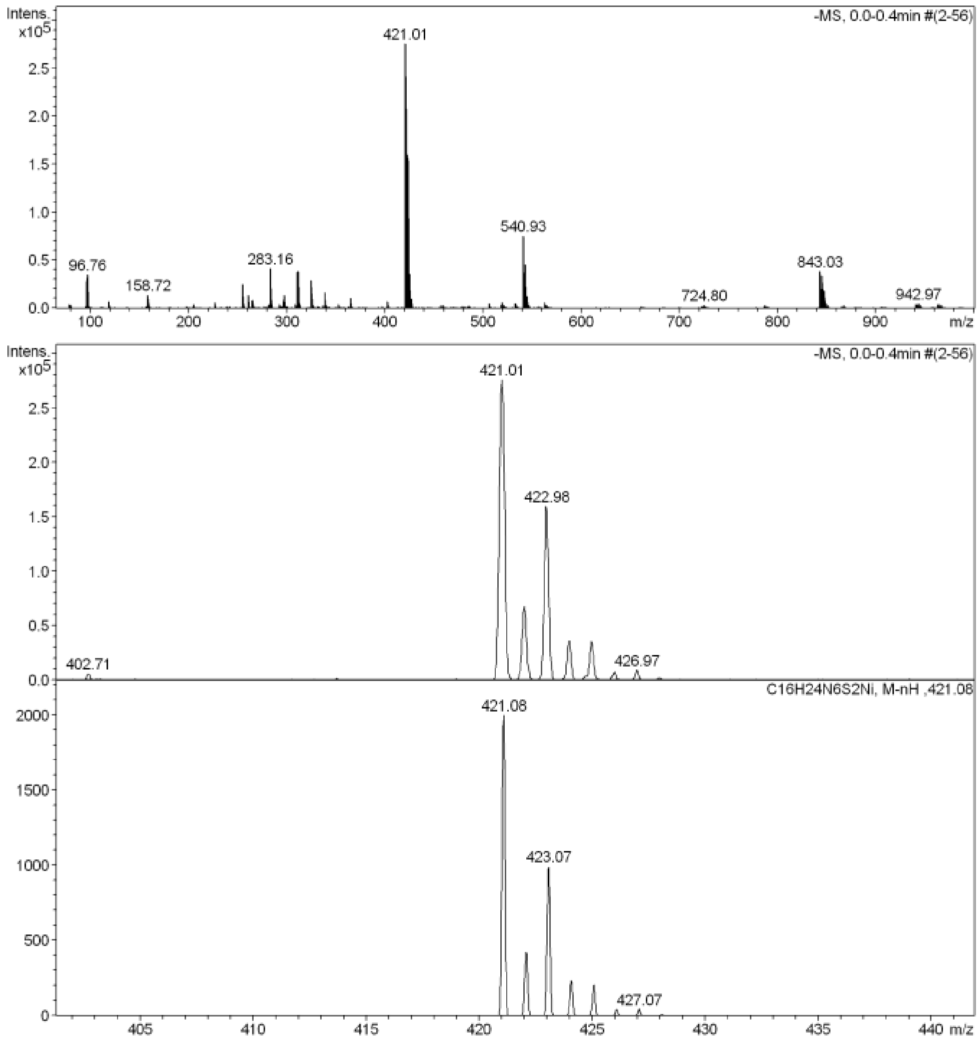

Figure S11. Negative ion ESI-MS for $\mathbf{Ni^{II}L^{S}}$.

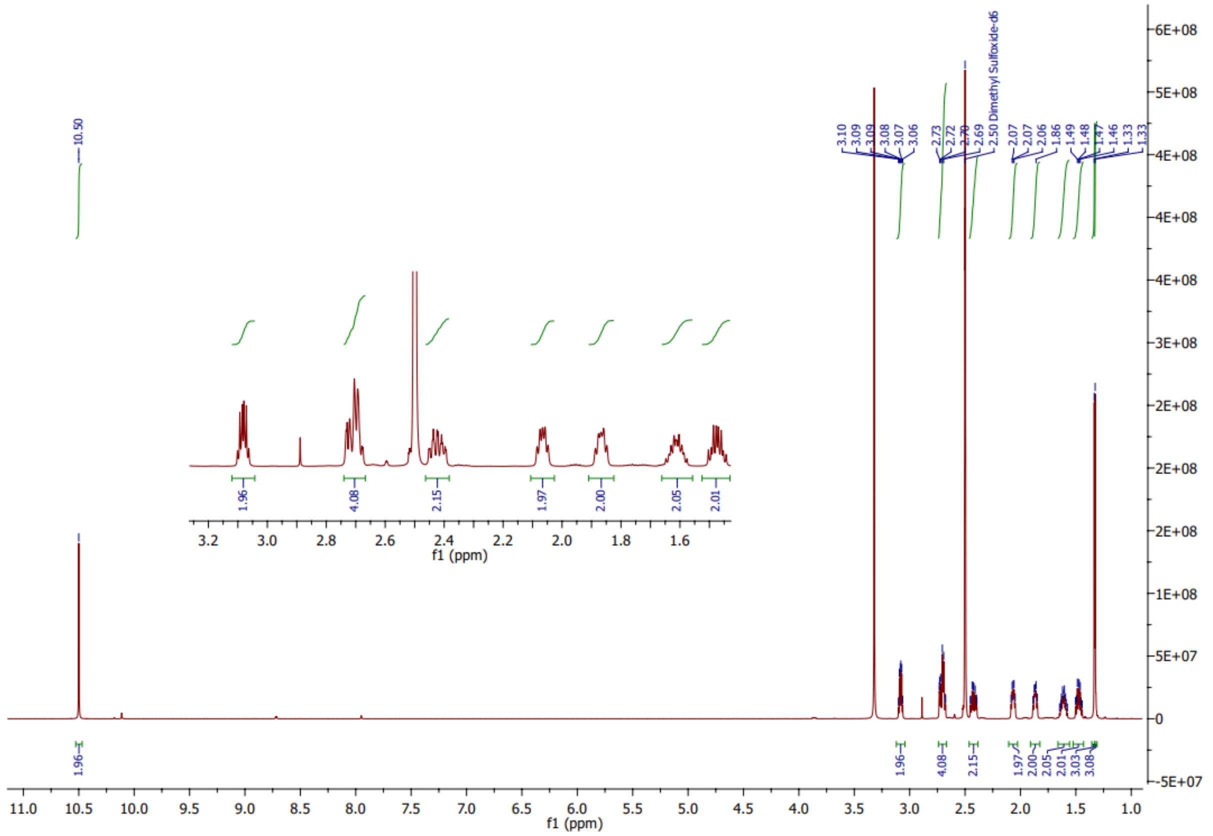

Figure S12. ^{1}H NMR spectra for $\mathbf{Ni^{II}L^{S}}$.

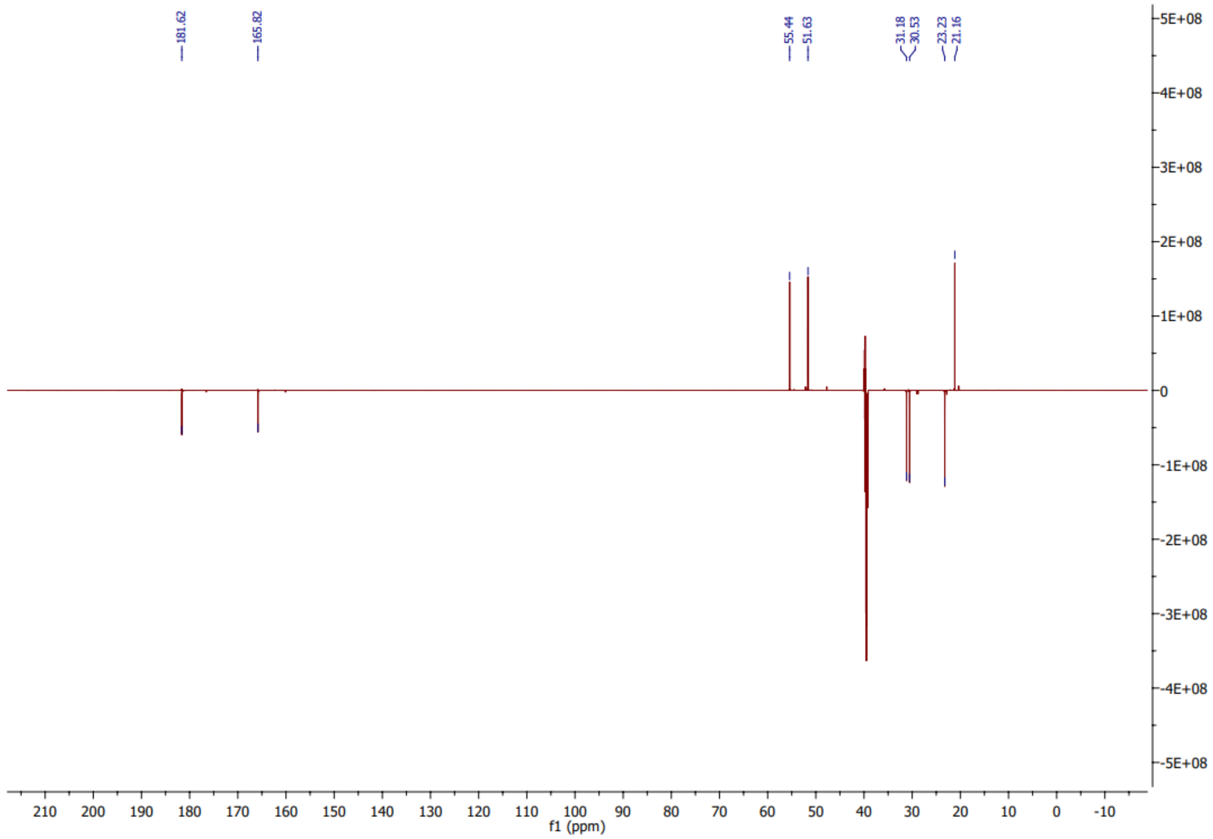

Figure S13. ^{13}C NMR spectra for $\mathbf{Ni^{II}L^{S}}$.

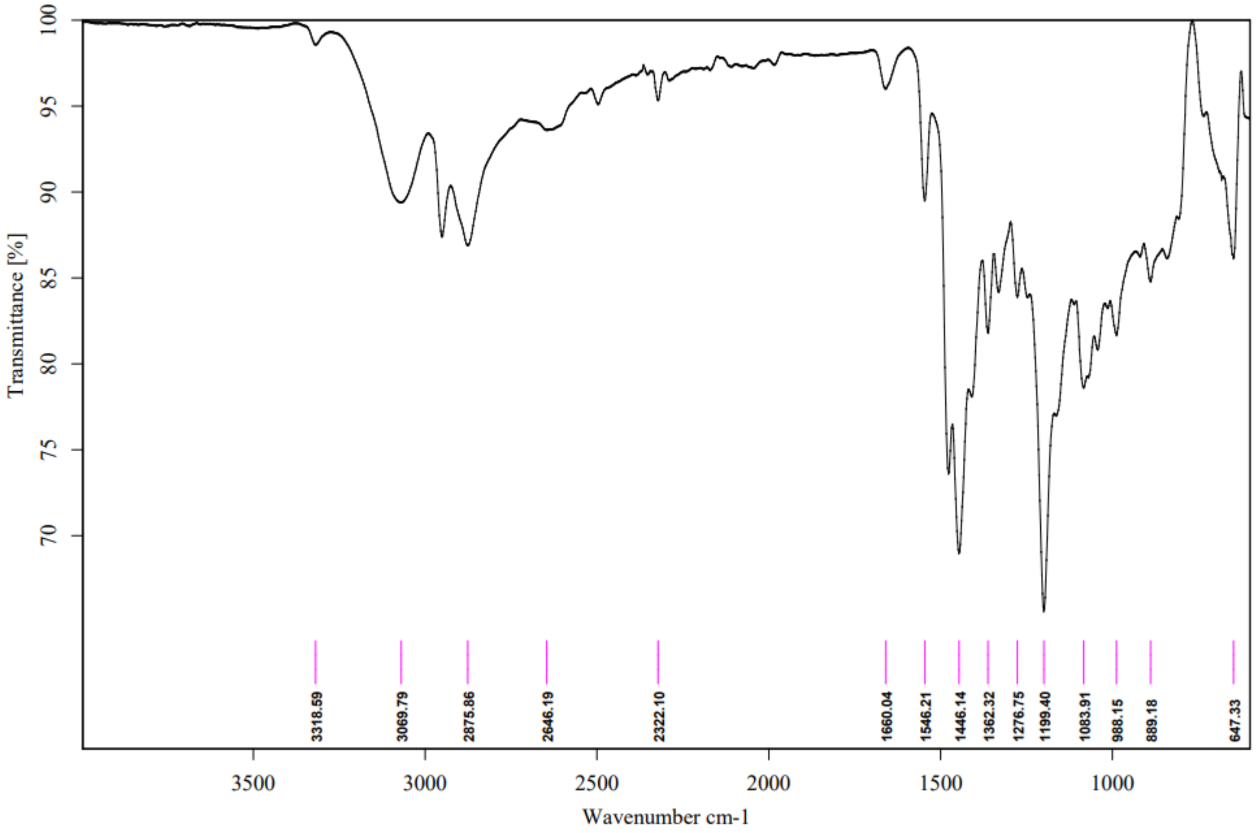

Figure S14. IR spectra for $\mathbf{Ni^{II}L^{S}}$.

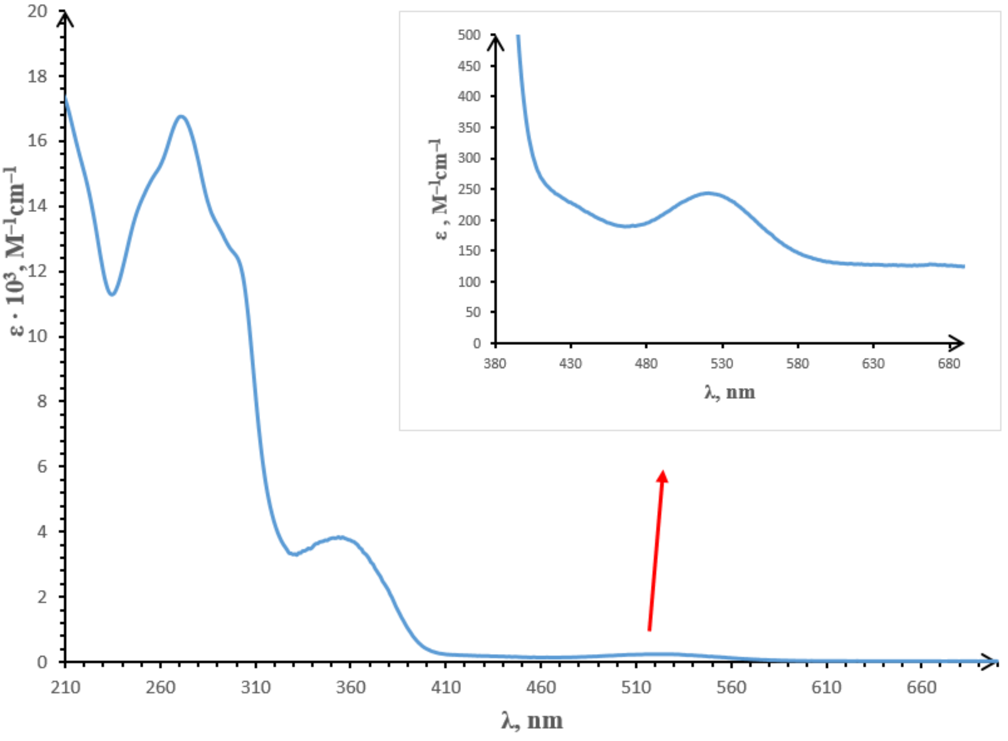

Figure S15. UV–vis absorption spectrum for $\mathbf{Ni^{II}L^{S}}$ in methanol.

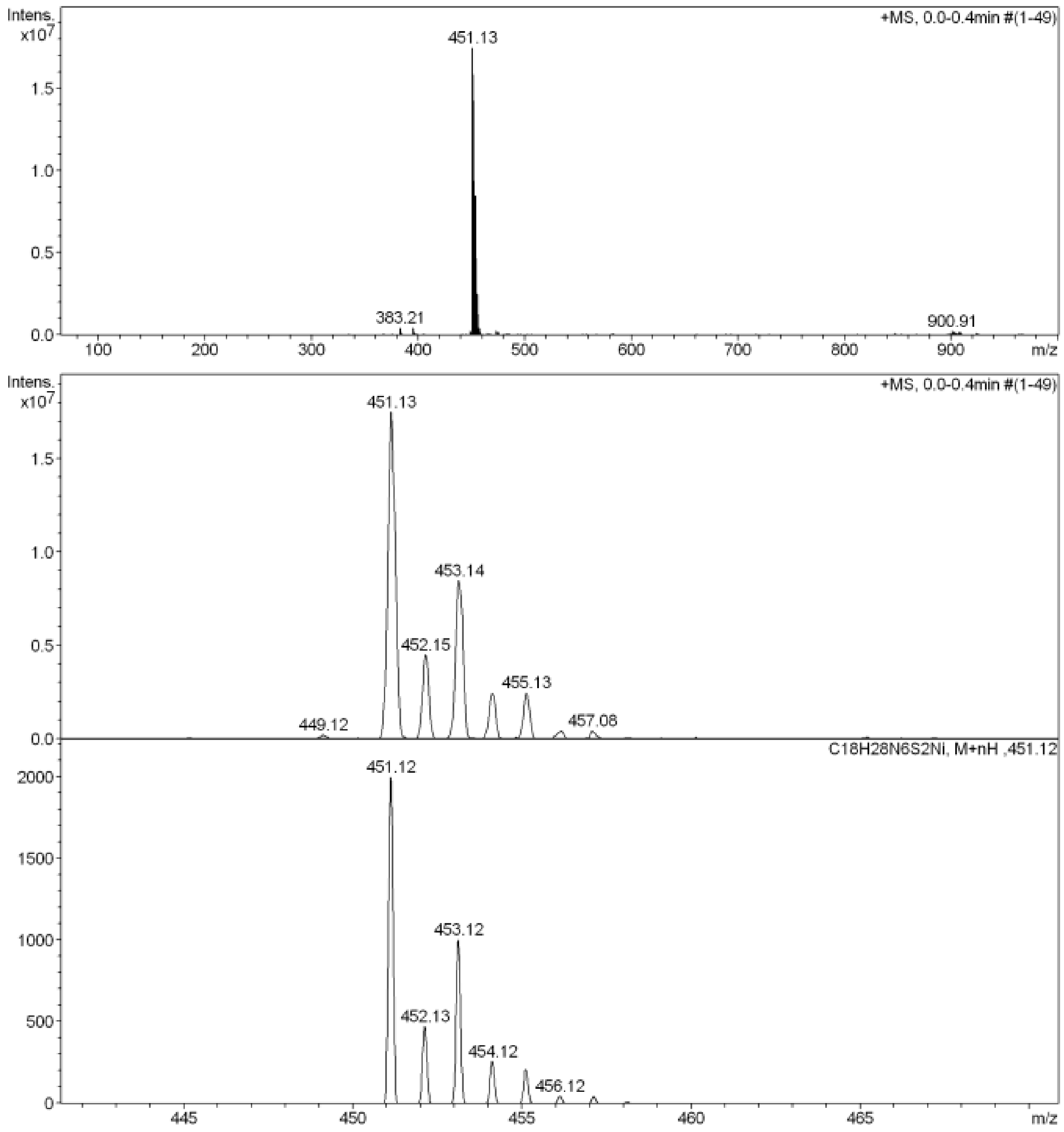

Figure S16. Positive ion ESI-MS for **Ni^{II}L^{SMe}**.

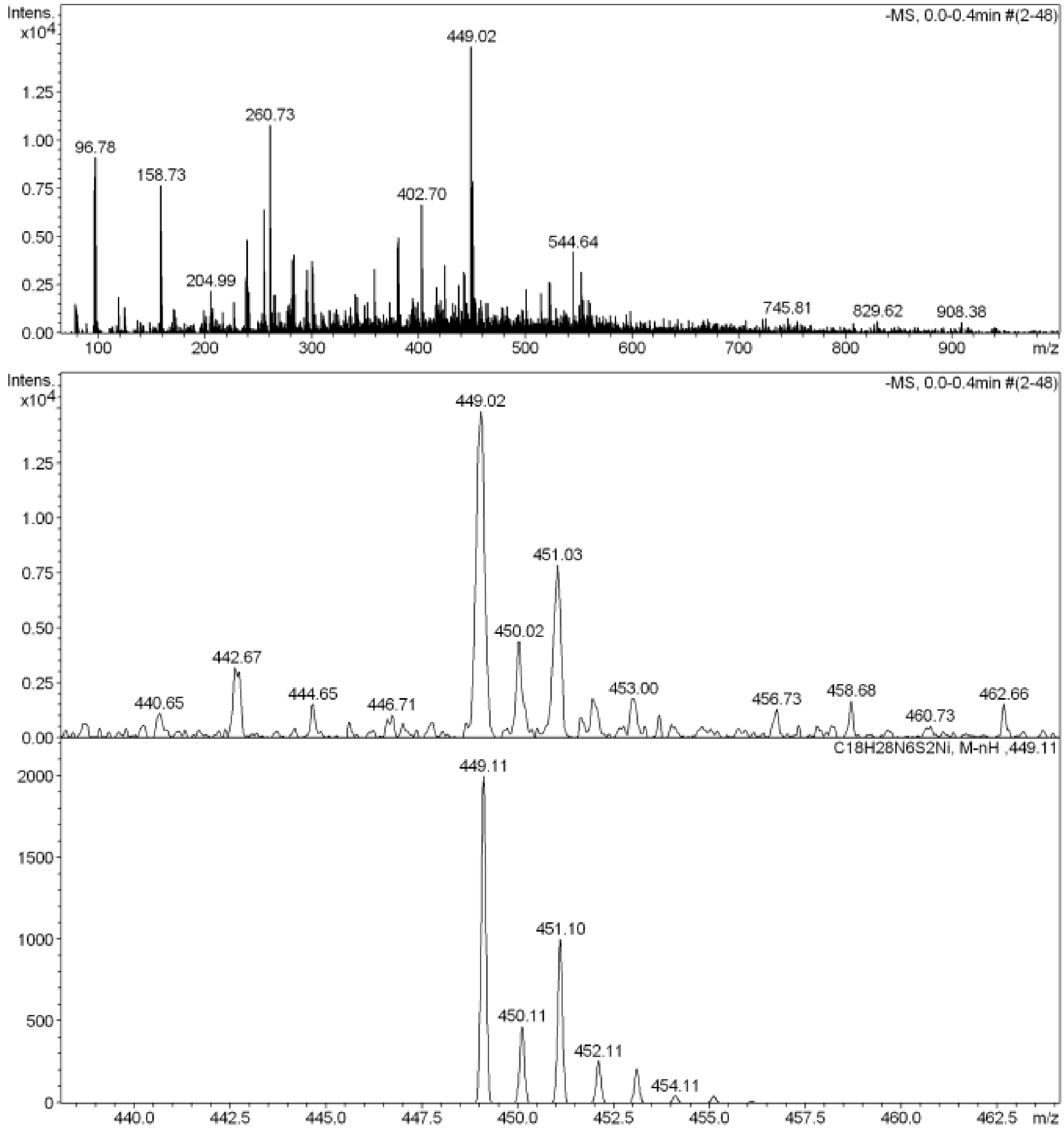

Figure S17. Negative ion ESI-MS for $\mathbf{Ni^{II}L^{SMe}}$.

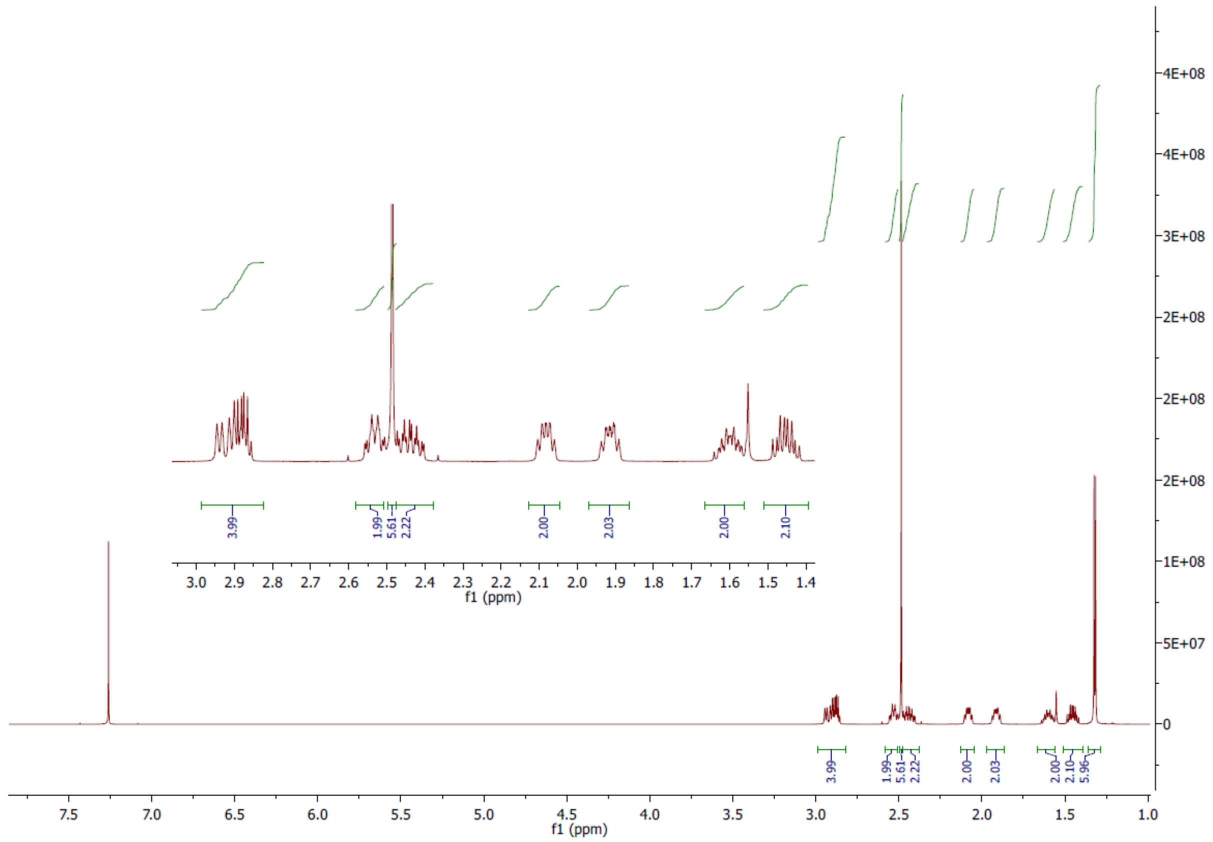

Figure S18. ^{1}H NMR spectra for $\mathbf{Ni^{II}L^{SMe}}$.

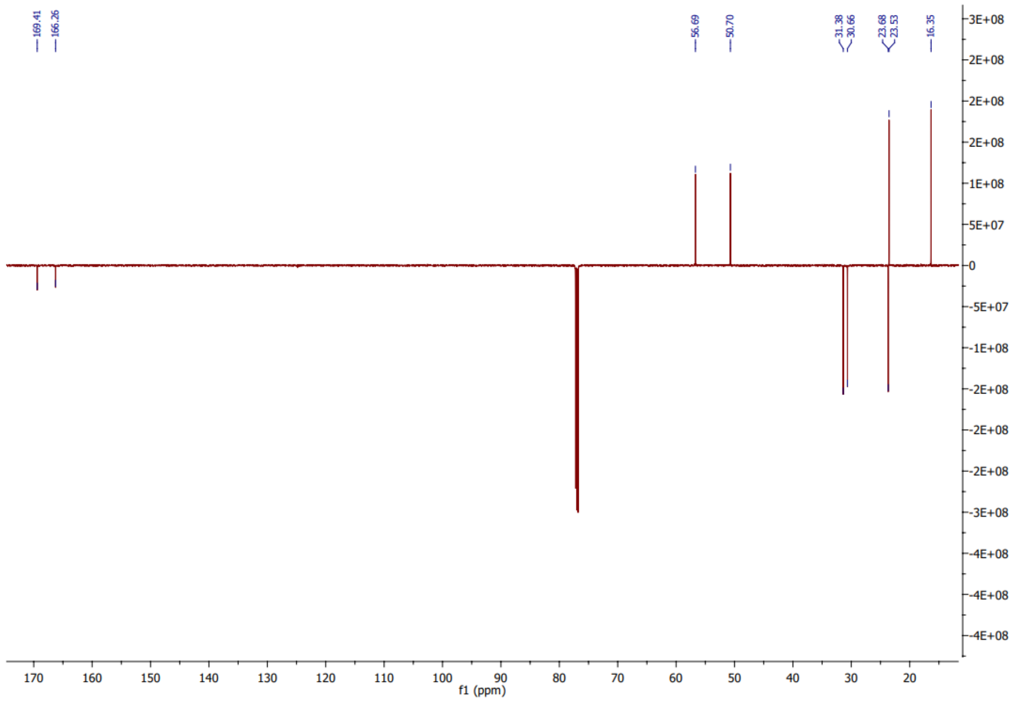

Figure S19. ^{13}C NMR spectrum for $\mathbf{Ni^{II}L^{SMe}}$.

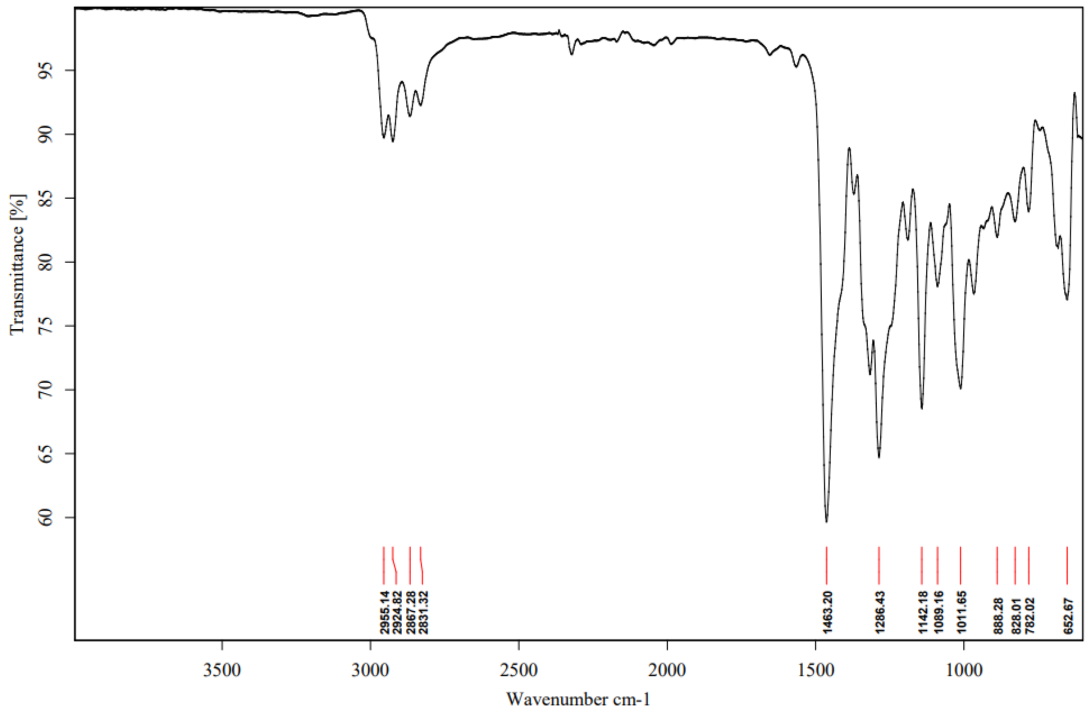

Figure S20. IR spectra for $\mathbf{Ni^{II}L^{SMe}}$.

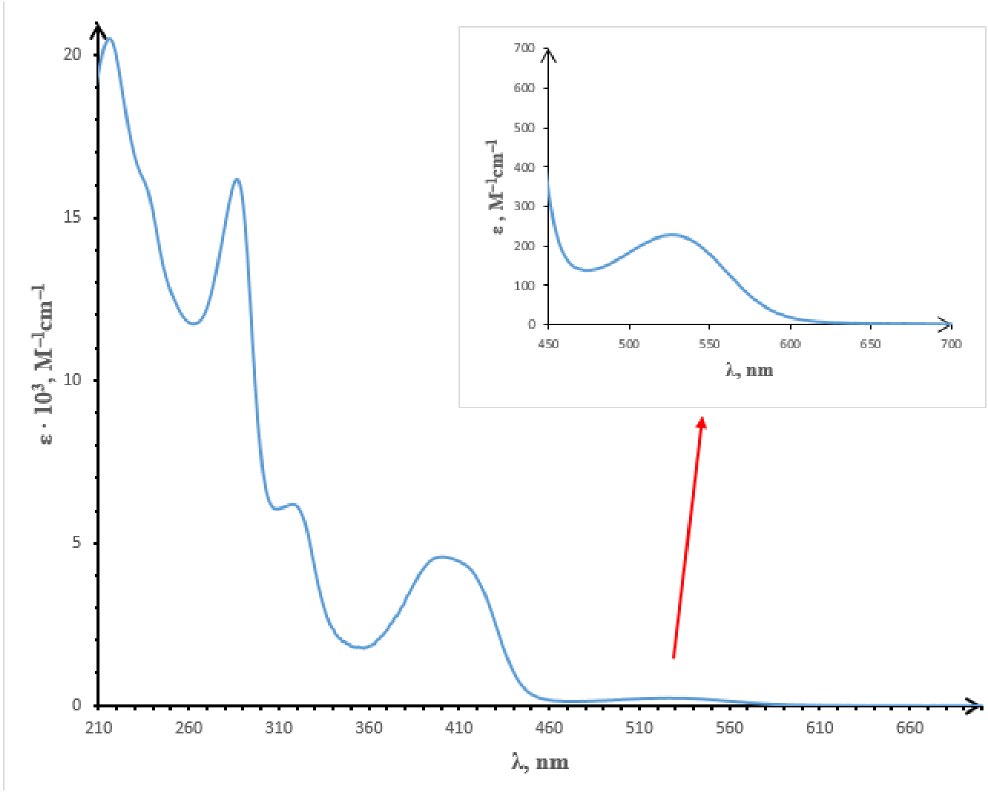

Figure S21. UV–vis absorption spectrum of $\mathbf{Ni^{II}L^{SMe}}$ in methanol.

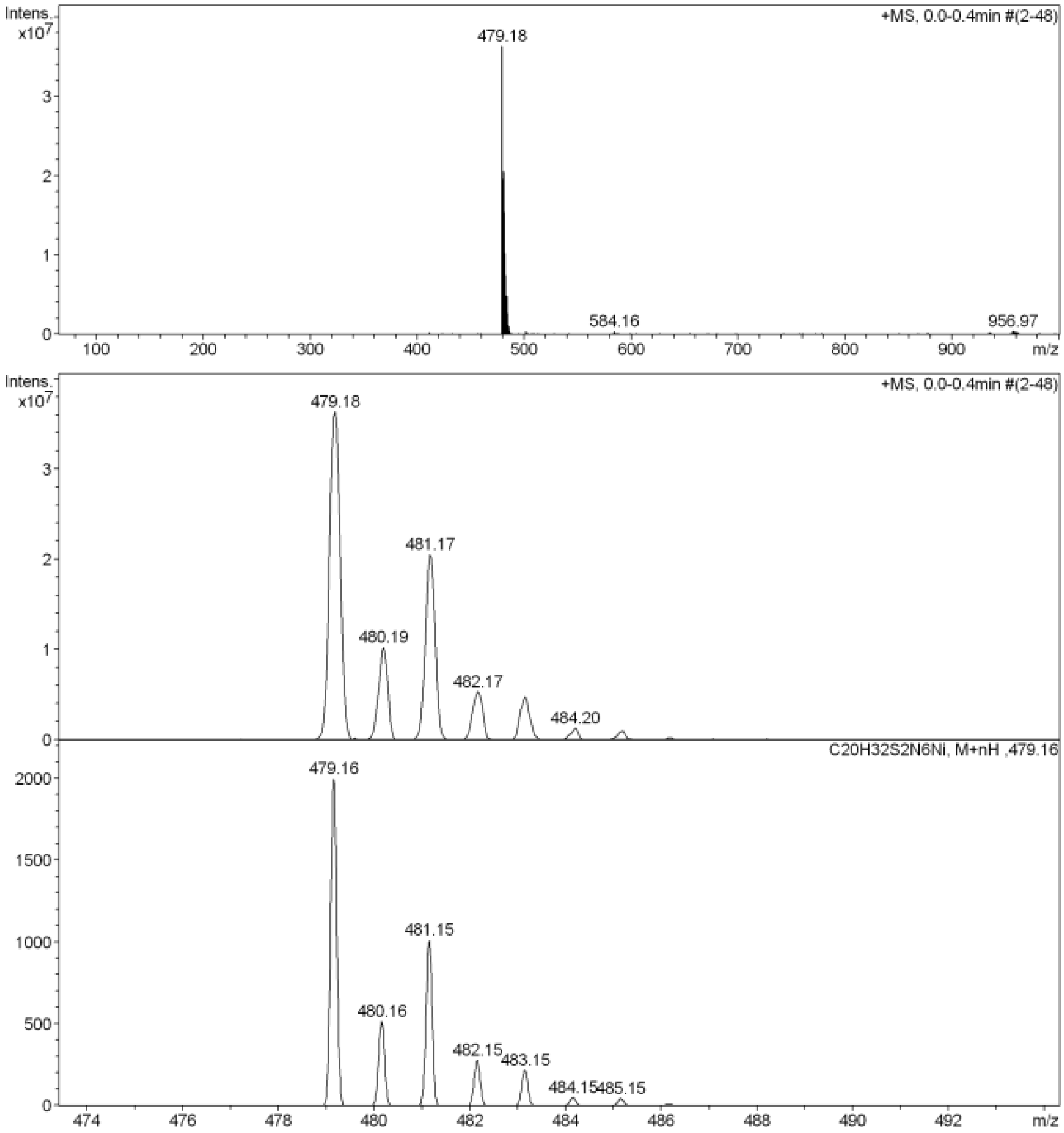

Figure S22. Positive ion ESI-MS for $\mathbf{Ni^{II}L^{SEt}}$.

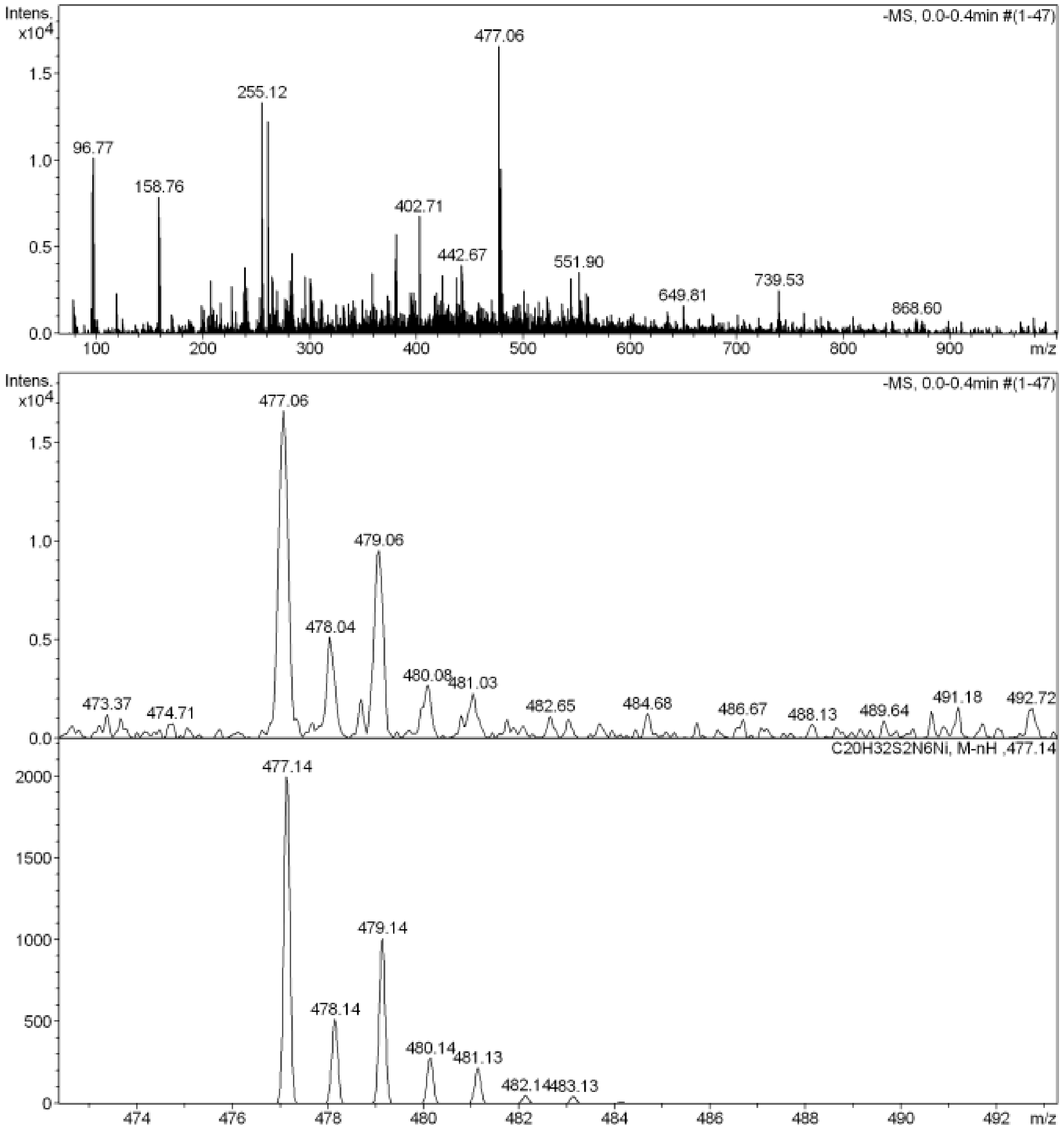

Figure S23. Negative ion ESI-MS for **Ni^{II}L^{SEt}**.

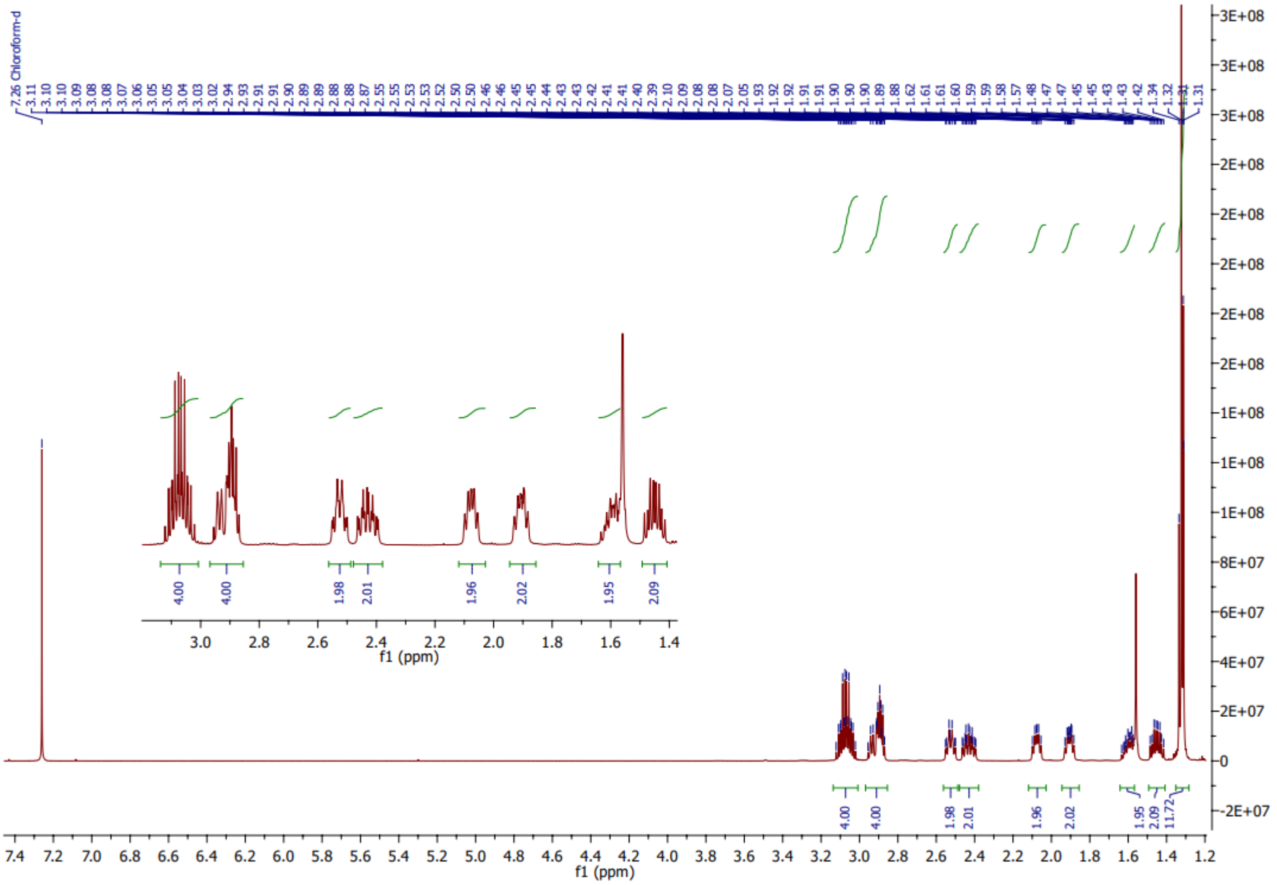

Figure S24. ^{1}H NMR spectrum for $\mathbf{Ni^{II}L^{SEt}}$.

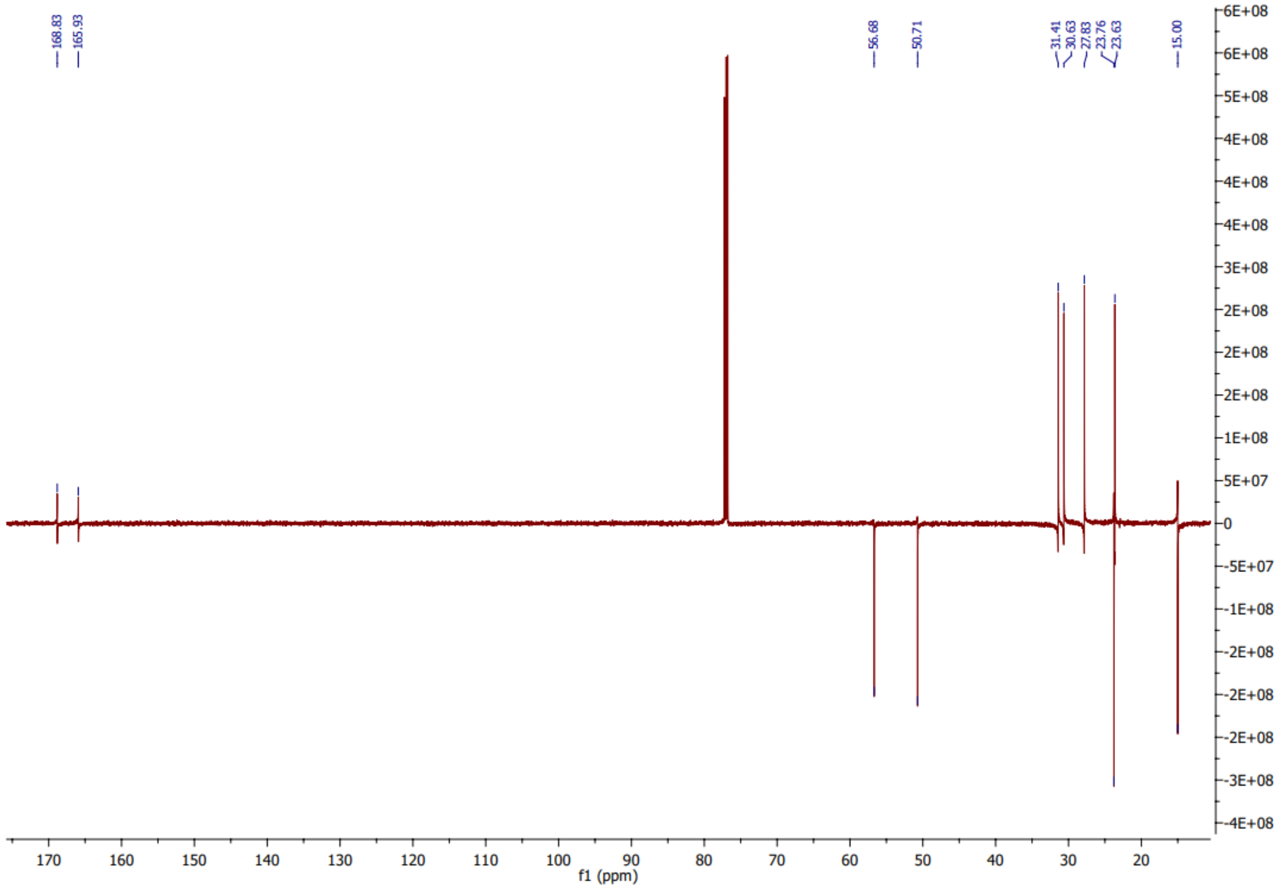

Figure S25. ^{13}C NMR spectra for **Ni^{II}L^{SEt}**.

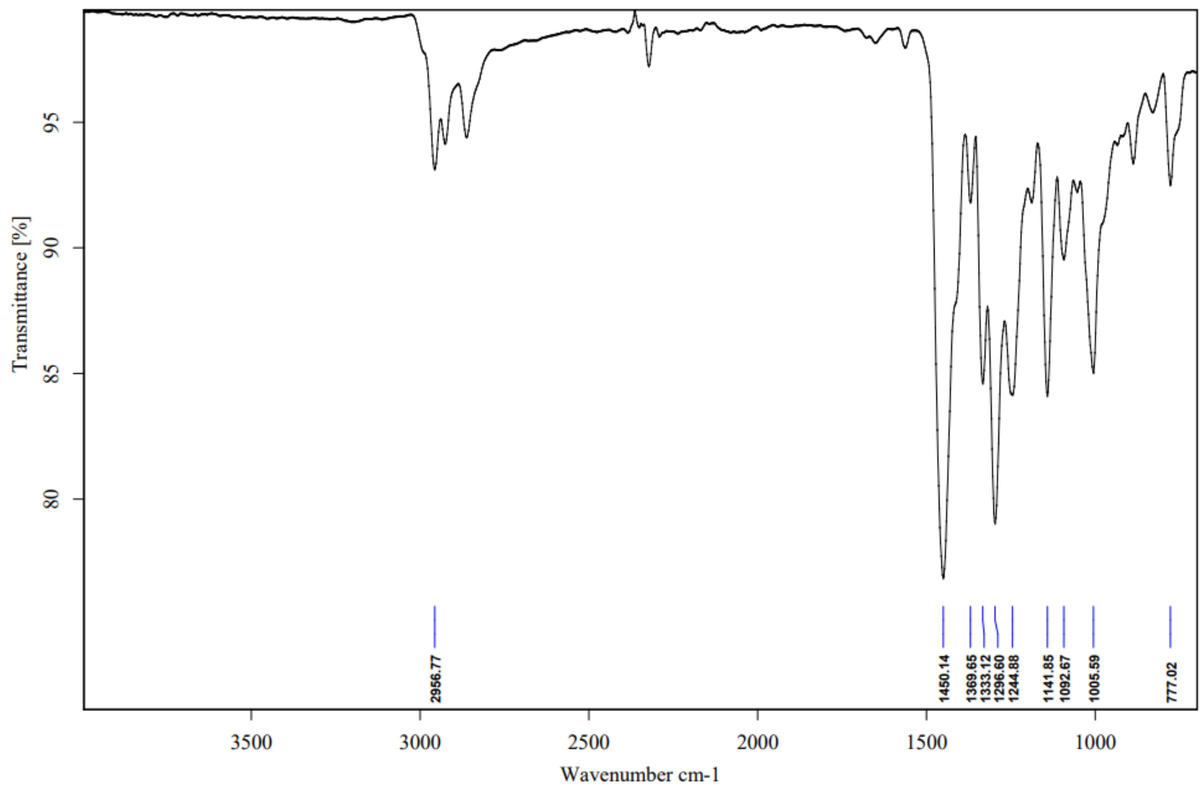

Figure S26. IR spectrum of **Ni^{II}L^{SEt}.**

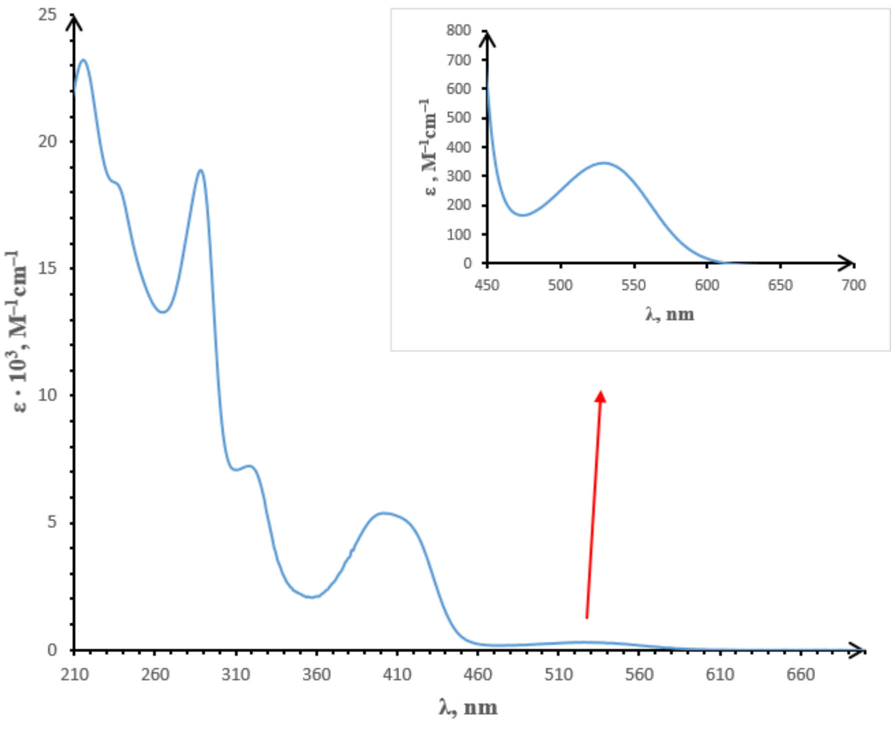

Figure S27. UV–vis absorption spectrum of **Ni^{II}L^{SEt}.**

3. Crystallographic data

Table S1. Deviation of atoms in 5-membered rings from respective NiN_2 planes in $\mathbf{Ni^{II}L^{S}}$.

Atom	**NiN1N2C3N4**	**NiN8N9C10N11**
N2/N9	–0.393(5)	–0.387(5)
C3/C10	–0.237(5)	–0.302(4)
Conformation	envelope	envelope

Table S2. Deviation of atoms in 6-membered rings from respective NiN_2 planes in $\mathbf{Ni^{II}L^{S}}$.

Atom	**NiN4C5C6C7N8**	**NiN11C12C13C14N1**
C5/C12	0.636(5)	0.393(5)
C6/C13	0.270(6)	0.874(5)
C7/C14	–0.145(5)	0.193(5)
C15/C16	2.155(5)	1.529(6)
Conformation	A	B

Table S3. Crystal Data and Details of Data Collection and Refinement for $\mathbf{H_2L^S}$, $\mathbf{H_2L^{SEt}}$, $\mathbf{NiL^S}$, $\mathbf{NiL^{SMe}}$ and $\mathbf{NiL^{SEt}}$.

compound	$\mathbf{H_2L^S}$	$\mathbf{H_2L^{SEt}}$	$\mathbf{NiL^S}$	$\mathbf{NiL^{SMe}}$	$\mathbf{NiL^{SEt}}$
empirical formula	$C_{16}H_{26}N_6S_2$	$C_{20}H_{34}N_6S_2$	$C_{16}H_{24}N_6NiS_2$	$C_{18}H_{28}N_6NiS_2$	$C_{20}H_{32}N_6NiS_2$
fw	366.55	422.65	423.24	451.29	479.34
space group	*Pbca*	*P*-1	*Cc*	$P2_1/c$	*P*-1
a, Å	17.0619(9)	9.2324(7)	17.4292(5)	11.5410(2)	11.6032(10)
b, Å	9.7583(3)	10.4294(7)	6.6235(3)	13.7657(2)	13.6885(12)
c, Å	21.8947(7)	12.5483(8)	16.2981(5)	13.0159(2)	16.1127(13)
α, °		101.269(5)			112.018(3)
β, °		103.562(6)	100.416(2)	103.9050(10)	90.826(3)
γ, °		93.900(6)			107.495(3)
V [Å^3]	3645.4(3)	1143.68(14)	1850.48(11)	2007.24(6)	2239.3(3)
Z	8	2	4	4	2
λ [Å]	0.71073	1.54186	0.71073	0.71073	0.71073
ρ_{calcd}, g cm^{-3}	1.336	1.227	1.519	1.493	1.422
cryst size, mm^3	0.10 × 0.08 × 0.03	0.13 × 0.11 × 0.11	0.24 × 0.15 × 0.07	0.31 × 0.18 × 0.14	0.25 × 0.10 × 0.05
T [K]	100(2)	300(2)	100(2)	100(2)	200(2)
μ, mm^{-1}	0.303	2.238	1.286	1.191	1.072
$R_1{}^a$	0.0282	0.0488	0.0292	0.0254	0.0362
$wR_2{}^b$	0.0739	0.1456	0.0679	0.0639	0.0869
GOF^c	1.038	1.056	1.089	1.021	1.010
CCDC no.	2324332	2324333	2324334	2324335	2324336

^{a} $R_1 = \Sigma||F_o| - |F_c||/\Sigma|F_o|$. ^{b} $wR_2 = \{\Sigma[w(F_o^2 - F_c^2)^2]/\Sigma[w(F_o^2)^2]\}^{1/2}$. ^{c} $GOF = \{\Sigma[w(F_o^2 - F_c^2)^2]/(n - p)\}^{1/2}$, where n is the number of reflections and p is the total number of parameters refined.

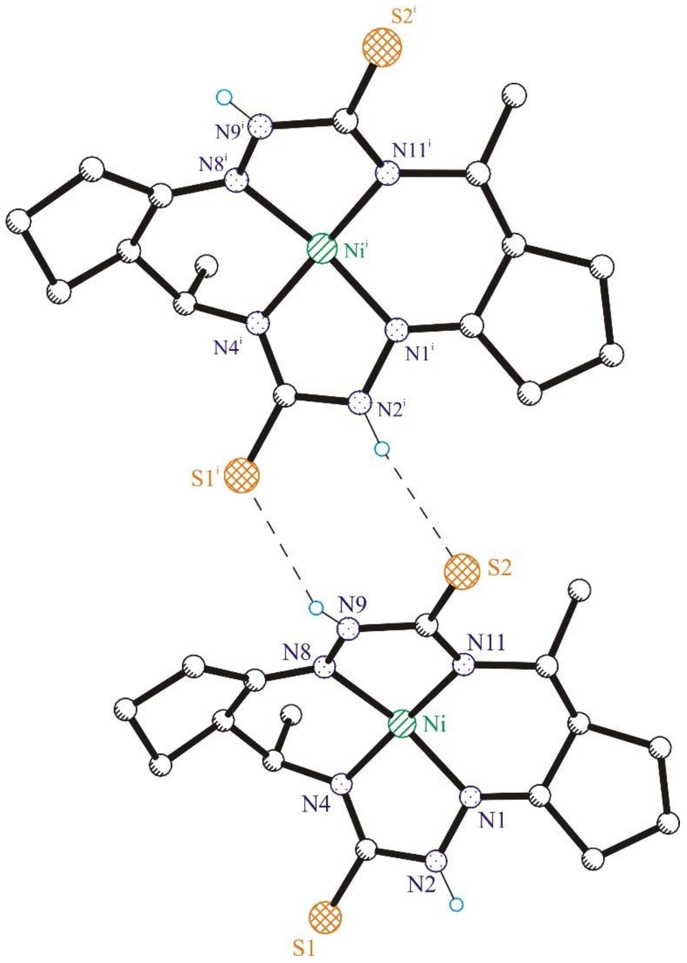

Figure S28. Formation of dimeric associates in the crystal of $\mathbf{Ni^{II}L^{S}}$ via two intermolecular hydrogen bonding interactions of the thiolactam group of the type N9–H···S1^{i} and N2^{i}–H···S2. Symmetry code: (i) *x* – 0.5, *y* – 0.5, +*z*.
